# Exploring ChatGPT effectiveness in addressing direct patient queries on colorectal cancer screening

**DOI:** 10.1055/a-2568-9416

**Published:** 2025-05-12

**Authors:** Marcello Maida, Yuichi Mori, Lorenzo Fuccio, Sandro Sferrazza, Alessandro Vitello, Antonio Facciorusso, Cesare Hassan

**Affiliations:** 1 217140 Department of Medicine and Surgery, Kore University of Enna, Enna, Italy; 2 73129 Gastroenterology Unit, Umberto I Hospital, Enna, Italy; 3 6305 Institute of Health and Society, University of Oslo, Oslo, Norway; 4 220878 Digestive Disease Center, Showa University Northern Yokohama Hospital, Yokohama, Japan; 5 18508 Department of Medical and Surgical Sciences, IRCCS University Hospital of Bologna Sant Orsola Polyclinic, Bologna, Italy; 6 9296 Department of Medical and Surgical Sciences, University of Bologna, Bologna, Italy; 7 26204 Gastroenterology Unit, Ospedale Civico Palermo, Palermo, Italy; 8 18972 Gastroenterology Unit, Department of Experimental Medicine, University of Salento, Lecce, Italy; 9 6305 Clinical Effectiveness Research Group, University of Oslo, Oslo, Norway; 10 437807 Department of Biomedical Sciences, Humanitas University, Milan, Italy; 11 9268 Endoscopy Unit, IRCCS Humanitas Research Hospital, Rozzano, Italy

**Keywords:** Endoscopy Lower GI Tract, CRC screening, Colorectal cancer

## Abstract

**Background and study aims:**

Recent studies showed that large language models (LLMs) could enhance understanding of colorectal cancer (CRC) screening, potentially increasing participation rates. However, a limitation of these studies is that questions posed to LLMs are generated by experts. This study aimed to investigate ChatGPT-4o effectiveness in answering CRC screening queries directly generated by patients.

**Patients and methods:**

Ten consecutive subjects aged 50 to 69 years who were eligible for the Italian national CRC screening program but not actively involved were enrolled. Four possible scenarios for CRC screening were presented to each participant and they were asked to formulate one question per scenario to gather additional information. These questions were then posed to ChatGPT in two separate sessions. The responses were evaluated by five senior experts, who rated each answer based on three criteria: accuracy, completeness, and comprehensibility, using a 5-point Likert scale. In addition, the same 10 patients who created the questions assessed the answers, rating each response as complete, understandable, and trustworthy on a dichotomous scale (yes/no).

**Results:**

Experts rated the responses with mean scores of 4.1 ± 1.0 for accuracy, 4.2 ± 1.0 for completeness, and 4.3 ± 1.0 for comprehensibility. Patients rated responses as complete in 97.5%, understandable in 95%, and trustworthy in 100% of cases. Consistency over time was confirmed by an 86.8% similarity between session responses.

**Conclusions:**

Despite variability in questions and answers, ChatGPT confirmed good performances in answering CRC screening queries, even when used directly by patients.

## Graphical Abstract

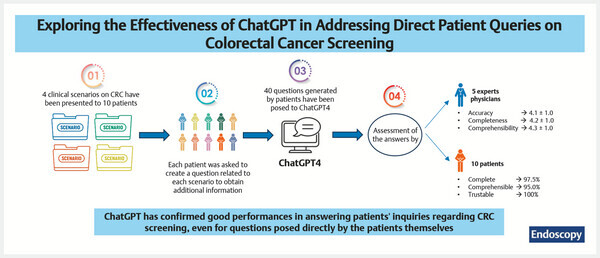

## Introduction

Colorectal cancer (CRC) remains a major global health issue, standing as the third most common cancer in men, the second in females, and the fourth leading cause of cancer-related deaths worldwide 1 . CRC screening through early identification and resection of precancerous and cancerous lesions has a crucial role in decreasing disease incidence and mortality 2 3 , and participation rates significantly influence effectiveness of screening programs.

Unfortunately, the current participation rate is limited to approximately 30% to 80% 4 5 .

Studies have shown that one contributing factor to the low participation rate is lack of knowledge about screening programs, largely due to insufficient opportunities for participants to learn about them 6 7 . To address this issue, large language models (LLMs) could help improve understanding of CRC screening programs, ultimately increasing participation rates and enhancing the impact of cancer prevention.

In a recent study, we demonstrated that ChatGPT-4 performs well in answering questions about CRC screening 8 . Nevertheless, a key limitation in that study, as well as in others evaluating the effectiveness of LLMs, was that common questions asked by patients were generated by experts. On the other hand, when patients ask questions firsthand, the latter may be less focused, affecting the quality of the responses.

To overcome this limitation, we conducted this prospective study aimed at assessing the performance of ChatGPT-4 (Chat Generative Pretrained Transformer, OpenAI Foundation) 9 in responding to questions directly asked by patients.

## Patients and methods

A team of three national experts with documented clinical and scientific experience on CRC screening was selected by the authors and invited to participate in the project. The working group met regularly through videoconference meetings and developed four clinical scenarios related to CRC:

- **Scenario I:** You've heard about colon cancer screening and want more information. Please ask your question for clarification.
- **Scenario II:** You want to participate in colon cancer screening but are unsure how to get started. Please ask your question for guidance.
- **Scenario III:** You've received a positive fecal occult blood test result but are uncertain about its implications and what other tests you may need. Ask your question for clarification.
- **Scenario IV:** Following a positive fecal occult blood test result, you have been advised to undergo a colonoscopy, and you are unsure about the procedure and how to prepare for it. Please ask your question for detailed information.

The scenarios were constructed on the most common concerns expressed by patients undergoing screening, based on data from the literature 10 and our center experience.

Therefore, we enrolled 10 consecutive subjects aged 50 to 69 years who were eligible for the Italian national CRC screening program but not actively involved (e.g. never receiving a positive fecal immunohistochemical test [FIT] result or a colonoscopy after a positive FIT). We asked each patient to create a question related to each scenario to obtain additional information. Participants were also asked to recall the keywords of each scenario in the questions to provide context.

Thereafter, the same queries were posed to ChatGPT (version ChatGPT-4o) in two separate sessions (August 30, 2024, and September 14, 2024), and the responses were recorded.

For example, in Scenario 1, patients asked, “What does colon cancer screening mean?” The response from ChatGPT was: “Colon cancer screening refers to a set of tests and procedures used to detect colorectal cancer in people who have no symptoms, the goal is to detect cancer at an early stage, when is more treatable and has better chances of cure, or identify and remove precancerous polyps before they turn into malignant tumors” ( **Supplementary Table 1** ).

In a second phase, five senior experts with extensive experience with and knowledge about CRC and cancer screening programs reviewed the responses. They scored each response according to three domains - accuracy, completeness, and comprehensibility - using a 5-point Likert scale ( **Supplementary Table 2** ).

The answers were also scored by the same 10 patients who formulated the questions, who rated each response dichotomously (yes/no) as complete, understandable, and trustable.

Both experts and patients performed their ratings independently, blinded to the evaluations of the other group. All questions and answers were formulated in Italian, the native language of patients and raters, to ensure greater accuracy.

Because neither clinical patient data nor intervention approaches were used, Institutional Review Board approval was not required. The results were analyzed using mean ± standard deviation for reporting continuous variables and frequency and percentage for categorical variables.

Internal consistency of the scale was assessed using the Cronbach alpha coefficient and cut-off points of < 60%, 61% to 70%, 71% to 80%, 81% to 90%, and > 90% were considered to suggest poor, questionable, acceptable, good, and excellent reliability, respectively 11 .

Degree of similarity in responses between the two sessions was evaluated using a similarity detection software (GoTranscript, GoTranscript Inc., Lewes, Delaware, United States). All statistical analyses were performed using SPSS v. 29.0 for Macintosh (SPSS Inc., Chicago, Illinois, United States).

## Results

All 10 enrolled subjects were native Italian speakers. Mean age was 60 ± 5.6 years, with 60% being male. Educational status was elementary school (10%), middle school (20%), high school (30%), and academic (30%). Among these, only two subjects reported previous exposure to LLMs systems.

**Fig. 1:**
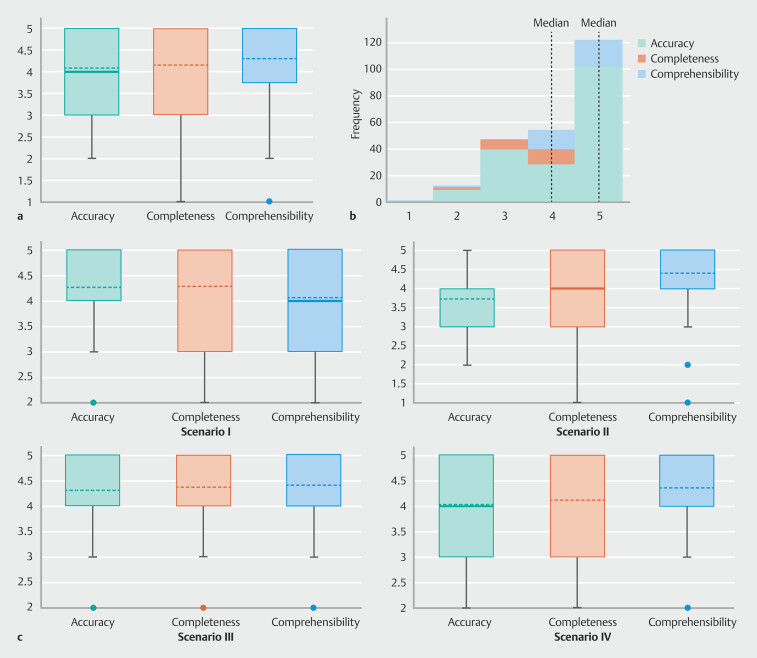
**a** Box plot and **b** frequency histogram showing the rating of ChatGPT answers by experts overall, and **c** in each scenario. (Full list of Q&A for each scenario available in Supplementary Table 1 and scales for assessment of accuracy, completeness and comprehensibility are available in Supplementary Table 2).

According to the expert rating, mean accuracy, completeness and comprehensibility scores of ChatGPT answers were 4.1 ± 1.0, 4.2 ± 1.0, and 4.3 ± 1.0, respectively ( Fig. 1 **a, 1b** ).

In detail, the highest accuracy score was registered in Scenarios 1 and 3 (4.3 ± 0.9), while the lowest was in Scenario 2 (3.7 ± 0.9). The highest completeness rating score was registered in Scenario 1 (4.3 ± 1.0) and Scenario 3 (4.4 ± 0.9), whereas the lowest was in Scenario 2 (3.8 ± 1.0).

Moreover, a high comprehensibility rating > 4 was recorded in all scenarios ( Fig. 1 **c** ).

The Cronbach alpha coefficient (α = 0.9) showed an excellent internal consistency among expert ratings.

Concerning patient assessment, all questions were deemed complete in 97.5%, understandable in 95%, and trustworthy in 100% of cases.

Finally, we evaluated text similarity in each pair of responses obtained by ChatGPT in the first and second sessions. The results showed an average similarity of 86.8 ± 2.7% (range 82%-93%), indicating good consistency of outputs over time ( **Supplementary Table 1** ).

## Discussion

Results from this study confirm the good performance of ChatGPT-4o in assisting patients with CRC screening information, with high levels of accuracy, completeness, and comprehensibility.

However, we found a significant diversity in the questions asked in the same scenario, resulting in a wide range of generated answers. In addition, we observed a 13% average difference between the answers provided by ChatGPT-4o in the first and second sessions.

As is known, consistency in LLM-generated responses is crucial to avoid patient confusion and misinformation. The literature does not provide data demonstrating when a level of consistency can be defined acceptable in a medical setting. However, strategies such as Prompt Engineering, Supervised Fine-Tuning, or Retrieval-Augmented Generation may be considered to increase accuracy and mitigate inconsistencies 12 .

In our study, despite a slight variability in responses, we found that ChatGPT-4o is still effective in providing accurate, complete, and understandable answers. Moreover, patients provided positive feedback about the completeness, comprehensibility, and trustworthiness of the responses, indicating their favorable perception of tool performance.

Nevertheless, it is important to emphasize that this technology is not intended to replace professional medical advice. Most patients require face-to-face interactions with healthcare providers to discuss their concerns and receive necessary explanations. In addition, consulting a doctor is always needed to address complex issues involving health conditions and medication management and to provide personalized healthcare solutions.

Future research should investigate integration of LLMs into patient education with physician oversight to ensure safe and effective utilization.

To the best of our knowledge, this is the first study assessing the performance of a LLM in responding to CRC screening-related inquiries directly formulated by patients.

The study has several strengths. First, the questions were created directly by patients and evaluation of responses was conducted by both experts and patients. Second, queries were posed to ChatGPT-4o in two separate sessions to assess consistency of responses over time. Finally, the evaluation was conducted in the native language of the patients and experts to ensure consistency.

This study also presents some limitations. First, we assessed patient queries through a unique LLM tool, namely ChatGPT. This approach was taken because this study serves as a follow-up to previous research that validated the efficacy of ChatGPT in addressing patient concerns through queries generated by experts. As a consequence, it was crucial to utilize the same tool to ensure consistent comparisons of results. Nevertheless, even if comparing different AI tools is beyond the scope of this study, further research on the performance of different LLMs in this context would be useful. Second, the study was limited to 10 patients enrolled and four scenarios, resulting in creation of 40 questions and answers, and the processing of 600 ratings. In the future, studies with larger sample sizes. would be beneficial to validate these findings. Finally the study was conducted exclusively in Italian, which may affect the accuracy and comprehensiveness of the responses generated by LLMs and limits generalizability of results. Therefore, future validation studies in different languages and cultural contexts are necessary to confirm these findings.

## Conclusions

In conclusion, ChatGPT has confirmed good performances in answering patient inquiries regarding CRC screening, even for questions posed directly by the patients themselves. In the future, enhancing this tool would be beneficial by ensuring greater consistency in responses and better contextualization of information based on region and country. Furthermore, upcoming studies evaluating LLMs should focus more on questions posed by patients to ensure a more accurate assessment of the tool.

## Supplementary Material

Supplementary Material Zusatzmaterial
