# Supplementary material for: Exploring ChatGPT effectiveness in addressing direct patient queries on colorectal cancer screening
Source: Endosc Int Open. 2025 May 12;13:a25689416. doi: 10.1055/a-2568-9416 (PMC12080512; doi:10.1055/a-2568-9416)
Supplement: Supplementary file 1 — Supplementary Material [file 10-1055-a-2568-9416_25704776.pdf]

Supplementary Table 1. Questions Posed to ChatGPT and their respective answers (reported in original language and English translation).

ORIGINAL LANGUAGE (ITALIAN)

SCENARIO 1

Hai sentito parlare dello screening per il cancro del colon e vuoi ricevere maggiori informazioni. Fai la tua domanda per ricevere chiarimenti.

1) **INPUT: Che significa screening del tumore del colon?**

OUTPUT (SESSION 1): Lo screening del tumore del colon si riferisce a un insieme di test e procedure utilizzati per rilevare il cancro del colon-retto in persone che non presentano sintomi. L'obiettivo è individuare il cancro in una fase precoce, quando è più trattabile e le possibilità di cura sono migliori, oppure identificare e rimuovere polipi precancerosi prima che si trasformino in tumori maligni.
Esistono diversi tipi di test di screening per il tumore del colon, tra cui:

1. Colonscopia: Una procedura in cui un medico utilizza un endoscopio per esaminare l'interno del colon e del retto. Durante la colonscopia, è possibile rimuovere polipi o prelevare campioni di tessuto per ulteriori analisi.
2. Colonscopia virtuale (o colonografia TC): Un esame radiologico che utilizza tomografia computerizzata per ottenere immagini del colon.
3. Sigmoidoscopia flessibile: Simile alla colonscopia ma limita l'esame all'ultima parte del colon.
4. Test del sangue occulto nelle feci (FOBT) o test immunochimico fecale (FIT): Questi test cercano tracce di sangue nelle feci, un possibile indicatore di cancro o polipi.
5. Test di DNA fecale (es. Cologuard): Cerca alterazioni genetiche nelle cellule di sfaldamento del colon che passano attraverso le feci, oltre a tracce di sangue.

La scelta del test dipende da vari fattori tra cui l'età, la storia familiare di cancro, i fattori di rischio personali, e le preferenze del paziente. È importante consultare un medico per decidere il programma di screening più opportuno.

OUTPUT (SESSION 2): Lo screening del tumore del colon è un insieme di test e procedure mediche utilizzate per rilevare il cancro del colon-retto in persone che non presentano sintomi. L'obiettivo è identificare eventuali polipi precancerosi o tumori in fase precoce, quando sono più facilmente trattabili. I test comuni per lo screening includono:

1. Colonscopia: un esame visivo diretto dell'interno del colon usando un tubo flessibile con una telecamera.
2. Sigmoidoscopia flessibile: simile alla colonscopia ma esamina solo la parte inferiore del colon.
3. Ricerca di sangue occulto nelle feci (FOBT): un test che rileva tracce di sangue nelle feci, che possono indicare la presenza di polipi o tumori.
4. Test del DNA fecale: analizza le feci per rilevare mutazioni genetiche associate al cancro del colon.
5. Colonscopia virtuale (colonografia TC): una scansione TC che produce immagini dettagliate del colon e del retto.

Lo screening regolare è raccomandato generalmente a partire dai 45-50 anni, ma può essere iniziato prima per chi ha fattori di rischio elevato. È importante discutere

con un medico per determinare quale test sia più appropriato basato sulla storia personale e familiare.

Text similarity: 87%

2) **INPUT: Cosa si intende per screening del tumore al colon?**

OUTPUT (SESSION 1): Lo screening del tumore al colon si riferisce a una serie di test medici progettati per rilevare precocemente il cancro del colon-retto nelle persone che non presentano sintomi. L'obiettivo degli screening è individuare il cancro in una fase iniziale, quando il trattamento è più efficace e le probabilità di successo sono maggiori. Inoltre, lo screening aiuta a scoprire e rimuovere polipi precancerosi prima che possano trasformarsi in tumori maligni.
Ecco i principali metodi di screening usati:

1. Colonscopia: Esamina l'intero colon e il retto usando un tubo flessibile con una telecamera. Può individuare e rimuovere i polipi.
2. Colonscopia virtuale (colonografia TC): Utilizza la tomografia computerizzata per creare immagini dettagliate del colon.
3. Sigmoidoscopia flessibile: Esamina solo la parte inferiore del colon e del retto.
4. Test del sangue occulto nelle feci (FOBT) e test immunochimico fecale (FIT): Rilevano il sangue nelle feci, che può indicare la presenza di polipi o cancro.
5. Test di DNA fecale: Analizza le feci per rilevare alterazioni genetiche associate al cancro del colon-retto.

È importante discutere con un medico quale metodo di screening è più adatto in base all'età, alla storia familiare, ai fattori di rischio e alle preferenze individuali.

OUTPUT (SESSION 2): Lo screening del tumore al colon si riferisce a una serie di test e procedure mediche che vengono eseguite per rilevare precocemente il cancro del colon-retto in individui asintomatici. Lo scopo principale è identificare sia i polipi precancerosi, che possono evolvere in tumore, sia i tumori nelle loro fasi iniziali, quando sono più facilmente trattabili e curabili. Le metodologie comuni per lo screening includono:

1. Colonscopia: un esame visivo diretto del colon mediante un tubo flessibile dotato di una telecamera, che permette di individuare e rimuovere i polipi.
2. Sigmoidoscopia flessibile: simile alla colonscopia, ma si concentra sull'esame della parte inferiore del colon.
3. Test del sangue occulto fecale (FOBT): verifica la presenza di sangue non visibile nelle feci, che può essere un indicatore di polipi o tumore.
4. Test del DNA fecale: analizza le feci per rilevare modifiche genetiche associate al cancro del colon.
5. Colonografia CT (colonscopia virtuale): utilizza scansioni TC per produrre immagini del colon e del retto.

La raccomandazione è che gli adulti inizino lo screening di routine tra i 45 e i 50 anni, a seconda delle linee guida nazionali e dei fattori di rischio individuali, come la storia familiare di cancro del colon. Parlare con un medico è fondamentale per determinare il piano di screening più adeguato.

Text similarity: 90%

3) **INPUT: In cosa consiste lo screening del cancro del colon?**

OUTPUT (SESSION 1): Lo screening del cancro del colon consiste in una serie di test mirati a identificare precocemente il cancro del colon-retto o polipi precancerosi nelle persone asintomatiche. Lo scopo principale è quello di rilevare la malattia in una fase iniziale, aumentando così le possibilità di trattamento efficace e successo. I principali metodi di screening includono:

1. Colonscopia: Una procedura in cui un medico utilizza un endoscopio per esaminare l'intero colon e il retto. Durante l'esame, è possibile rimuovere polipi o prelevare campioni di tessuto per ulteriori analisi.
2. Colonscopia virtuale (colonografia TC): Un esame non invasivo che utilizza la tomografia computerizzata per ottenere immagini dettagliate del colon e del retto.
3. Sigmoidoscopia flessibile: Questa procedura è simile alla colonscopia ma esamina solo l'ultima parte del colon e del retto.
4. Test del sangue occulto nelle feci (FOBT) e test immunochimico fecale (FIT): Test che verificano la presenza di sangue nelle feci, un potenziale indicatore di polipi o cancro.
5. Test di DNA fecale: Analizza il DNA presente nelle cellule di sfaldamento del colon nelle feci per individuare cambiamenti genetici che potrebbero indicare la presenza di tumori o polipi.

Il tipo di test di screening scelto dipende da diversi fattori, tra cui l'età del paziente, la storia familiare, i fattori di rischio personali e le preferenze individuali. È sempre consigliabile discutere con il proprio medico per determinare il percorso di screening più appropriato.

OUTPUT (SESSION 2): Lo screening del cancro del colon consiste in una serie di test progettati per rilevare precocemente il cancro del colon-retto e i polipi precancerosi. Questi test sono eseguiti in persone che non hanno sintomi del cancro, con l'obiettivo di identificare la malattia quando è nelle sue fasi iniziali e più trattabili. I principali metodi di screening includono:

1. Colonscopia: Una procedura in cui un medico esamina l'interno del colon usando un tubo flessibile con una telecamera. Durante la colonscopia, possono essere rimossi polipi o effettuate biopsie.
2. Sigmoidoscopia flessibile: Un test simile alla colonscopia, ma che esamina solo la parte inferiore del colon e del retto.
3. Ricerca di sangue occulto nelle feci (FOBT): Un test che verifica la presenza di sangue non visibile nelle feci, che potrebbe indicare la presenza di polipi o tumori.
4. Test del DNA fecale: Un esame che rileva mutazioni genetiche specifiche che possono essere segno della presenza di cancro del colon.
5. Colonografia CT (colonscopia virtuale): Un esame di imaging che utilizza una TAC per produrre immagini dettagliate del colon e del retto.

La scelta del test dipende da vari fattori, inclusi l'età, la storia familiare di cancro e altri fattori di rischio. In generale, si raccomanda agli adulti di iniziare lo screening tra i 45 e i 50 anni, a meno che ci siano motivi per iniziare prima. Consultare un medico può aiutare a determinare il miglior piano di screening personalizzato.

Text similarity: 87%

4) INPUT: Come si svolge lo screening del cancro del colon?

OUTPUT (SESSION 1): Lo screening del cancro del colon può essere effettuato attraverso diversi test, ognuno con il proprio metodo di svolgimento. Ecco come funzionano i più comuni:

1. Colonscopia:
   - Preparazione: Richiede una preparazione intestinale che consiste nel seguire una dieta specifica e nell'assumere lassativi per pulire il colon.
   - Procedura: Un medico inserisce un colonscopio (un tubo flessibile con una telecamera) attraverso il retto per esaminare l'intero colon. Durante l'esame, eventuali polipi possono essere rimossi e inviati in laboratorio per l'analisi.
2. Colonscopia virtuale (colonografia TC):
   - Preparazione: Simile alla colonscopia, con una pulizia intestinale.
   - Procedura: Utilizza una tomografia computerizzata per acquisire immagini dell'intestino crasso. Non richiede sedazione, ma può essere meno efficace nel rilevare polipi piccoli.
3. Sigmoidoscopia flessibile:
   - Preparazione: Di solito è necessaria una pulizia del retto e della parte inferiore del colon.
   - Procedura: Simile alla colonscopia ma esamina solo il retto e il colon sigmoideo.
4. Test del sangue occulto nelle feci (FOBT) e test immunochimico fecale (FIT):
   - Preparazione: Spesso non richiede restrizioni dietetiche o la sospensione di medicine, ma è importante seguire le istruzioni del test.
   - Procedura: Il paziente fornisce un campione di feci che viene analizzato per la presenza di tracce di sangue non visibili a occhio nudo.
5. Test di DNA fecale:
   - Preparazione: Non richiede una preparazione intestinale specifica.
   - Procedura: Il paziente raccoglie un campione di feci a casa e lo invia a un laboratorio, dove viene analizzato per identificare mutazioni genetiche specifiche e la presenza di sangue.

La scelta del test dipende da vari fattori individuali, tra cui età, rischio personale e preferenze, e dovrebbe essere discussa con il proprio medico.

OUTPUT (SESSION 2): Lo screening del cancro del colon viene svolto attraverso una serie di test che permettono di individuare precocemente segni di tumore o polipi precancerosi nel colon e nel retto. Ecco una descrizione di come si svolgono i principali test di screening:

1. Colonscopia:
   - Preparazione: Nei giorni precedenti, può essere richiesta una dieta speciale e l'assunzione di lassativi per pulire completamente l'intestino.
   - Procedura: Durante l'esame, un medico introduce un colonscopio (un tubo flessibile con una telecamera) nell'ano e lo guida attraverso il colon. Questo consente di visualizzare l'interno del colon e rilevare eventuali polipi o anomalie. Se necessario, il medico può rimuovere i polipi o prendere campioni di tessuto per una biopsia.
2. Sigmoidoscopia flessibile:
   - Preparazione: Simile alla colonscopia, ma di solito meno intensa.
   - Procedura: Si utilizza uno strumento simile a quello della colonscopia, ma la portata è limitata alla parte inferiore del colon. Questo esame è più rapido e meno invasivo.
3. Ricerca di sangue occulto nelle feci (FOBT):
   - Preparazione: Richiede l'adozione di una dieta specifica alcuni giorni prima del test per evitare falsi risultati.

- Procedura: Il paziente raccoglie campioni di feci a casa, che vengono poi inviati a un laboratorio per verificare la presenza di sangue non visibile all'occhio nudo.

4. Test del DNA fecale:
    - Procedura: Simile alla FOBT, si prelevano campioni di feci a casa. Il laboratorio analizza le feci per individuare mutazioni genetiche o marcatori di cancro del colon.
5. Colonografia CT (colonscopia virtuale):
    - Preparazione: Simile alla colonscopia tradizionale, il colon deve essere pulito.
    - Procedura: Durante la scansione TC, il paziente giace su un lettino mentre una macchina a raggi X scatta immagini del colon. Questo test è meno invasivo, ma se riscontra anomalie, potrebbe essere necessaria una colonscopia tradizionale.

Ogni test ha i suoi vantaggi e limitazioni, e la scelta del metodo più adatto dipende dalle condizioni individuali del paziente, dalla disponibilità dei test, e dalle raccomandazioni del medico.

Text similarity: 88%

5) INPUT: Come viene eseguito lo screening del cancro del colon?

OUTPUT (SESSION 1): Lo screening del cancro del colon può essere eseguito tramite vari test, ciascuno con un procedimento specifico. Ecco una panoramica dei principali metodi:

1. Colonscopia:
    - Preparazione: I pazienti devono seguire una dieta specifica e assumere lassativi il giorno prima per pulire l'intestino.
    - Procedura: Un medico inserisce un colonscopio, che è un tubo flessibile con una telecamera, attraverso il retto fino al colon, per visualizzare l'intero tratto intestinale. Eventuali polipi o campioni di tessuto possono essere rimossi o prelevati per ulteriori esami.
2. Colonscopia virtuale (colonografia TC):
    - Preparazione: Simile a quella della colonscopia con una pulizia completa del colon.
    - Procedura: Viene eseguita una scansione TC dopo l'insufflazione di aria o CO2 nel colon per ottenere immagini dettagliate. Non richiede sedazione e permette una visualizzazione dettagliata del colon.
3. Sigmoidoscopia flessibile:
    - Preparazione: Di solito richiede una pulizia parziale del colon inferiore.
    - Procedura: Un sigmoidoscopio, simile a un colonscopio ma più corto, viene inserito nel retto per esaminare il retto e il colon sigmoideo.
4. Test del sangue occulto nelle feci (FOBT) e test immunochimico fecale (FIT):
    - Preparazione: In genere non richiede preparazioni specifiche.
    - Procedura: Il paziente raccoglie un piccolo campione di feci a casa, che viene poi testato in laboratorio per verificare la presenza di sangue occulto, che può indicare la presenza di polipi o cancro.
5. Test di DNA fecale:
    - Preparazione: Non richiede una preparazione intestinale particolare.
    - Procedura: Il paziente raccoglie un campione di feci a casa. Questo viene inviato a un laboratorio, dove viene analizzato per cercare alterazioni genetiche specifiche associate al cancro del colon-retto.

Questi test hanno lo scopo di rilevare precocemente il cancro del colon-retto o di identificarne i precursori, aumentando così le possibilità di un trattamento efficace. La scelta del test più adeguato deve essere fatta in collaborazione con un medico, in base ai rischi individuali e alle preferenze del paziente.

OUTPUT (SESSION 2): Lo screening del cancro del colon viene eseguito utilizzando una serie di test progettati per rilevare polipi precancerosi e tumori nel colon e nel retto prima che causino sintomi. Ecco come si eseguono i principali test di screening:

1. Colonscopia:
    - Preparazione: Nei giorni precedenti l'esame, si richiede una dieta specifica e l'assunzione di lassativi per pulire completamente l'intestino.
    - Procedura: Un colonscopio (un tubo flessibile con una telecamera) viene inserito nell'ano e guidato attraverso il colon per visualizzare l'interno e rilevare eventuali anomalie. Durante la procedura, il medico può rimuovere polipi o prelevare campioni per una biopsia.
2. Sigmoidoscopia flessibile:
    - Preparazione: Richiede una pulizia meno intensa rispetto alla colonscopia completa.
    - Procedura: Un tubo simile viene inserito per esaminare solo la parte inferiore del colon. Questo esame è solitamente più veloce e può essere fatto senza sedazione.
3. Ricerca di sangue occulto nelle feci (FOBT):
    - Preparazione: Si potrebbero seguire indicazioni dietetiche specifiche per alcuni giorni.
    - Procedura: I pazienti raccolgono campioni di feci a casa, che vengono poi testati in laboratorio per rilevare sangue non visibile.
4. Test del DNA fecale:
    - Procedura: Campioni di feci vengono raccolti a casa e analizzati in laboratorio per mutazioni genetiche legate al cancro del colon.
5. Colonografia CT (colonscopia virtuale):
    - Preparazione: Simile alla colonscopia tradizionale, richiede la pulizia del colon.
    - Procedura: Il paziente si sdraia su un lettino in un macchinario che esegue scansioni TC per captare immagini dettagliate del colon. Se vengono trovati polipi o altre anomalie, potrebbe essere richiesto un ulteriore esame mediante colonscopia tradizionale.

Ogni test ha i suoi pro e contro, e la scelta di quale eseguire dipende da vari fattori tra cui età, storia familiare e condizioni mediche personali. Un medico può aiutare a determinare il test di screening più adatto per te.

Text similarity: 86%

6) **INPUT: A cosa serve lo screening del carcinoma del colon?**

OUTPUT (SESSION 1): Lo screening del carcinoma del colon ha l'obiettivo principale di rilevare la malattia in stadi precoci, quando è più trattabile e le possibilità di cura sono maggiori. Ecco a che cosa serve nello specifico:

1. Individuazione precoce: Rilevare il cancro del colon-retto in fase iniziale, prima che si manifestino sintomi evidenti. Questo consente interventi tempestivi, migliorando le possibilità di trattamento efficace e riducendo il rischio di mortalità.

2. Prevenzione: Identificare e rimuovere i polipi precancerosi (adenomi), che sono escrescenze nel colon che potrebbero evolvere in cancro se non trattate. La loro rimozione aiuta a prevenire lo sviluppo del cancro.
3. Riduzione della mortalità: Statistiche e studi hanno dimostrato che lo screening regolare riduce il tasso di mortalità associato al cancro del colon-retto, grazie alla possibilità di trattare la malattia prima che diventi avanzata.
4. Guida al trattamento: I risultati dello screening possono aiutare a determinare il miglior corso d'azione terapeutico, che potrebbe includere chirurgia, chemioterapia, o altre forme di trattamento.

   In sintesi, lo screening è un'importante misura preventiva che contribuisce a migliorare la salute e la longevità della popolazione, permettendo di affrontare il carcinoma del colon con maggiore successo.

OUTPUT (SESSION 2): Lo screening del carcinoma del colon serve a rilevare precocemente la presenza di tumori o polipi precancerosi nel colon e nel retto. L'obiettivo principale dello screening è individuare questi polipi o tumori in una fase iniziale, quando sono solitamente più trattabili e, quindi, aumentare le possibilità di cura e sopravvivenza.

I benefici principali dello screening includono:

1. Prevenzione: Identificare e rimuovere polipi precancerosi può prevenire lo sviluppo di un tumore maligno.
2. Diagnosi precoce: Rilevare il cancro del colon-retto nelle fasi iniziali consente trattamenti più efficaci e meno invasivi, aumentando significativamente le probabilità di guarigione.
3. Riduzione della mortalità: Lo screening regolare ha dimostrato di ridurre il tasso di mortalità associato al carcinoma del colon, poiché le cure sono più efficaci nei primi stadi della malattia.
4. Consapevolezza dei fattori di rischio: Può anche aiutare a identificare individui con una predisposizione genetica o altri fattori di rischio, che necessitano di monitoraggio e controllo più frequenti.

   Lo screening del colon-retto è generalmente raccomandato per gli adulti a partire dai 45-50 anni, o prima se ci sono fattori di rischio elevati, come una storia familiare di cancro del colon. Parlare con un medico aiuta a determinare il miglior piano di screening e le tempistiche più appropriate per la propria situazione personale.

Text similarity: 90%

7) **INPUT: Perché si fa lo screening del carcinoma del colon?**

OUTPUT (SESSION 1): Lo screening del carcinoma del colon viene effettuato per diverse ragioni fondamentali:

1. Individuazione precoce: Permette di rilevare il cancro del colon-retto in stadi iniziali, quando potrebbe non esserci la presenza di sintomi. Un'identificazione precoce facilita trattamenti più efficaci e aumenta le probabilità di guarigione.
2. Prevenzione: Lo screening consente di identificare e rimuovere polipi precancerosi (piccole escrescenze nel colon), riducendo significativamente il rischio che questi si trasformino in tumori maligni.
3. Riduzione della mortalità: Attraverso la diagnosi precoce e il trattamento tempestivo, lo screening regolare aiuta a ridurre il tasso di mortalità associato al cancro del colon-retto. Numerosi studi dimostrano che lo screening abbassa la probabilità di morte provocata da questo tipo di cancro.

4. Intervento tempestivo: Identificare il cancro prima che si diffonda permette di scegliere il trattamento più indicato, che può includere chirurgia, chemioterapia o altre terapie mirate.
5. Consapevolezza della salute: Partecipare a programmi di screening regolari incoraggia le persone a mantenere un atteggiamento proattivo riguardo alla loro salute, portandole a fare controlli medici regolari e a discutere eventuali fattori di rischio con i loro medici.

   In sintesi, lo screening del carcinoma del colon è un'importante strategia di salute pubblica progettata per prevenire la malattia e salvare vite.

OUTPUT (SESSION 2): Lo screening del carcinoma del colon si effettua per diversi motivi fondamentali:

1. Prevenzione: Consente di individuare e rimuovere i polipi precancerosi prima che possano evolvere in tumori maligni, riducendo così il rischio di sviluppare un carcinoma del colon-retto.
2. Diagnosi precoce: Rileva i tumori nelle loro fasi iniziali, quando sono più facilmente trattabili e le possibilità di successo terapeutico sono maggiori.
3. Riduzione della mortalità: Grazie alla diagnosi precoce e al trattamento tempestivo, lo screening contribuisce a ridurre il tasso di mortalità associato al carcinoma del colon-retto.
4. Miglioramento delle opzioni di trattamento: Il trattamento dei tumori scoperti precocemente tende ad essere meno invasivo e più efficace rispetto a quelli scoperti in uno stadio avanzato.
5. Identificazione dei fattori di rischio: Lo screening può rilevare individui con una maggiore predisposizione genetica o altri fattori di rischio, permettendo una sorveglianza più attenta e interventi preventivi tempestivi.

   In sintesi, lo screening del carcinoma del colon è una misura preventiva importante che può salvare vite, migliorare la qualità della vita e ridurre i costi associati al trattamento di tumori in fase avanzata. È generalmente raccomandato per gli adulti a partire dai 45-50 anni, o prima se ci sono fattori di rischio particolari.

Text similarity: 89%

8) **INPUT: Perché è consigliabile fare lo screening del carcinoma del colon?**

OUTPUT (SESSION 1): Lo screening del carcinoma del colon è consigliabile per diverse ragioni chiave:

1. Identificazione precoce: Consente di rilevare il cancro del colon-retto nelle fasi iniziali, quando è più trattabile e le possibilità di cura sono più elevate. I tumori individuati precocemente possono spesso essere rimossi completamente con interventi chirurgici.
2. Prevenzione: Può individuare polipi precancerosi, che sono escrescenze benigne ma potenzialmente pericolose nel colon. Rimuoverli durante i test di screening può prevenire la trasformazione in tumori maligni.
3. Riduzione della mortalità: Lo screening regolare è stato dimostrato efficace nel ridurre i tassi di mortalità legati al cancro del colon-retto grazie alla diagnosi e al trattamento tempestivi.
4. Diminuzione di trattamenti invasivi: Individuare il cancro in uno stadio precoce può spesso limitare la necessità di trattamenti più invasivi o estesi, migliorando la qualità della vita e riducendo le complicazioni.

5. Consapevolezza dei fattori di rischio: Partecipare allo screening offre anche l'opportunità di discutere con il proprio medico i fattori di rischio personali, come la storia familiare e altri elementi correlati alla salute, che possono influenzare la frequenza e la tipologia di screening necessaria.
6. Incoraggiamento della salute generale: Fare screening regolarmente può incitare le persone a mantenere uno stile di vita sano e a essere consapevoli della loro salute, promuovendo misure preventive più ampie.
   In sintesi, lo screening del carcinoma del colon è uno strumento cruciale per la prevenzione e il controllo del cancro del colon-retto, offrendo un potenziale significativo di ridurre l'incidenza e la mortalità associata a questa malattia.

OUTPUT (SESSION 2): È consigliabile fare lo screening del carcinoma del colon per diverse ragioni chiave:

1. Prevenzione: Lo screening permette di identificare e rimuovere i polipi precancerosi prima che possano trasformarsi in tumori maligni, riducendo il rischio di sviluppare un carcinoma del colon-retto.
2. Diagnosi precoce: Identifica il cancro nelle sue fasi iniziali, quando è più facilmente trattabile e le probabilità di cura sono maggiori.
3. Riduzione della mortalità: Grazie alla diagnosi e al trattamento tempestivo, lo screening aiuta a ridurre significativamente il tasso di mortalità legato al carcinoma del colon.
4. Trattamenti meno invasivi: I tumori individuati precocemente possono essere trattati con procedure meno invasive e con un maggiore successo terapeutico.
5. Identificazione dei fattori di rischio: Lo screening può rivelare una predisposizione genetica o altri fattori di rischio, offrendo l'opportunità di una sorveglianza e di interventi preventivi adeguati.
   In sintesi, lo screening regolare è una misura preventiva che può salvare vite umane, migliorare la qualità di vita e ridurre i costi associati al trattamento del cancro in stadi avanzati. Gli esperti raccomandano solitamente di iniziare lo screening a partire dai 45-50 anni, ma in presenza di fattori di rischio particolari, potrebbe essere necessario iniziare prima. Consultare un medico consente di determinare il miglior approccio e programma di screening per la propria situazione personale.

Text similarity: 87%

9) **INPUT: Quale è l'utilità dello screening del cancro del colon?**

OUTPUT (SESSION 1): L'utilità dello screening del cancro del colon risiede principalmente nei seguenti aspetti:

1. Rilevamento Precoce: Permette di individuare il cancro del colon-retto nelle fasi iniziali, quando il trattamento è generalmente più efficace e le probabilità di guarigione sono più alte. Un tumore individuato precocemente può spesso essere rimosso chirurgicamente con successo.
2. Prevenzione: Lo screening può identificare polipi precancerosi nel colon, che possono essere rimossi prima che evolvano in tumori maligni. Ciò contribuisce a prevenire lo sviluppo del cancro del colon-retto.
3. Riduzione della Mortalità: Studi dimostrano che lo screening regolare riduce i tassi di mortalità associati al cancro del colon-retto, grazie al rilevamento precoce e all'intervento rapido.

4. Limitazione dei Trattamenti Invasivi: Rilevare il tumore in uno stadio iniziale può ridurre la necessità di trattamenti invasivi più complessi e prolungati, migliorando la qualità della vita per i pazienti.
5. Consapevolezza e Prevenzione: Lo screening incoraggia le persone a essere più consapevoli della loro salute colica e a discutere con i medici il loro rischio di cancro del colon-retto, favorendo uno stile di vita più salutare.
6. Informazioni sui Fattori di Rischio: Durante le visite di screening, i medici possono consigliare meglio i pazienti sui loro fattori di rischio, personalizzando le raccomandazioni in base alla storia familiare, all'età e ad altri aspetti di salute.

   In sintesi, lo screening del cancro del colon è uno strumento essenziale nella strategia di prevenzione e controllo del cancro del colon-retto, contribuendo a salvare vite e migliorare le esperienze di salute.

OUTPUT (SESSION 2): L'utilità dello screening del cancro del colon risiede in diversi aspetti fondamentali che migliorano la gestione della salute e salvano vite:

1. Prevenzione: Attraverso lo screening, è possibile identificare e rimuovere polipi precancerosi prima che si trasformino in tumori maligni, riducendo significativamente il rischio di sviluppare un cancro del colon.
2. Diagnosi precoce: Rilevare il cancro del colon nelle fasi iniziali consente un trattamento più efficace rispetto a quando la malattia è in uno stadio avanzato. La diagnosi precoce è associata a migliori tassi di sopravvivenza.
3. Riduzione della mortalità: Gli screening regolari hanno dimostrato di ridurre la mortalità associata al carcinoma del colon, grazie alla possibilità di trattamenti tempestivi e appropriati.
4. Interventi meno invasivi: Il trattamento dei tumori individuati in fase iniziale è spesso meno invasivo e più efficace, con un minore impatto sulla qualità della vita del paziente.
5. Identificazione dei fattori di rischio: Lo screening è utile per identificare individui con un rischio elevato, come quelli con una storia familiare di cancro del colon, che potrebbero beneficiare di un monitoraggio più frequente.

   Nel complesso, lo screening del colon-retto è un'importante strategia di salute pubblica che contribuisce a prevenire lo sviluppo del carcinoma del colon e a migliorare le opzioni di trattamento e la prognosi per chi lo sviluppa. È generalmente raccomandato per gli adulti a partire dai 45-50 anni, a meno che non ci siano specifici fattori di rischio che richiedono un approccio diverso.

Text similarity: 85%

10) INPUT: A chi è rivolto lo screening del cancro del colon?

OUTPUT (SESSION 1): Lo screening del cancro del colon è generalmente rivolto a:

1. Adulti di età superiore ai 45 anni: In molte linee guida, l'età consigliata per iniziare lo screening è 45 anni, a causa dell'aumento del rischio di cancro del colon-retto con l'età.
2. Persone con storia familiare di cancro del colon-retto o polipi: Gli individui con parenti di primo grado che hanno avuto il cancro del colon o polipi adenomatosi potrebbero dover iniziare lo screening prima dei 45 anni e con maggiore frequenza.
3. Individui con condizioni ereditarie: Quelli affetti da sindromi ereditarie, come la sindrome di Lynch o la poliposi adenomatosa familiare (FAP), che aumentano il rischio di cancro del colon, necessitano di monitoraggio più attento e screening anticipato.

4. Persone con malattie infiammatorie intestinali: Coloro che hanno malattie come la colite ulcerosa o il morbo di Crohn sono a rischio maggiore e potrebbero richiedere uno screening più frequente.
5. Individui che hanno avuto polipi o cancro del colon in passato: Questi pazienti necessitano di controlli regolari per prevenire la recidiva o individuare nuove crescite precoci.
6. Persone con altri fattori di rischio: Condizioni come l'obesità, una dieta povera di fibre e ricca di grassi, l'uso prolungato di alcol e tabacco possono aumentare il rischio, rendendo lo screening una considerazione importante.

   È fondamentale per ogni individuo discuterne con il proprio medico per determinare il momento e il tipo più appropriato di screening in base alla propria situazione personale e fattori di rischio.

OUTPUT (SESSION 2): Lo screening del cancro del colon è generalmente rivolto a:

1. Adulti sopra i 45-50 anni: Le linee guida raccomandano che gli individui inizino lo screening regolare per il cancro del colon a partire da questa fascia di età, poiché il rischio di sviluppare la malattia aumenta con l'avanzare degli anni.
2. Persone con storia familiare di cancro del colon: Chi ha parenti di primo grado (genitori, fratelli o figli) che hanno avuto il cancro del colon potrebbe iniziare lo screening prima dei 45 anni, su consiglio di un medico.
3. Individui con condizioni ereditarie: Coloro che hanno sindromi genetiche come la poliposi adenomatosa familiare (FAP) o la sindrome di Lynch (cancro colorettale ereditario non poliposico, HNPCC) sono considerati a rischio elevato e possono necessitare di uno screening precoce e frequente.
4. Pazienti con storia personale di polipi o cancro del colon: Le persone che hanno avuto polipi precancerosi o cancro del colon in passato devono sottoporsi a regolari controlli.
5. Individui con malattie infiammatorie intestinali: Malattie come la colite ulcerosa o il morbo di Crohn possono aumentare il rischio di cancro del colon, rendendo necessari controlli più frequenti.

   È importante che ogni individuo discuta con il proprio medico per determinare il momento giusto per iniziare e la frequenza dello screening, considerando i propri fattori di rischio personali e familiari.

Text similarity: 85%

SCENARIO 2

Vuoi partecipare allo screening per il cancro del colon ma non sai come fare. Fai la tua domanda per ricevere chiarimenti.

1) **INPUT: A chi devo rivolgermi per ulteriori informazioni sullo screening del tumore del colon?**

OUTPUT (SESSION 1): Per ottenere ulteriori informazioni sullo screening del tumore del colon, puoi rivolgerti a:

1. Il tuo medico di base: È spesso il primo punto di contatto per discutere delle tue preoccupazioni e stabilire se lo screening è appropriato per te in base alla tua storia clinica e ai fattori di rischio.
2. Un gastroenterologo: Specialista in malattie del tratto gastrointestinale, può fornire dettagli specifici sui diversi tipi di screening disponibili e su quale potrebbe essere più indicato per te.
3. Centri di prevenzione oncologica: Strutture specializzate nella prevenzione e diagnosi precoce dei tumori possono offrire consulenze e programmi di screening specifici.
4. Cliniche o ospedali locali: Molti ospedali offrono programmi di screening del cancro del colon e hanno personale disponibile per rispondere alle domande.
5. Servizi di sanità pubblica: Possono fornire risorse educative e informazioni sui programmi di screening disponibili nella tua area.
6. Associazioni oncologiche: Organizzazioni senza scopo di lucro, come la Lega Italiana per la Lotta contro i Tumori (LILT), offrono informazioni e supporto per la prevenzione del cancro.

Consultare un professionista della salute competente ti aiuterà a comprendere meglio le opzioni di screening disponibili e a prendere decisioni informate sulla tua salute.

OUTPUT (SESSION 2): Per ottenere ulteriori informazioni sullo screening del tumore del colon, puoi rivolgerti a diverse fonti affidabili:

1. Medico di base: Il tuo primo riferimento dovrebbe essere il tuo medico di famiglia o il medico di base, che può fornire consigli personalizzati sulla base della tua storia medica e familiare.
2. Specialista in gastroenterologia: Un gastroenterologo è un medico specializzato nei disturbi del tratto gastrointestinale e può offrire dettagli specifici sugli screening e sui test più appropriati per te.
3. Centri di screening oncologico: Molti ospedali e cliniche offrono programmi di screening del cancro e dispongono di personale qualificato che può fornirti informazioni dettagliate.
4. Dipartimenti di sanità pubblica locali: Questi enti spesso dispongono di risorse e materiali informativi sui programmi di screening disponibili e sulle raccomandazioni correnti.
5. Associazioni oncologiche: Organizzazioni del settore, come l'Associazione Italiana per la Ricerca sul Cancro (AIRC) o simili, forniscono risorse educative e informazioni dettagliate sui benefici dello screening e sugli sviluppi della ricerca.

Consultando queste fonti, puoi ottenere una visione completa dello screening del tumore del colon e delle opzioni disponibili per te.

Text similarity: 83%

2) **Chi devo contattare per partecipare allo screening del cancro del colon?**

OUTPUT (SESSION 1): Per partecipare allo screening del cancro del colon, dovresti contattare:

1. Il tuo medico di base: Può valutare il tuo rischio, fornirti un riferimento per lo screening e darti informazioni su come procedere.
2. Un gastroenterologo: Questo specialista può offrirti una consulenza dettagliata sui test di screening disponibili e prenotare eventuali esami necessari, come una colonscopia.
3. Servizi di sanità pubblica locale: Molte regioni o stati hanno programmi di screening del cancro del colon che puoi contattare direttamente per informazioni e per prenotare un appuntamento.
4. Cliniche o ospedali locali: Queste strutture spesso offrono programmi di screening e possono aiutarti a organizzare gli esami necessari.

Assicurati di discutere con un professionista sanitario i tuoi specifici fattori di rischio e quale metodo di screening è più appropriato per te.

OUTPUT (SESSION 2): Per partecipare allo screening del cancro del colon, dovresti contattare:

1. Medico di base: Il tuo medico di famiglia è il primo passo per avviare il processo di screening. Può fornirti le informazioni necessarie e indirizzarti ai servizi di screening appropriati.
2. Programma di screening regionale: In molte regioni, esistono programmi di screening organizzati dal servizio sanitario pubblico. Contattare il dipartimento di sanità pubblica locale o visitare il sito web della tua azienda sanitaria locale può aiutarti a scoprire come partecipare.
3. Gastroenterologo: Se hai fattori di rischio particolari, potrebbe essere utile contattare uno specialista in gastroenterologia che può offrirti consigli più specifici e proporti test appropriati.

Rivolgendoti a uno di questi professionisti o servizi, potrai ottenere le informazioni necessarie per iscriverti al programma di screening più adatto alle tue esigenze.

Text similarity: 90%

3) **INPUT: Come si fa a partecipare allo screening del tumore del colon?**

OUTPUT (SESSION 1): Per partecipare allo screening del tumore del colon, puoi seguire questi passaggi:

1. Consulta il tuo medico di base: È il primo passo per discutere la tua idoneità allo screening. Il medico valuterà i tuoi fattori di rischio e ti fornirà raccomandazioni personalizzate, oltre a spiegarti i vari tipi di esami disponibili.
2. Ricevi una prescrizione o un referral: Il medico può fornirti una prescrizione per il test di screening appropriato o indirizzarti a uno specialista, come un gastroenterologo, per ulteriori esami.
3. Contatta un centro di screening o una clinica: Puoi prenotare un appuntamento presso un centro specializzato, un ospedale o una clinica che offre programmi di screening per il tumore del colon.
4. Prepara per il test: A seconda del tipo di screening raccomandato (ad esempio, colonscopia, test del sangue occulto nelle feci), potrebbe essere necessario seguire istruzioni specifiche, come una dieta particolare o l’assunzione di lassativi.

5. Effettua il test di screening: Segui le istruzioni fornite dal tuo medico o dal centro di screening per completare il test.
6. Ricevi i risultati e pianifica i passi successivi: Dopo il test, il tuo medico discuterà con te i risultati e qualsiasi ulteriore azione necessaria, che potrebbe includere follow-up o trattamenti aggiuntivi, se richiesto.
   Partecipare regolarmente allo screening, come indicato dal tuo medico, è importante per individuare precocemente eventuali problematiche e preservare la tua salute.

   OUTPUT (SESSION 2): Per partecipare allo screening del tumore del colon, segui questi passaggi:
1. Contatta il tuo medico di base: Inizia parlando con il tuo medico di famiglia. Possono consigliarti sui tempi appropriati per lo screening in base alla tua età, storia familiare e fattori di rischio personali.
2. Informazioni sui programmi di screening regionali: In molte regioni, il servizio sanitario pubblico offre programmi di screening organizzati per il cancro del colon. Puoi spesso trovare informazioni sui siti web delle aziende sanitarie locali o contattando il dipartimento di sanità pubblica locale per sapere come partecipare.
3. Ricevi l'invito: Nei programmi organizzati, potresti ricevere un invito diretto per partecipare allo screening. Assicurati di seguire le istruzioni fornite per prenotare il tuo appuntamento o per raccogliere i kit di test, se si tratta di uno screening basato su campioni fecali.
4. Esegui il test: Segui le istruzioni fornite per completare il test di screening, che può includere procedure come la ricerca di sangue occulto nelle feci o una colonscopia, a seconda del programma e dei tuoi fattori di rischio.
5. Ritira i risultati: Una volta completato lo screening, assicurati di ottenere i risultati e discuterli con il tuo medico per capire i prossimi passi, se necessari.
   Partecipare allo screening del tumore del colon è un importante passo preventivo per mantenere la tua salute e dovrebbe essere fatto regolarmente secondo le linee guida mediche appropriate.

   Text similarity: 86%

4) **INPUT: Come si fa ad accedere allo screening per il tumore del colon?**

   OUTPUT (SESSION 1): Accedere allo screening per il tumore del colon generalmente comporta i seguenti passaggi:
1. Consultazione con il Medico di Base: Parla con il tuo medico di base della tua età, della storia familiare e dei fattori di rischio personali per ricevere una raccomandazione sullo screening. Il medico può spiegarti i diversi tipi di screening disponibili e determinare quale sia più appropriato per te.
2. Piano di Screening: Basato sulla consulenza medica, il tuo medico ti guiderà sul piano più adatto, che potrebbe includere un referral per una colonscopia o per altri test come il test del sangue occulto nelle feci (FOBT) o un test immunochimico fecale (FIT).
3. Prenotare un Appuntamento: Una volta che hai il referral o la prescrizione, contatta una clinica, un ospedale o un centro di screening per prenotare il tuo test. Molte strutture ospedaliere pubbliche e private offrono questi servizi.
4. Preparazione per il Test: A seconda del tipo di screening, potrebbero esserti fornite istruzioni specifiche. Ad esempio, per una colonscopia, potrebbe essere necessario seguire una dieta speciale e assumere lassativi per pulire l'intestino.

5. Esame di Screening: Presentati all’appuntamento programmato per effettuare il test. Segui tutte le istruzioni del personale sanitario per assicurarti che l'esame sia eseguito correttamente.
6. Risultati e Follow-Up: Dopo il test, il tuo medico di base discuterà i risultati con te e ti consiglierà su eventuali passaggi successivi, che potrebbero includere ulteriore monitoraggio o trattamenti, se necessario.

   Partecipare regolarmente allo screening aiuta a prevenire il cancro del colon-retto rilevando eventuali anomalie nelle fasi iniziali.

   OUTPUT (SESSION 2): Per accedere allo screening per il tumore del colon, segui questi passaggi:

1. Consulta il tuo medico di base: Inizia parlando con il tuo medico di famiglia, che può valutare i tuoi fattori di rischio personali e familiari e darti indicazioni sui tempi e le modalità dello screening più adatto per te.
2. Informati sui programmi regionali di screening: Molte regioni offrono programmi di screening organizzati dal servizio sanitario pubblico. Puoi ottenere informazioni su questi programmi visitando il sito web della tua azienda sanitaria locale o contattando il dipartimento di sanità pubblica nella tua area.
3. Ricevi l'invito o contatta direttamente i servizi sanitari:
   - In alcune regioni, potresti ricevere un invito a partecipare al programma di screening quando raggiungi l'età raccomandata.
   - Se non ricevi un invito ma desideri partecipare, puoi contattare il servizio sanitario locale per chiedere di essere incluso nel programma.
4. Esegui il test: Una volta ottenuto l'accesso al programma, riceverai istruzioni dettagliate su come eseguire il test di screening, che potrebbe includere la raccolta di campioni fecali per la ricerca di sangue occulto o l'esecuzione di una colonscopia.
5. Segui le istruzioni e comunica i risultati: Dopo aver eseguito il test, invia i campioni al laboratorio designato, se richiesto, e attendi i risultati. Il tuo medico discuterà con te i risultati e i passi successivi, se necessario.

   Partecipando regolarmente allo screening del tumore del colon, puoi ridurre significativamente il rischio di sviluppare il cancro e migliorare le possibilità di successo del trattamento se viene rilevato precocemente.

   Text similarity: 84%

5) **INPUT: A chi mi devo rivolgere per partecipare allo screening del tumore del colon?**

   OUTPUT (SESSION 1): Per partecipare allo screening del tumore del colon, puoi rivolgerti a:

1. Medico di Base: È il primo contatto consigliato. Può discutere con te i tuoi fattori di rischio personali, spiegarti le varie opzioni di screening disponibili e fornirti una prescrizione o un indirizzo per eseguire i test necessari.
2. Gastroenterologo: Questo specialista può offrire consulenze dettagliate sui test di screening, come la colonscopia, e gestire direttamente l'esecuzione di questi esami.
3. Centro di Screening Locale: Molti ospedali e cliniche locali hanno programmi di screening del cancro del colon e possono assisterti nella prenotazione e nell'esecuzione degli esami appropriati.
4. Programmi di Sanità Pubblica: In alcune aree, esistono programmi di screening organizzati dalle autorità sanitarie locali che offrono test gratuiti o a costo ridotto, specialmente per le persone a rischio.

Contattare uno di questi professionisti o strutture ti aiuterà ad avviare il processo di screening e a curare la tua salute proattivamente.

OUTPUT (SESSION 2): Per partecipare allo screening del tumore del colon, dovresti rivolgerti a:

1. Medico di base: Il tuo medico di famiglia è un ottimo punto di partenza. Può fornirti informazioni sui programmi di screening disponibili e aiutarti a capire se sei idoneo a parteciparvi in base alla tua età, alla tua storia familiare e ad eventuali fattori di rischio.
2. Servizi sanitari locali: Contatta la tua azienda sanitaria locale o il dipartimento di sanità pubblica per ottenere informazioni sui programmi di screening organizzati nella tua area. Spesso questi programmi sono offerti dal servizio sanitario pubblico e sono destinati agli individui entro una certa fascia di età o con particolari fattori di rischio.
3. Centri di screening e ospedali: Molte strutture ospedaliere offrono servizi di screening del cancro del colon. Puoi contattare direttamente queste strutture per ottenere informazioni sui test disponibili, come la colonscopia o i test per la ricerca di sangue occulto nelle feci.

Chiedendo al tuo medico o contattando i servizi locali, potrai ricevere le informazioni necessarie per iscriverti al programma più adatto a te e assicurarti di seguire un piano di screening adeguato.

Text similarity: 92%

6) **INPUT: A quali persone è rivolto lo screening del cancro del colon?**

OUTPUT (SESSION 1): Lo screening del cancro del colon è rivolto principalmente a:

1. Adulti di età pari o superiore a 45 anni: Questo è l'inizio raccomandato per lo screening nella maggior parte delle linee guida, poiché il rischio di cancro del colon-retto aumenta con l'età.
2. Persone con storia familiare di cancro del colon-retto o polipi: Chi ha parenti di primo grado (come genitori, fratelli o figli) che hanno avuto la malattia potrebbe dover iniziare lo screening prima e con maggiore frequenza.
3. Individui con condizioni ereditarie: Coloro che presentano sindromi genetiche come la sindrome di Lynch o la poliposi adenomatosa familiare (FAP), che aumentano il rischio di cancro del colon, necessitano di uno screening anticipato e più frequente.
4. Persone con malattie infiammatorie intestinali: Le persone con condizioni come la colite ulcerosa o il morbo di Crohn hanno un rischio maggiore e potrebbero necessitare di uno screening più frequente.
5. Persone che hanno avuto polipi o cancro del colon in passato: Questi individui necessitano di controlli regolari per prevenire recidive o individuare nuove formazioni precoci.
6. Persone con altri fattori di rischio: Questi includono obesità, dieta povera, uso prolungato di alcol e tabacco, e stili di vita sedentari, che possono aumentare il rischio di cancro del colon.

È importante discutere con un medico quali criteri di screening si applicano al tuo caso specifico per garantire una diagnosi tempestiva e la migliore gestione possibile.

OUTPUT (SESSION 2): Lo screening del cancro del colon è rivolto principalmente alle seguenti persone:

1. Adulti di età pari o superiore a 45-50 anni: Le linee guida internazionali individuano questo gruppo di età come il punto di inizio per i normali programmi di screening, poiché il rischio di sviluppare il cancro del colon-retto aumenta con l'età.

2. Persone con una storia familiare di cancro del colon: Chi ha parenti di primo grado (genitori, fratelli o figli) che hanno avuto il cancro del colon dovrebbe iniziare lo screening prima dell'età raccomandata per la popolazione generale, spesso circa dieci anni prima rispetto all'età in cui il familiare è stato diagnosticato.
3. Individui con sindromi genetiche ereditarie: Quelli con condizioni come la poliposi adenomatosa familiare (FAP) o la sindrome di Lynch (cancro colorettale ereditario non poliposico) sono ad alto rischio e richiedono uno screening più precoce e frequente.
4. Pazienti con una storia personale di polipi o cancro del colon: Coloro che hanno già avuto polipi precancerosi o cancro del colon devono sottoporsi a controlli più regolari.
5. Individui con malattie infiammatorie intestinali: Chi soffre di condizioni come la colite ulcerosa o il morbo di Crohn, che aumentano il rischio di cancro del colon, dovrebbe effettuare screening più frequenti.

   Consultarsi con un medico è fondamentale per determinare il momento giusto e la frequenza per lo screening basato sui fattori di rischio personali.

   Text similarity: 88%

7) **INPUT: A che età si deve iniziare lo screening del tumore del colon?**

   OUTPUT (SESSION 1): L'età raccomandata per iniziare lo screening del tumore del colon è generalmente 45 anni per gli adulti a rischio medio. Questa raccomandazione è stata abbassata dai precedenti 50 anni a causa di un aumento dei casi di cancro del colon-retto tra le persone più giovani. Tuttavia, ci sono situazioni in cui potrebbe essere opportuno iniziare lo screening prima:

1. Storia familiare: Se hai un parente di primo grado che ha avuto il cancro del colon-retto o polipi adenomatosi, potresti dover iniziare lo screening prima, spesso intorno ai 40 anni, o a un'età che è di 10 anni inferiore a quella alla quale è stata diagnosticata la malattia al tuo parente, a seconda di quale dei due eventi venga prima.
2. Condizioni ereditarie: Chi è portatore di sindromi ereditarie ad alto rischio, come la sindrome di Lynch o la poliposi adenomatosa familiare (FAP), può richiedere uno screening iniziato anche in adolescenza o poco dopo, in base alla raccomandazione del medico.
3. Malattie infiammatorie intestinali: Le persone con condizioni come la colite ulcerosa o il morbo di Crohn potrebbero necessitare di una sorveglianza più ravvicinata iniziando prima, rispetto all'età standard raccomandata per quelli a rischio medio.

   È sempre importante discutere il tuo caso specifico con un medico per determinare il momento più opportuno per iniziare lo screening.

   OUTPUT (SESSION 2): Lo screening del tumore del colon è generalmente raccomandato a partire dai 45 anni per le persone con un rischio medio. Questa età è stata abbassata rispetto alla precedente raccomandazione di 50 anni, a causa di un aumento del rischio osservato nei gruppi di età più giovani.

   Se hai fattori di rischio più elevati, come una storia familiare di cancro del colon, sindromi genetiche ereditarie o precedenti personali di polipi o malattie infiammatorie intestinali, potresti dover iniziare lo screening anche prima. In questi casi, consultare il proprio medico è importante per determinare un piano di screening appropriato alle proprie esigenze individuali.

   Text similarity: 85%

8) **INPUT: A partire da che età bisogna fare lo screening del cancro del colon?**

OUTPUT (SESSION 1): Lo screening per il cancro del colon è generalmente raccomandato a partire dai 45 anni per gli adulti a rischio medio. Questa linea guida è stata stabilita per migliorare la diagnosi precoce, data l'incidenza crescente del cancro del colon-retto tra i più giovani. Tuttavia, in alcuni casi, potrebbe essere necessario cominciare prima:

1. Storia Familiare: Se hai parenti di primo grado che hanno avuto cancro del colon-retto o polipi, potrebbe essere raccomandato iniziare lo screening prima, intorno ai 40 anni o 10 anni prima rispetto all'età in cui la malattia è stata diagnosticata al tuo parente, a seconda di quale dei due viene prima.
2. Sindrome di Lynch o FAP: Chi ha sindromi genetiche ereditarie che aumentano il rischio può necessitare di uno screening molto anticipato, potenzialmente già nella tarda adolescenza o nei primi 20 anni.
3. Condizioni di Rischio Elevato: Per chi soffre di malattie infiammatorie croniche intestinali, come colite ulcerosa o morbo di Crohn, il medico potrebbe consigliare di iniziare lo screening prima dei 45 anni.

È consigliabile discutere con il proprio medico per personalizzare il piano di screening in base ai propri fattori di rischio.

OUTPUT (SESSION 2): Lo screening del cancro del colon è generalmente raccomandato a partire dai 45 anni per le persone a rischio medio. Questa raccomandazione riflette le linee guida attuali, che sono state adattate per rispondere all'aumento dei casi di tumore del colon-retto osservato nei gruppi di età più giovani. Per coloro che presentano fattori di rischio elevati, come una storia familiare di cancro del colon, sindromi genetiche ereditarie (come la sindrome di Lynch), o condizioni come la colite ulcerosa o il morbo di Crohn, potrebbe essere necessario iniziare lo screening prima. È importante discutere con il proprio medico per personalizzare un piano di screening in base ai propri fattori di rischio e alla storia personale.

Text similarity: 82%

9) **INPUT: Ogni quanti anni va fatto lo screening del tumore del colon?**

OUTPUT (SESSION 1): La frequenza con cui va effettuato lo screening del tumore del colon dipende dal tipo di test utilizzato e dal rischio individuale del paziente. Ecco le raccomandazioni generali:

1. Colonscopia: Ogni 10 anni, se i risultati sono normali e non ci sono altri fattori di rischio.
2. Sigmoidoscopia flessibile: Ogni 5 anni, oppure ogni 10 anni se combinata con un test immunochimico fecale (FIT) annuale.
3. Colonscopia virtuale (colonografia TC): Ogni 5 anni.
4. Test del sangue occulto nelle feci (FOBT) o test immunochimico fecale (FIT): Annualmente.
5. Test di DNA fecale (come Cologuard): Ogni 3 anni.

Queste sono linee guida generali per individui a rischio medio. Coloro che hanno un rischio maggiore, come quelli con una storia familiare di cancro del colon-retto o condizioni ereditarie particolari, potrebbero aver bisogno di uno screening più frequente o di un inizio anticipato. È importante discutere con il proprio medico per personalizzare la frequenza dello screening in base alle proprie esigenze.

OUTPUT (SESSION 2): La frequenza dello screening del tumore del colon dipende dal tipo di test utilizzato e dai fattori di rischio personali. Ecco una guida generale:

1. Colonscopia: Se i risultati sono normali, generalmente è raccomandata ogni 10 anni per le persone a rischio medio.
2. Sigmoidoscopia flessibile: In assenza di anomalie, si consiglia di ripeterla ogni 5 anni, spesso in combinazione con il test del sangue occulto fecale (FOBT) ogni 3 anni.
3. Test del sangue occulto fecale (FOBT) o test immunochimico fecale (FIT): Questi test, che cercano sangue nelle feci, sono generalmente raccomandati ogni anno.
4. Test del DNA fecale: Questo test, che ricerca mutazioni genetiche nelle feci, può essere fatto ogni 3 anni se i risultati sono normali.

La frequenza può essere diversa per chi ha fattori di rischio elevati, e quindi è importante discutere un piano personalizzato di screening con il proprio medico.

Text similarity: 86%

10) **INPUT: Ogni quanto tempo va eseguito lo screening del tumore del colon?**

OUTPUT (SESSION 1): La frequenza dello screening del tumore del colon dipende dal tipo di test scelto e dai fattori di rischio individuali. Ecco le raccomandazioni generali per una persona a rischio medio:

1. Colonscopia: Ogni 10 anni, se i risultati sono normali.
2. Sigmoidoscopia flessibile: Ogni 5 anni, oppure combinata con un test immunochimico fecale (FIT) annuale.
3. Colonscopia virtuale (colonografia TC): Ogni 5 anni.
4. Test del sangue occulto nelle feci (FOBT) o Test immunochimico fecale (FIT): Ogni anno.
5. Test di DNA fecale (come Cologuard): Ogni 3 anni.

Questi intervalli sono basati su linee guida generali. Per le persone con un rischio più elevato, come coloro con una storia familiare di cancro del colon-retto o condizioni ereditarie specifiche, potrebbero essere necessari screening più frequenti o iniziare prima. È sempre consigliabile consultarsi con il proprio medico per determinare il programma di screening più appropriato per la propria situazione.

OUTPUT (SESSION 2): La frequenza con cui va eseguito lo screening del tumore del colon dipende dal tipo di test scelto e dai fattori di rischio individuali. Ecco una panoramica generale:

1. Colonscopia: Se i risultati sono normali, è solitamente consigliata ogni 10 anni per le persone a rischio medio.
2. Sigmoidoscopia flessibile: Generalmente raccomandata ogni 5 anni, spesso abbinata a un test annuale per il sangue occulto fecale (FOBT) o FIT.
3. Test del sangue occulto fecale (FOBT) o test immunochimico fecale (FIT): Consigliati annualmente se utilizzati come principale metodo di screening.
4. Test del DNA fecale: Di solito si raccomanda ogni 3 anni in assenza di anomalie.

Queste linee guida possono variare per persone con fattori di rischio elevati, come una storia personale o familiare di polipi o cancro del colon. In questi casi, è importante consultare il proprio medico per un programma di screening personalizzato.

Text similarity: 85%

SCENARIO 3
Hai ricevuto un risultato positivo al test del sangue occulto nelle feci ma non sei sicuro delle sue implicazioni e di quali altri test potresti aver bisogno. Fai la tua domanda per avere chiarimenti.

1) **INPUT: Il risultato positivo del test del sangue occulto nelle feci significa che ho un tumore?**

   OUTPUT (SESSION 1): Un risultato positivo del test del sangue occulto nelle feci (FOBT o FIT) non significa necessariamente che hai un tumore. Questo risultato indica che è stata rilevata la presenza di sangue nelle feci, ma il sangue può provenire da diverse fonti. Oltre al cancro del colon, altre cause possono includere:

- Polipi non cancerosi nel colon.
- Emorroidi.
- Ulcerazioni o infiammazioni intestinali, come nel caso del morbo di Crohn o della colite ulcerosa.
- Alterazioni dietetiche o l'assunzione di determinati farmaci.

   Se il test del sangue occulto nelle feci è positivo, il medico probabilmente ti consiglierà un'ulteriore valutazione, solitamente tramite una colonscopia, per determinare la causa del sanguinamento e verificare l'eventuale presenza di polipi o tumori. È importante seguire le raccomandazioni del tuo medico e non trarre conclusioni affrettate solo basandosi sul risultato positivo del test.

   OUTPUT (SESSION 2): Un risultato positivo del test del sangue occulto nelle feci non significa necessariamente che tu abbia un tumore. Questo test rileva la presenza di sangue nelle feci, che può risultare da diverse cause, tra cui:

- Polipi: Crescite benigne nel colon che possono sanguinare.
- Emorroidi: Possono causare sanguinamento.
- Infiammazioni o infezioni intestinali: Condizioni come la colite possono causare la presenza di sangue.
- Altre lesioni non cancerose: Ulcere o traumi possono portare a sanguinamento.

   Un risultato positivo indica la necessità di ulteriori indagini, di solito tramite una colonscopia, per determinare la causa del sanguinamento. È importante discutere i risultati del test con il tuo medico, che può consigliarti i passi successivi appropriati.

   Text similarity: 83%

2) **INPUT: Avere un risultato positivo al test del sangue occulto equivale ad avere un cancro al colon?**

   OUTPUT (SESSION 1): No, un risultato positivo al test del sangue occulto nelle feci non equivale necessariamente ad avere un cancro al colon. Un esito positivo indica la presenza di sangue nelle feci, che può derivare da varie cause, non solo dal cancro. Possibili fonti di sanguinamento includono:

- Polipi non cancerosi.
- Emorroidi.
- Ulcerazioni o infiammazioni dell'intestino, come nel caso di colite ulcerosa o morbo di Crohn.
- Assunzione di alcuni alimenti o farmaci che possono causare sanguinamento.

Un risultato positivo è un segnale per eseguire ulteriori accertamenti, di solito una colonscopia, al fine di identificare la causa del sanguinamento e valutare la presenza di eventuali polipi o tumori. È fondamentale seguire le indicazioni del medico per ottenere una diagnosi accurata e le cure adeguate.

OUTPUT (SESSION 2): No, avere un risultato positivo al test del sangue occulto nelle feci non equivale necessariamente ad avere un cancro al colon. Questo test rileva la presenza di sangue nelle feci, che può essere causato da varie condizioni, non tutte cancerose. Ad esempio:

- Polipi non cancerosi: Alcuni polipi possono causare sanguinamento.
- Emorroidi: Possono causare sangue nelle feci.
- Inflammationi o infezioni: Come quelle causate dalla colite o altre malattie infiammatorie intestinali.
- Ulcerazioni o altre lesioni: Possono portare a sanguinamento gastrointestinale.

Un risultato positivo indica che è necessario effettuare ulteriori indagini per identificare la causa del sanguinamento, solitamente tramite una colonscopia. È importante discutere i risultati con il tuo medico per capire quali sono i passi successivi più appropriati.

Text similarity: 89%

3) **INPUT: Dopo un risultato positivo al test del sangue occulto nelle feci devo eseguire altri esami?**

OUTPUT (SESSION 1): Sì, dopo un risultato positivo al test del sangue occulto nelle feci, è generalmente consigliato eseguire ulteriori esami per determinare la causa del sanguinamento. Il passo successivo più comune è una colonscopia.
La colonscopia è una procedura che permette al medico di visualizzare l'interno del colon e del retto utilizzando un tubo flessibile con una telecamera. Questo esame consente di:

- Identificare e rimuovere eventuali polipi che potrebbero essere precancerosi.
- Diagnosticare la presenza di infiammazioni o altre condizioni patologiche.
- Se necessario, effettuare biopsie di qualsiasi tessuto anomalo per un'analisi più dettagliata.

È importante seguire le raccomandazioni del tuo medico per eseguire la colonscopia o altri esami suggeriti, al fine di avere una diagnosi accurata e decidere il miglior corso di azione.

OUTPUT (SESSION 2): Sì, dopo un risultato positivo al test del sangue occulto nelle feci, è importante eseguire ulteriori esami per determinare la causa del sanguinamento. La procedura più comune che viene raccomandata è la colonscopia.
La colonscopia consente al medico di esaminare direttamente l'interno del colon e del retto utilizzando un tubo flessibile con una videocamera. Durante la procedura, il medico può:

- Identificare e rimuovere polipi, se presenti.
- Prelevare campioni di tessuto (biopsia) per ulteriori analisi di eventuali anomalie riscontrate.

Questo esame aiuterà a chiarire se la causa del sanguinamento è non cancerosa, come polipi o emorroidi, o se è dovuta a condizioni più serie, come il cancro del colon. Discussione e pianificazione con il tuo medico sono fondamentali per comprendere i risultati del test e stabilire i corretti passi successivi.

Text similarity: 87%

4) **INPUT: Il risultato positivo del test del sangue occulto nelle feci può rappresentare un errore?**

OUTPUT (SESSION 1): Sì, un risultato positivo al test del sangue occulto nelle feci può essere un falso positivo. Questo significa che il test ha rilevato la presenza di sangue, che tuttavia potrebbe non derivare da un problema serio come il cancro del colon. Ci sono diversi fattori che possono influenzare il risultato, tra cui:

- Dieta: Consumo di alcuni alimenti (come carne rossa, alcuni frutti e verdure) chi può interferire con alcuni tipi di test FOBT.
- Farmaci: L'uso di farmaci anti-infiammatori non steroidei (FANS), aspirina o anticoagulanti può causare sanguinamento gastrointestinale.
- Condizioni Benigne: Disturbi come le emorroidi o le ulcere gastrointestinali possono causare la presenza di sangue nelle feci.

A causa della possibilità di falsi positivi, un test positivo di sangue occulto nelle feci richiede generalmente un'ulteriore valutazione, spesso attraverso una colonscopia. Questo esame consente di determinare la causa esatta del sanguinamento e di escludere o confermare la presenza di condizioni più gravi.

OUTPUT (SESSION 2): Sì, un risultato positivo del test del sangue occulto nelle feci può rappresentare un falso positivo, che si verifica quando il test indica la presenza di sangue ma non c'è una condizione seria come un cancro. Ci sono diverse ragioni per cui si possono verificare falsi positivi:

1. Dieta: Alcuni alimenti, come carne rossa, alcune verdure (ad esempio, ravanelli, cavolfiori), o integratori di vitamina C e ferro, possono influenzare i risultati del test.
2. Farmaci: L'uso di farmaci come l'aspirina o altri anti-infiammatori non steroidei (FANS) può causare sanguinamento gastrointestinale, influenzando i risultati.
3. Condizioni non cancerose: Sanguinamento da emorroidi, ragadi anali, o malattie infiammatorie intestinali può portare a un risultato positivo.

A causa di queste potenziali influenze sui risultati, un test positivo è generalmente seguito da una colonscopia per esaminare direttamente il colon e determinare la causa esatta del sanguinamento. È fondamentale discutere i risultati con il tuo medico per valutare i passi successivi appropriati.

Text similarity: 86%

5) **INPUT: Dopo un risultato positivo al test del sangue occulto nelle feci devo eseguire un altro test del sangue occulto di conferma?**

OUTPUT (SESSION 1): Di solito, dopo un risultato positivo al test del sangue occulto nelle feci, il passo successivo non è eseguire un altro test di conferma, ma sottoporsi a una colonscopia. La colonscopia è considerata il metodo standard per ulteriori indagini, in quanto offre una visualizzazione diretta dell'interno del colon e del retto per identificare la fonte del sanguinamento.
Una colonscopia permette di:

- Identificare polipi, tumori o altre anomalie.
- Eseguire biopsie di tessuti sospetti.

- Rimuovere polipi potenzialmente precancerosi.

Ripetere il test del sangue occulto senza procedere con una colonscopia potrebbe ritardare la diagnosi di eventuali condizioni che necessitano di attenzione medica. Pertanto, è importante seguire le istruzioni del tuo medico e pianificare una colonscopia per ottenere una valutazione accurata.

OUTPUT (SESSION 2): Dopo un risultato positivo al test del sangue occulto nelle feci, solitamente non si esegue un altro test del sangue occulto di conferma. Invece, il passo successivo raccomandato è di solito una colonscopia.
La colonscopia è importante perché permette di esaminare direttamente l'interno del colon e del retto per identificare la causa del sanguinamento. Attraverso questa procedura, è possibile non solo diagnosticare la presenza di polipi o altre anomalie, ma anche intervenire immediatamente, ad esempio rimuovendo polipi che potrebbero essere precancerosi.
Eseguire un altro test del sangue occulto, invece, potrebbe non fornire informazioni aggiuntive utili e ritardare l'opportunità di ottenere una diagnosi accurata. È essenziale seguire le indicazioni del tuo medico riguardo ai passi successivi dopo un risultato positivo.

Text similarity: 85%

6) **INPUT: Il risultato positivo del test del sangue occulto fecale può derivare dalle emorroidi?**

OUTPUT (SESSION 1): Sì, un risultato positivo al test del sangue occulto fecale può derivare dalla presenza di emorroidi. Le emorroidi sono considerati una causa benigna di sanguinamento rettale e possono portare a tracce di sangue nelle feci, che il test può rilevare. Tuttavia, poiché il sangue occulto nelle feci può anche indicare altre condizioni più serie come polipi o tumori nel colon o nel retto, è importante non trarre conclusioni affrettate solo basandosi su questo esito.
Un risultato positivo richiede ulteriori accertamenti, spesso attraverso una colonscopia, per determinare la causa esatta del sanguinamento e assicurarsi che non vi siano condizioni più gravi non diagnosticate. Seguire le indicazioni del tuo medico è fondamentale per ricevere una diagnosi accurata e il trattamento adeguato.

OUTPUT (SESSION 2): Sì, un risultato positivo del test del sangue occulto fecale può essere causato dalla presenza di emorroidi. Le emorroidi sono dilatazioni delle vene nella regione anale e rettale che possono causare sanguinamento. Questo sanguinamento può contaminare le feci e portare a un risultato positivo nel test del sangue occulto.
Tuttavia, un risultato positivo potrebbe anche significare la presenza di altre condizioni, alcune delle quali potrebbero essere più serie, come polipi precancerosi o cancro del colon. Per questo motivo, un risultato positivo viene generalmente seguito da ulteriori indagini, come una colonscopia, per determinare la causa esatta del sanguinamento.
È importante discutere i risultati del test con il tuo medico per comprendere il significato specifico nel tuo caso e pianificare gli ulteriori esami diagnostici necessari.

Text similarity: 89%

7) **INPUT: Le emorroidi possono giustificare un test del sangue occulto nelle feci positivo?**

OUTPUT (SESSION 1): Sì, le emorroidi possono giustificare un risultato positivo al test del sangue occulto nelle feci. Le emorroidi sono vene gonfie nel retto o nell'ano che possono sanguinare, soprattutto durante o dopo l'evacuazione, e questo sanguinamento può essere rilevato dal test.
Tuttavia, poiché un risultato positivo al test del sangue occulto può anche indicare altre condizioni più serie, come polipi o cancro del colon-retto, è importante che un risultato positivo venga seguito da ulteriori esami. Solitamente, il passo successivo è una colonscopia, che consente di esaminare direttamente l'interno del colon e del retto per determinare la causa esatta del sanguinamento.
È importante seguire le raccomandazioni mediche per garantire una diagnosi accurata e affrontare efficacemente qualsiasi problema di salute sottostante.

OUTPUT (SESSION 2): Sì, le emorroidi possono giustificare un test del sangue occulto nelle feci positivo. Le emorroidi sono vene gonfie nella regione anale e rettale che possono rompere e causare sanguinamento, il quale può essere rilevato dal test del sangue occulto nelle feci.
Tuttavia, poiché un test positivo può anche indicare altre condizioni, come polipi o tumore del colon, è importante eseguire ulteriori accertamenti per determinare la causa esatta del sanguinamento. Solitamente, il passo successivo consigliato è una colonscopia, che permette di visualizzare direttamente l'interno del colon e del retto per arrivare a una diagnosi precisa.
Discutere i risultati con il tuo medico è fondamentale per pianificare la gestione e gli esami successivi appropriati.

Text similarity: 84%

8) **INPUT: Cosa vuol dire risultare positivi al test del sangue occulto nelle feci?**

OUTPUT (SESSION 1): Risultare positivi al test del sangue occulto nelle feci significa che è stata rilevata la presenza di sangue nelle feci tramite l'esame. Questo sangue non è visibile a occhio nudo, ma il test è in grado di identificarlo. Un risultato positivo non indica necessariamente la presenza di un cancro, ma segnala che c'è del sanguinamento nel tratto gastrointestinale inferiore che necessita di ulteriori indagini.
Le possibili cause di sanguinamento includono:

- Polipi del colon: Crescite non cancerose che possono sanguinare.
- Cancro del colon-retto: Una delle possibili cause più serie.
- Emorroidi: Varici rettali che possono causare sanguinamento.
- Ulcere o infiammazioni: Condizioni come la colite ulcerosa o il morbo di Crohn.
- Diverticolosi: Piccole protrusioni nei tessuti del colon che possono sanguinare.

A seguito di un risultato positivo, è comune che un medico raccomandi una colonscopia per esaminare più accuratamente l'interno del colon e del retto, determinare la fonte del sanguinamento e prendere le misure appropriate. È importante seguire le indicazioni del medico per completare il processo diagnostico.

OUTPUT (SESSION 2): Risultare positivi al test del sangue occulto nelle feci significa che è stata rilevata la presenza di sangue nelle feci. Questo test è progettato per identificare tracce di sangue che non sono visibili a occhio nudo.

Un risultato positivo indica che potrebbe esserci sanguinamento nel tratto gastrointestinale, che può essere causato da una varietà di condizioni, tra cui:

- Polipi: Crescite che possono essere precancerose e che a volte sanguinano.
- Emorroidi: Vene gonfie nella zona rettale o anale che possono causare sanguinamento.
- Ulcere o infiammazioni: Condizioni come colite ulcerosa o altre infiammazioni intestinali.
- Tumori: Sia benigni che maligni, che possono sanguinare.

È importante notare che un risultato positivo non significa automaticamente la presenza di cancro. Tuttavia, è un segnale che richiede ulteriori indagini, tipicamente tramite una colonscopia, per determinare la causa specifica del sanguinamento. Consultare il proprio medico per discutere i risultati e i passi successivi è essenziale.

Text similarity: 82%

9) **INPUT: Sono risultato positivo al test del sangue occulto fecale, cosa devo fare?**

OUTPUT (SESSION 1): Se sei risultato positivo al test del sangue occulto fecale, il passo successivo più comunemente raccomandato è quello di sottoporsi a una colonscopia. La colonscopia è una procedura che consente di esaminare l'interno del colon e del retto per identificare la causa del sanguinamento. Ecco cosa dovresti fare:

1. Contatta il tuo medico: Informa il tuo medico del risultato del test. Lui o lei ti fornirà indicazioni su come procedere.
2. Programma una colonscopia: Il medico ti indirizzerà a un gastroenterologo per programmare una colonscopia. Questo esame è fondamentale per determinare se il sanguinamento è causato da polipi, cancro o altre condizioni come emorroidi o infiammazioni.
3. Preparati per l'esame: Segui le istruzioni fornite per la preparazione della colonscopia. Questa di solito include una dieta specifica e lassativi per pulire il colon.
4. Partecipa all'appuntamento: Assicurati di presentarti all'appuntamento per la colonscopia. L'esame aiuterà a ottenere una diagnosi accurata e a pianificare eventuali trattamenti necessari.
5. Ricevi i risultati e segui le raccomandazioni: Dopo la colonscopia, discuterai i risultati con il tuo medico per determinare i passi successivi, che possono includere trattamenti, cambiamenti dello stile di vita, o ulteriori monitoraggi.

Affrontare tempestivamente un risultato positivo e seguire le raccomandazioni del medico è importante per garantire una diagnosi accurata e un trattamento appropriato.

OUTPUT (SESSION 2): Se sei risultato positivo al test del sangue occulto fecale, il passo successivo raccomandato di solito è sottoporsi a una colonscopia. La colonscopia è un esame più dettagliato che consente di visualizzare l'interno del colon e del retto. Attraverso questo esame, il medico può:

- Identificare e rimuovere eventuali polipi presenti.
- Eseguire biopsie su tessuti sospetti per ulteriori esami.
- Escludere o confermare la presenza di condizioni serie, come il cancro del colon.

Ecco cosa dovresti fare dopo un risultato positivo:

1. Consulta il tuo medico di base: Discuti i risultati del test con il tuo medico per capire cosa significa specificamente per te e ricevere raccomandazioni su come procedere.

2. Programma una colonscopia: Il tuo medico ti indirizzerà probabilmente a un gastroenterologo per prenotare una colonscopia.
3. Prepara l'esame: Segui attentamente le istruzioni fornite per prepararti alla colonscopia, che potrebbero includere una dieta specifica e lassativi per pulire l'intestino.
4. Segui le raccomandazioni successive: In base ai risultati della colonscopia, il tuo medico discuterà i passi successivi, che potrebbero includere trattamenti o una pianificazione del monitoraggio futuro.

   I risultati positivi non significano necessariamente la presenza di un cancro, ma è cruciale fare ulteriori accertamenti per comprendere completamente la situazione.

   Text similarity: 85%

10) INPUT: Cosa fare in caso di esito positivo del test del sangue occulto nelle feci?

OUTPUT (SESSION 1): In caso di esito positivo del test del sangue occulto nelle feci, è importante intraprendere i seguenti passi per una valutazione più approfondita e accurata:

1. Contatta il tuo medico: Informa il tuo medico del risultato positivo. È essenziale discutere dell'esito del test per capire meglio cosa potrebbe significare nel tuo caso specifico.
2. Programma una colonscopia: Il medico probabilmente ti consiglierà di eseguire una colonscopia. Questa procedura è fondamentale per esaminare l'interno del colon e del retto per identificare la fonte del sanguinamento.
3. Segui le istruzioni per la preparazione: Prima della colonscopia, dovrai seguire istruzioni specifiche per preparare l'intestino, che possono includere una dieta speciale e l'uso di lassativi per pulire l'intestino.
4. Sottoponiti alla colonscopia: Questa procedura consentirà al medico di vedere se ci sono polipi, tumori o altre fonti di sanguinamento nel colon. Potrebbe anche consentire la rimozione di polipi o il prelievo di campioni di tessuto per ulteriori analisi.
5. Interpretazione dei risultati: Dopo la colonscopia, il tuo medico discuterà i risultati con te e ti consiglierà sui passi successivi, che potrebbero includere ulteriori monitoraggi, un intervento o cambiamenti nello stile di vita.

   Affrontare un risultato positivo rapidamente e seguire le indicazioni mediche è fondamentale per assicurarsi che, se presente, ogni potenziale problema possa essere trattato nel modo più efficace possibile.

OUTPUT (SESSION 2): In caso di un esito positivo del test del sangue occulto nelle feci, è importante seguire questi passi per approfondire la situazione e determinare la causa del sanguinamento:

1. Consulta il tuo medico: Parla con il tuo medico di base per discutere il risultato del test. Questo è un passo cruciale per comprendere il significato dell'esito positivo e per pianificare i successivi accertamenti.
2. Preparati per ulteriori esami: Il tuo medico probabilmente ti consiglierà di sottoporsi a una colonscopia. Questo esame è il metodo più efficace per esaminare l'interno del colon e del retto, identificare la fonte del sanguinamento, e rimuovere o biopsiare polipi o altre anomalie.
3. Programma la colonscopia: Fissa un appuntamento con un gastroenterologo per eseguire la colonscopia. Assicurati di seguire attentamente tutte le istruzioni di preparazione fornite dal tuo medico per garantire che l'intestino sia pulito e il test sia accurato.

4. Discussione dei risultati: Dopo la colonscopia, il medico discuterà con te i risultati e i passi successivi. Se vengono identificati polipi o altre condizioni, potrebbero essere necessari interventi o monitoraggi ulteriori.
   L'obiettivo di questi passi è identificare o escludere seri problemi di salute e assicurarsi che tu riceva le cure appropriate il prima possibile.

   Text similarity: 84%

SCENARIO 4

A seguito di un risultato positivo al test del sangue occulto nelle feci, ti è stato consigliato di sottoporti a una colonscopia e non sei sicuro della procedura e di come prepararti. Fai la tua domanda per avere informazioni dettagliate.

1) **INPUT: Come si svolge la colonscopia?**

OUTPUT (SESSION 1): La colonscopia è una procedura medica utilizzata per esaminare l'interno del colon e del retto. Ecco come si svolge generalmente la colonscopia:

1. Preparazione Intestinale: Prima dell'esame, è necessario pulire completamente l'intestino. Ciò di solito implica seguire una dieta speciale priva di fibre il giorno prima e assumere lassativi forti come il giorno precedente l'esame. Questa preparazione è fondamentale per consentire una visualizzazione chiara della mucosa intestinale.
2. Digiuno: Dovrai evitare di mangiare o bere qualsiasi cosa dopo la mezzanotte prima della procedura, salvo diverse indicazioni del medico.
3. Sedazione: Prima dell'inizio della colonscopia, ti verranno somministrati farmaci sedativi per aiutarti a rilassarti e ridurre il disagio. Questo significa che sarai molto sonnolento o addormentato durante la procedura, ma non completamente in anestesia generale.
4. Esecuzione dell'Esame:
    - Il medico utilizza un colonscopio, un tubo flessibile dotato di una telecamera e una luce, che viene inserito attraverso il retto e spinto delicatamente nel colon.
    - Il gas (di solito aria o CO_2) potrebbe essere introdotto nel colon per distenderlo e permettere una migliore visualizzazione della parete colica.
    - Durante la procedura, il medico cerca polipi, tumori o altre anomalie. Polipi piccoli possono essere rimossi durante l'esame, e campioni di tessuto possono essere prelevati per una biopsia se necessario.
5. Post-procedura: Dopo la colonscopia, sarai tenuto sotto osservazione finché gli effetti della sedazione non si saranno ridotti. Potresti avvertire gonfiore o crampi a causa dell'aria introdotta, ma questi sintomi di solito si risolvono rapidamente.
6. Risultati: Una volta che ti sei ripreso, il medico discuterà i risultati preliminari con te. Se sono stati prelevati tessuti per la biopsia, ci vorrà del tempo per ricevere i risultati di laboratorio.

   È importante ricordare che per la preparazione e il recupero potresti necessitare di assistenza, quindi è meglio pianificare in anticipo e avere qualcuno che ti accompagni a casa dopo l'esame.

OUTPUT (SESSION 2): La colonscopia è una procedura medica utilizzata per esaminare l'interno del colon e del retto. Ecco come si svolge generalmente:

1. Preparazione: Nei giorni precedenti la colonscopia, il paziente deve seguire una dieta specifica (spesso liquida) e assumere lassativi per pulire completamente l'intestino. È essenziale seguire attentamente queste istruzioni per garantire che il colon sia privo di residui alimentari che potrebbero ostacolare la visione durante l'esame.
2. Sedazione: Poco prima dell'inizio della procedura, viene di solito somministrata una forma di sedazione o anestesia leggera al paziente per ridurre il disagio e l'ansia. Il livello di sedazione può variare a seconda delle necessità individuali e delle prassi della struttura sanitaria.
3. Procedura: Un colonscopio, un tubo flessibile con una telecamera all'estremità, viene inserito delicatamente attraverso l'ano e guidato lungo il retto e il colon. Il medico può gonfiare il colon con aria o anidride carbonica per una migliore visualizzazione delle pareti intestinali.

4. Esame e interventi: Durante la colonscopia, il medico osserva l'immagine trasmessa dalla telecamera per individuare polipi, tumori o altre anomalie. Se vengono trovati polipi, spesso vengono rimossi durante la procedura. Campioni di tessuti (biopsie) possono essere prelevati per ulteriori analisi in laboratorio.
5. Conclusione e recupero: Dopo la procedura, il colonoscopio viene rimosso e il paziente viene monitorato mentre la sedazione svanisce. Potrebbe verificarsi una leggera sensazione di gonfiore o crampi causata dall'aria introdotta durante l'esame, ma dovrebbe diminuire rapidamente.
6. Risultati: Il medico discuterà i risultati della colonscopia. Se sono stati rimossi polipi o eseguite biopsie, i risultati potrebbero richiedere qualche giorno.
   La colonscopia è un esame sicuro e altamente efficace per la diagnosi precoce e la prevenzione del cancro del colon e altre condizioni gastrointestinali.

   Text similarity: 89%

2) **INPUT: In cosa consiste esattamente la colonscopia?**

   OUTPUT (SESSION 1): La colonscopia è una procedura diagnostica che consente di esaminare l'intero colon e il retto per individuare anomalie, polipi o malattie come il cancro. Ecco in cosa consiste esattamente:

1. Preparazione Intestinale: Prima dell'esame, è fondamentale pulire completamente l'intestino per garantire una buona visibilità. Questo di solito comporta:
   - Seguire una dieta a basso contenuto di fibre nei giorni precedenti l'esame.
   - Assumere lassativi forti il giorno prima della colonscopia per svuotare l'intestino.
   - Digiunare da solide e liquidi trasparenti almeno dalla mezzanotte prima del giorno dell'esame, seguendo le indicazioni del medico.
2. Sedazione: Prima dell'inizio della procedura, al paziente viene somministrata una sedazione per ridurre il disagio e l'ansia. Questo stato di semicoscienza permette di non avvertire dolore durante l'esame.
3. Esecuzione della colonscopia:
   - Un colonscopio, che è un tubo flessibile e sottile dotato di una telecamera e una luce, viene inserito delicatamente attraverso il retto.
   - Il colonscopio viene avanzato lentamente attraverso il colon, permettendo al medico di visualizzare l'intera mucosa intestinale sul monitor.
   - Viene spesso insufflata aria o anidride carbonica nel colon per distenderlo e migliorare la visibilità e l'accesso.
4. Valutazione e Interventi: Durante l'esame, il medico cerca eventuali polipi, tumori o altre anomalie. Se vengono individuati polipi o tessuti sospetti, possono essere:
   - Rimossi.
   - Biopsiati, ovvero prelevati per ulteriori analisi in laboratorio.
5. Post-procedura: Dopo l'esame, il paziente viene monitorato finché gli effetti della sedazione non svaniscono. Si possono avvertire gonfiore o crampi leggeri, ma questi sintomi di solito si risolvono rapidamente.
6. Discussione dei Risultati: Al termine della procedura, il medico discuterà i risultati preliminari. Se sono state effettuate biopsie o rimozioni di polipi, i risultati di laboratorio completi potrebbero richiedere alcuni giorni.
   La colonscopia è un esame chiave per la prevenzione del cancro del colon-retto e per la diagnosi di altre condizioni intestinali.

OUTPUT (SESSION 2): La colonscopia è una procedura diagnostica che consente di esaminare l'interno del colon e del retto per identificare eventuali anomalie, come polipi, infiammazioni, o tumori. Ecco in cosa consiste esattamente:

1. Preparazione dell'intestino: Nei giorni che precedono la colonscopia, è necessario seguire una dieta speciale, spesso a base di liquidi chiari, e assumere lassativi. Questa preparazione è fondamentale per assicurare che l'intestino sia completamente pulito per una corretta visualizzazione durante l'esame.
2. Sedazione: Prima di iniziare la procedura, viene generalmente somministrata una sedazione leggera per ridurre il disagio e l'ansia. Questo rende la procedura più tollerabile per il paziente.
3. Esecuzione della procedura: Durante la colonscopia, un tubo flessibile chiamato colonscopio, dotato di una videocamera e una fonte di luce, viene inserito delicatamente nell'ano e fatto avanzare attraverso il retto e il colon.
4. Ispezione del colon: Il medico osserva l'immagine trasmessa dalla videocamera su un monitor per individuare eventuali anomalie. Durante il procedimento, il colon può essere leggermente gonfiato con aria o anidride carbonica per migliorare la visualizzazione delle pareti intestinali.
5. Rimozione di polipi e biopsie: Se vengono individuati polipi o altre anomalie, possono essere rimossi o biopsiati (prelevati piccoli campioni di tessuto) per l'analisi successiva in laboratorio.
6. Conclusione e recupero: Una volta completato l'esame, il colonscopio viene ritirato. Il paziente viene quindi tenuto in osservazione per un breve periodo mentre la sedazione svanisce. Potrebbe esserci una sensazione temporanea di gonfiore o crampi addominali, dovuta all'aria introdotta nel colon.
7. Discussione dei risultati: Il medico discuterà immediatamente i risultati preliminari con il paziente e fornirà ulteriori dettagli una volta disponibili i risultati delle eventuali biopsie.

La colonscopia è uno strumento essenziale per la prevenzione e la diagnosi precoce del cancro del colon-retto, ed è anche utile per valutare altre malattie intestinali.

Text similarity: 85%

3) **INPUT: La colonscopia è un esame rischioso?**

OUTPUT (SESSION 1): La colonscopia è generalmente considerata una procedura sicura e viene eseguita di routine per lo screening e la diagnosi di condizioni del colon, compreso il cancro del colon-retto. Tuttavia, come con qualsiasi procedura medica, comporta alcuni rischi. Ecco i potenziali rischi associati alla colonscopia:

1. Perforazione del colon: Anche se raro, c'è un piccolo rischio che il colonscopio possa perforare la parete del colon, che richiederebbe un intervento chirurgico per essere riparato.
2. Sanguinamento: Può verificarsi sanguinamento, soprattutto se vengono rimossi polipi o effettuate biopsie. Solitamente, questo tipo di sanguinamento è minore e si risolve da solo o può essere controllato durante la procedura.
3. Reazioni alla sedazione: Gli effetti collaterali da sedazione possono includere problemi respiratori o reazioni avverse, ma sono eventi rari. Prima della procedura, il team medico valuta il tuo stato di salute per minimizzare questo rischio.
4. Infezioni: Anche se molto rare, possono verificarsi infezioni locali a seguito della procedura.

5. Dolore o disagio addominale: Dopo la procedura è comune avvertire qualche crampo o gonfiore dovuto all'aria introdotta nel colon, ma questi sintomi di solito scompaiono rapidamente.
   È importante discutere con il medico i benefici e i rischi specifici della colonscopia nel tuo caso, specialmente se hai condizioni mediche particolari o preoccupazioni specifiche. Tuttavia, per la maggior parte delle persone, i benefici dello screening e della diagnosi precoce del cancro del colon-retto attraverso la colonscopia superano di gran lunga i potenziali rischi.

OUTPUT (SESSION 2): La colonscopia è considerata una procedura sicura ed efficace per la diagnosi e la prevenzione del cancro del colon-retto e di altre malattie intestinali. Tuttavia, come con qualsiasi procedura medica, ci sono alcuni rischi associati. Ecco i principali:

1. Perforazione intestinale: Un rischio raro, ma serio, durante una colonscopia è la perforazione della parete intestinale. Questo evento è molto raro e spesso richiede un intervento chirurgico per essere riparato.
2. Sanguinamento: Può verificarsi sanguinamento dopo la rimozione di un polipo o una biopsia. Solitamente, questo sanguinamento è lieve e si risolve da solo, ma in alcuni casi, potrebbe essere necessario un trattamento.
3. Reazioni alla sedazione: Sebbene rare, alcune persone possono avere reazioni avverse ai farmaci utilizzati per la sedazione.
4. Infezioni: Anche se estremamente rare, c'è un piccolo rischio di infezioni seguenti la procedura.
   È importante discutere questi rischi con il proprio medico prima della procedura. Nonostante i potenziali rischi, i benefici della colonscopia, in termini di diagnosi precoce e prevenzione del cancro del colon, superano di gran lunga i rischi associati nella maggior parte dei casi. Il tuo medico può aiutarti a valutare i benefici e i rischi personali e determinare se la colonscopia è appropriata per te.

Text similarity: 83%

4) **INPUT: La colonscopia è una procedura dolorosa?**

OUTPUT (SESSION 1): La colonscopia è generalmente ben tollerata dalla maggior parte dei pazienti e non è considerata una procedura dolorosa, grazie all'uso di sedativi e analgesici. Ecco cosa aspettarsi riguardo al dolore e al disagio associati alla colonscopia:

1. Sedazione: Durante la colonscopia, vengono somministrati sedativi e, in alcuni casi, analgesici per aiutare i pazienti a rilassarsi e a non sentire dolore. La maggior parte delle persone è in uno stato di sonnolenza o non ricorda gran parte della procedura.
2. Disagio minimo: Alcuni pazienti possono avvertire un lieve disagio o crampi addominali durante o dopo la procedura, principalmente a causa dell'aria introdotta nel colon per distenderlo e migliorare la visibilità.
3. Sensazione di gonfiore: È comune sentire gonfiore o pressione durante e subito dopo la procedura, ma questi sintomi di solito passano rapidamente una volta che l'aria viene espulsa.
4. Recupero rapido: Dopo la procedura, il paziente viene monitorato fino a quando gli effetti della sedazione non sono svaniti, solitamente entro un paio d'ore.
   Per molte persone, l'anticipazione della procedura è spesso più stressante del procedimento stesso, grazie alle misure di comfort fornite dall'anestesista e dal personale medico. È importante condividere eventuali preoccupazioni riguardanti il

dolore o il disagio con il tuo medico prima della colonscopia, in modo da poter adeguare le cure alle tue esigenze.

OUTPUT (SESSION 2): La colonscopia è generalmente ben tollerata e non è considerata una procedura dolorosa grazie all'uso di sedativi e/o analgesici somministrati prima e durante l'esame. Questi farmaci aiutano a rilassare il paziente e a ridurre eventuali disagi.
Durante la procedura, alcuni pazienti potrebbero avvertire:

- Sensazione di pressione o pienezza: Questo può essere dovuto all'introduzione di aria o anidride carbonica nel colon per espandere le pareti e migliorare la visibilità.
- Leggeri crampi: Potrebbero verificarsi crampi temporanei quando il colonscopio viene fatto avanzare o quando l'aria viene introdotta.

La maggior parte delle persone che si sottopongono a una colonscopia ricorda poco o niente della procedura a causa della sedazione, e il recupero dalla sedazione è generalmente rapido. È importante discutere con il medico eventuali preoccupazioni relative al dolore o al disagio, in modo che possano essere affrontate adeguatamente e si possa garantire il massimo comfort durante la procedura.

Text similarity: 89%

5) **INPUT: La colonscopia è una procedura invasiva?**

OUTPUT (SESSION 1): Sì, la colonscopia è considerata una procedura invasiva. Essa consiste nell'introduzione di un colonscopio, un tubo flessibile dotato di telecamera e luce, attraverso il retto per esaminare l'intero colon e il retto. Essendo un esame che comporta l'ingresso di strumenti nel corpo, è classificato come invasivo.
Nonostante ciò, la colonscopia è un procedimento standard per il rilevamento e la prevenzione di condizioni serie, come il cancro del colon-retto, e per l'individuazione di polipi che potrebbero essere precancerosi. Grazie all'uso della sedazione, la procedura è generalmente ben tollerata e la maggior parte dei pazienti sperimenta solo un minimo disagio. I benefici della colonscopia, in termini di diagnosi precoce e possibilità di rimozione immediata di potenziali rischi, tendono a superare i rischi associati alla sua natura invasiva.

OUTPUT (SESSION 2): Sì, la colonscopia è considerata una procedura invasiva, poiché comporta l'inserimento di un colonscopio - un tubo flessibile con una videocamera all'estremità - attraverso l'ano e nel colon per esaminare l'interno del tratto intestinale. Nonostante la natura invasiva della procedura, la colonscopia è generalmente sicura e ben tollerata, soprattutto grazie all'uso di sedazione o anestesia leggera che aiuta a minimizzare il disagio e l'ansia per il paziente.
La sua invasività è significativamente compensata dai benefici che offre, come la possibilità di:

- Identificare e rimuovere polipi precancerosi.
- Diagnosticare rapidamente condizioni potenzialmente gravi, come il cancro del colon.
- Esaminare alterazioni o anomalie nella mucosa intestinale che potrebbero non essere visibili con altri test meno invasivi.

Il medico valuterà i potenziali rischi individuali e discuterà con te se la colonscopia è la procedura più adatta alle tue esigenze mediche e quale tipo di preparazione è necessaria per assicurare un esame sicuro e accurato.

Text similarity: 89%

6) **INPUT: Come devo prepararmi per sottopormi ad una colonscopia?**

OUTPUT (SESSION 1): La preparazione per una colonscopia è una parte fondamentale della procedura per garantire che il colon sia completamente pulito, consentendo al medico di visualizzare chiaramente l'interno dell'intestino. Ecco i passaggi tipici per prepararti alla colonscopia:

1. Consulta il Medico: Incontra il tuo medico per ricevere istruzioni specifiche. Ogni medico o struttura sanitaria potrebbe avere linee guida leggermente diverse.
2. Dieta a basso residuo: Nei giorni precedenti la colonscopia, potrebbe esserti consigliato di seguire una dieta a basso contenuto di fibre. Il giorno prima della procedura, di solito è richiesta una dieta liquida trasparente, come brodi, succhi senza polpa, tè, caffè senza latte e gelatina (non rossa o viola).
3. Assunzione di lassativi: Ti verrà prescritto un lassativo specifico per pulire l'intestino. Questo può essere una soluzione da bere, che porta a numerosi movimenti intestinali il giorno prima dell'esame. È importante seguire scrupolosamente le istruzioni su quando e come assumere i lassativi.
4. Digiuno: Non mangiare o bere nulla dopo una certa ora la sera prima dell'esame, di solito dalla mezzanotte, salvo diverse istruzioni del medico.
5. Farmaci: Discuti con il tuo medico del tuo elenco di farmaci attuale. Potrebbe essere necessario interrompere o modificare l'assunzione di alcuni farmaci, specialmente quelli che influenzano la coagulazione del sangue o gli integratori a base di ferro.
6. Pianifica il Trasporto: Organizza qualcuno che possa accompagnarti a casa dopo la procedura, poiché la sedazione comprometterà la tua capacità di guidare veicoli.

Seguire attentamente queste istruzioni è cruciale per il successo della colonscopia. Una preparazione inadeguata potrebbe portare a una visualizzazione incompleta, rendendo necessario ripetere l'esame. Se hai dubbi o difficoltà nel seguire le istruzioni, contatta il tuo medico per ricevere supporto.

OUTPUT (SESSION 2): La preparazione per una colonscopia è fondamentale per assicurare che l'intestino sia completamente pulito, permettendo al medico di ottenere una visione chiara dell'interno del colon. Ecco i passaggi tipici per prepararsi adeguatamente:

1. Dieta speciale: Nei giorni precedenti l'esame, potrebbe essere richiesto di seguire una dieta a basso contenuto di fibre e di evitare cibi solidi la giornata prima della procedura. Il giorno prima dell'esame, di solito viene raccomandata una dieta liquida chiara, che può includere:
   - Brodo senza grassi
   - Succhi di frutta senza polpa (come succo di mela)
   - Gelatina
   - Tè o caffè (senza latte o panna)
   - Acqua
2. Assunzione di lassativi: Il medico ti prescriverà un lassativo specifico da assumere la sera prima della colonscopia (e talvolta anche la mattina del giorno della procedura). Questo aiuta a pulire completamente l'intestino. È importante seguire esattamente le istruzioni fornite.
3. Idratazione: È fondamentale bere molti liquidi per evitare la disidratazione a causa dei lassativi. Bevi liquidi chiari come acqua, brodo e succhi trasparenti fino a un paio d'ore prima dell'esame, come indicato dal tuo medico.

4. Farmaci: Consulta il tuo medico su quali farmaci puoi continuare a prendere. Alcuni medicinali, in particolare anticoagulanti e farmaci antinfiammatori, potrebbero necessitare di aggiustamenti.
5. Pianificazione logistica: Poiché verrà utilizzata la sedazione, avrai bisogno che qualcuno ti accompagni a casa dopo la procedura. Pianifica un pomeriggio libero dal lavoro o da altre attività, poiché potresti sentirti stordito o affaticato.
   Assicurati di seguire le istruzioni specifiche fornite dal tuo medico o dalla struttura sanitaria, poiché le raccomandazioni possono variare. Una preparazione accurata è essenziale per un esame efficace e sicuro.

   Text similarity: 90%

7) **INPUT: In cosa consiste la preparazione alla colonscopia?**

   OUTPUT (SESSION 1): La preparazione alla colonscopia è un processo essenziale per pulire completamente il colon, in modo che il medico possa esaminare chiaramente la sua mucosa interna. Ecco in cosa consiste tipicamente la preparazione:

1. Dieta a basso residuo: Nei giorni che precedono la colonscopia, potrebbe essere consigliata una dieta a basso contenuto di fibre, per limitare la formazione di residui nel colon.
2. Dieta liquida chiara: Il giorno prima dell'esame, dovrai passare a una dieta completamente liquida e chiara. Questa può includere:
   - Brodi chiari senza solidi.
   - Bevande trasparenti come tè, caffè senza latte e succhi di frutta chiari senza polpa.
   - Gelatine non rosse o viola.
   - Acqua e bevande sportive chiare.
3. Assunzione di lassativi: Ti verrà prescritta una soluzione lassativa specifica per pulire l'intestino. Questo di solito comporta:
   - Bere una grande quantità di liquido lassativo la sera prima della colonscopia, che provoca frequenti movimenti intestinali per svuotare completamente il colon.
   - A volte può essere necessario bere una dose aggiuntiva la mattina dell'esame.
4. Digiuno: Solitamente, non è consentito mangiare o bere nulla, ad eccezione di piccoli sorsi d'acqua per assumere farmaci, dopo la mezzanotte precedente all'esame.
5. Farmaci: Parla con il tuo medico riguardo ai farmaci che stai assumendo. Potrebbe essere necessario interrompere o modificare alcuni di essi, specialmente quelli che influenzano la coagulazione del sangue.
6. Regole specifiche: Segui tutte le indicazioni specifiche fornite dal medico o dal centro sanitario dove la colonscopia sarà effettuata, poiché alcuni dettagli possono variare.
   È importante attenersi rigorosamente a queste istruzioni per garantire una visualizzazione chiara e accurata durante la colonscopia. Un colon non completamente pulito potrebbe portare a una procedura incompleta, rendendo necessaria una nuova colonscopia. Se hai domande o riscontri difficoltà nel processo di preparazione, contatta il tuo medico per assistenza.

   OUTPUT (SESSION 2): La preparazione alla colonscopia è un processo fondamentale per garantire che l'intestino sia pulito e chiaramente visibile durante la procedura. Ecco come si svolge generalmente la preparazione:

1. Dieta a basso residuo: Alcuni giorni prima dell'esame, il medico potrebbe consigliarti di seguire una dieta a basso contenuto di fibre per ridurre la quantità di residui nell'intestino.
2. Dieta liquida chiara: Il giorno prima della colonscopia, si passa a una dieta composta da soli liquidi chiari. Questo include:
    - Brodo senza grassi
    - Succhi di frutta senza polpa (come succo di mela o uva bianca)
    - Gelatina senza coloranti rossi o viola
    - Bevande sportive chiare
    - Acqua
    - Tè o caffè (senza latte o panna)
3. Assunzione di lassativi: Il medico prescriverà un lassativo specifico, che aiuta a svuotare completamente l'intestino. Questo può essere una soluzione da bere in due parti: una la sera prima e un'altra la mattina della procedura, a seconda delle istruzioni del medico.
4. Idratazione: È importante mantenersi ben idratati bevendo molti liquidi chiari. Questo aiuta anche a compensare i liquidi persi durante la fase di pulizia intestinale.
5. Farmaci: Consulta il tuo medico riguardo i farmaci che assumi regolarmente. Potrebbe essere necessario sospendere temporaneamente alcuni farmaci, come quelli che influenzano la coagulazione del sangue.
6. Pianificazione del viaggio: Poiché verrà somministrata la sedazione durante la colonscopia, avrai bisogno di qualcuno che ti accompagni a casa dopo l'esame.

   Comunicare eventuali dubbi o preoccupazioni al tuo medico è importante per una preparazione efficace e per garantire che la colonscopia sia condotta in modo sicuro e ottimale.

Text similarity: 86%

8) **INPUT: Come avviene la preparazione alla colonscopia?**

OUTPUT (SESSION 1): La preparazione alla colonscopia è fondamentale per garantire che il medico possa avere una visione chiara del colon. Di solito, la preparazione comprende i seguenti passi:

1. Dieta a basso residuo: Qualche giorno prima dell'esame, il medico potrebbe consigliare una dieta a basso contenuto di fibre per ridurre la quantità di feci nel colon.
2. Dieta liquida: Il giorno prima della colonscopia, ti verrà chiesto di seguire una dieta a base di soli liquidi chiari. Questo include acqua, brodo, succhi di frutta filtrati (senza polpa), tè, caffè (senza latte o panna) e gelatina (senza coloranti rossi o viola).
3. Assunzione di lassativi: Solitamente, il medico prescrive un potente lassativo da assumere il giorno prima dell'esame. Questo aiuta a svuotare completamente l'intestino. Il tipo di lassativo e il dosaggio dipendono dalle istruzioni specifiche del medico.
4. Idratazione: È importante bere molti liquidi chiari durante la preparazione per prevenire la disidratazione.
5. Modifiche ai farmaci: Parla con il tuo medico circa eventuali farmaci che stai assumendo. Potrebbe essere necessario interrompere o aggiustare alcuni di essi, specialmente se prendi anticoagulanti o farmaci per il diabete.

   Segui attentamente le istruzioni del tuo medico per assicurarti che l'esame venga eseguito correttamente. Se hai dubbi o incontri difficoltà durante la preparazione, contatta il tuo medico per chiarimenti.

OUTPUT (SESSION 2): La preparazione alla colonscopia è fondamentale per garantire che l'intestino sia completamente pulito e privo di residui, permettendo così una visibilità ottimale durante l'esame. Ecco un quadro generale di come avviene la preparazione:

1. Dieta speciale:
   - Giorni precedenti: Nei giorni che precedono la colonscopia, potrebbe essere consigliato di seguire una dieta a basso residuo, riducendo il consumo di fibre.
   - Il giorno prima: Passa a una dieta liquida chiara. Questo include consumare solo:
     - Brodo chiaro (senza grassi)
     - Succhi di frutta chiari e senza polpa (come il succo di mela)
     - Gelatina (evitando colori rosso e viola)
     - Tè o caffè (senza latte o panna)
     - Bevande sportive trasparenti
     - Acqua
2. Assunzione di lassativi:
   - Soluzione lassativa: Il medico prescriverà una soluzione lassativa, che deve essere assunta secondo le istruzioni specifiche. Spesso è divisa in due dosi: una la sera prima e una al mattino presto il giorno dell'esame.
   - Compresse lassative (in alcuni protocolli): Possono essere prescritte da assumere insieme alla soluzione lassativa.
3. Idratazione:
   - È importante bere molti liquidi chiari durante la preparazione per evitare la disidratazione e per aiutare nella pulizia dell'intestino.
4. Medicazioni:
   - Consulta il tuo medico su eventuali farmaci che stai assumendo. Potrebbe essere necessario interrompere temporaneamente o modificare l'uso di farmaci, soprattutto quelli che influenzano la coagulazione del sangue.
5. Ulteriori istruzioni e consigli:
   - Gli effetti del lassativo inizieranno a manifestarsi dopo poche ore dall'assunzione, quindi assicurati di avere accesso a un bagno.
   - Indossa abiti comodi e pianifica di rimanere a casa durante questa fase.

Seguendo attentamente le istruzioni del tuo medico per la preparazione, aiuti a garantire che la procedura si svolga nel modo più efficace e sicuro possibile. Se hai domande o dubbi, assicurati di discuterne con il tuo medico prima della colonscopia.

Text similarity: 93%

9) INPUT: Per sottopormi ad una colonscopia devo seguire delle istruzioni alimentari in aggiunta all'assunzione del lassativo?

OUTPUT (SESSION 1): Sì, oltre all'assunzione di un lassativo, è importante seguire specifiche istruzioni alimentari per prepararti adeguatamente a una colonscopia. Ecco cosa generalmente viene raccomandato:

1. Qualche giorno prima dell'esame:
   - Inizia una dieta a basso contenuto di fibre per ridurre la quantità di feci.
2. Il giorno prima della colonscopia:
   - Segui una dieta a base di soli liquidi chiari. Questo comprende:
     - Acqua
     - Brodo
     - Succhi di frutta filtrati (come mela o uva bianca, senza polpa)

    - Bevande sportive
    - Gelatina (senza coloranti rossi o viola)
    - Tè e caffè (senza latte o panna)
3. Il giorno dell'esame:
    - Solitamente, ti sarà chiesto di non mangiare o bere nulla dopo la mezzanotte, eccetto piccole quantità di acqua necessarie per assumere eventuali farmaci.

È cruciale seguire attentamente le istruzioni fornite dal tuo medico, poiché una preparazione inadeguata potrebbe influire sulla qualità dell'esame, rendendo difficile l'identificazione di eventuali anomalie. Se hai domande specifiche o se le istruzioni non sono chiare, contatta il tuo medico per ulteriori chiarimenti.

OUTPUT (SESSION 2): Sì, per sottoporsi a una colonscopia, è fondamentale seguire specifiche istruzioni alimentari in aggiunta all'assunzione del lassativo, per garantire che l'intestino sia completamente pulito. Ecco i passaggi generali:

1. Giorni precedenti:
    - Dieta a basso contenuto di fibre: Nei giorni che precedono la procedura, il medico potrebbe consigliarti di evitare cibi ricchi di fibre, come frutta fresca, verdure crude, cereali integrali e semi. Ridurre questi alimenti aiuta a minimizzare i residui nell'intestino.
2. Il giorno prima della colonscopia:
    - Dieta liquida chiara: Passa a una dieta composta solo da liquidi chiari. Puoi consumare:
        - Brodo chiaro (senza pezzi di carne o verdure)
        - Succhi di frutta senza polpa, come succo di mela o di uva bianca
        - Gelatina chiara (evitando i colori rosso e viola)
        - Bevande sportive chiare e trasparenti
        - Tè o caffè (senza latte o panna)
        - Acqua
3. Assunzione del lassativo:
    - Segui le indicazioni del tuo medico per l'assunzione del lassativo, spesso iniziandolo il pomeriggio o la sera prima della procedura.
4. Il giorno della colonscopia:
    - Continua a bere liquidi chiari fino a un paio d'ore prima dell'esame, secondo le istruzioni del tuo medico, per rimanere idratato.

Queste istruzioni alimentari sono essenziali per permettere una pulizia efficace dell'intestino, garantendo così una colonscopia accurata e sicura. Seguire le indicazioni del medico o del personale sanitario è cruciale per il successo della procedura.

Text similarity: 90%

10) INPUT: Viene eseguita una anestesia durante la colonscopia?

OUTPUT (SESSION 1): Sì, durante una colonscopia viene solitamente somministrata una forma di sedazione o anestesia per garantire comfort e ridurre il disagio durante la procedura. Ecco le opzioni comuni:

1. Sedazione cosciente: Questo tipo di sedazione ti rilassa e allevia il dolore, mentre rimani cosciente. Solitamente si utilizzano farmaci somministrati per via endovenosa.
2. Anestesia profonda: Ti porta in un sonno profondo durante il quale non sei cosciente. Anche in questo caso, i farmaci vengono somministrati per via endovenosa.

3. Anestesia generale (raramente usata): In alcuni casi, si può optare per un'anestesia generale, che ti induce a un sonno completo, ma è meno comune per le colonscopie di routine.
   Il tipo di sedazione scelto dipenderà da vari fattori, inclusi la tua salute generale, eventuali allergie o reazioni passate a farmaci sedativi, e le preferenze del medico e del paziente. È importante discutere con il tuo medico delle opzioni di sedazione disponibili per assicurarti di ricevere il livello di comfort e sicurezza adeguato durante l'esame.

OUTPUT (SESSION 2): Sì, durante una colonscopia viene generalmente somministrata una forma di sedazione o anestesia leggera per garantire il comfort e ridurre il disagio del paziente. Ecco le opzioni comuni:

1. Sedazione cosciente: Questa è la forma più comune di sedazione utilizzata durante una colonscopia. I farmaci vengono somministrati per via endovenosa, aiutando il paziente a rilassarsi e spesso a non ricordare la procedura. Pur essendo leggermente sedati, i pazienti possono essere in grado di rispondere a semplici comandi.
2. Sedazione profonda: Per alcuni pazienti o in base alle preferenze del medico, può essere utilizzata una sedazione più profonda, dove il paziente non è consapevole della procedura e non avverte alcun dolore. Anche in questo caso, i farmaci vengono somministrati per via endovenosa.
3. Anestesia generale: Quest'opzione viene raramente utilizzata per la colonscopia, ma può essere indicata in casi particolari quando richiesto da specifiche condizioni mediche o preferenze del paziente.
   Il tipo di sedazione utilizzata può dipendere da vari fattori, inclusi il tuo stato di salute generale, eventuali esperienze passate e le policy della struttura sanitaria. È importante discutere con il tuo medico quali opzioni di sedazione sono disponibili e più appropriate per te prima della procedura. Dopo una colonscopia con sedazione, è necessario farsi accompagnare a casa, poiché gli effetti sedativi possono durare alcune ore.

Text similarity: 89%

ENGLISH VERSION

SCENARIO 1
You have heard about colon cancer screening and want to learn more. Ask your question to get clarification.

1) **INPUT: What does colon cancer screening mean?**

OUTPUT (SESSION 1): Colon cancer screening refers to a set of tests and procedures used to detect colorectal cancer in people who have no symptoms. The goal is to detect cancer at an early stage, when is more treatable and has better chances of cure, or identify and remove precancerous polyps before they turn into malignant tumors.
There are several types of screening tests for colon cancer, including:

1. Colonoscopy: A procedure in which a doctor uses an endoscope to examine the inside of the colon and rectum. During a colonoscopy, polyps may be removed or tissue samples may be taken for further analysis.
2. Virtual colonoscopy (or CT colonography): A radiological test that uses computed tomography to obtain images of the colon.
3. sigmoidoscopy : Similar to colonoscopy but limits the examination to the lower part of the colon.
4. Fecal occult blood test (FOBT) or fecal immunochemical test (FIT): These tests look for traces of blood in the stool, a possible indicator of cancer or polyps.
5. Fecal DNA test (e.g. Cologuard): Looks for genetic alterations in colon slough cells that pass through stool, as well as traces of blood.

The choice of test depends on several factors including age, family history of cancer, personal risk factors, and patient preference. It is important to consult with a doctor to decide on the most appropriate screening program.

OUTPUT (SESSION 2): Colon cancer screening is a set of medical tests and procedures used to detect colorectal cancer in people who have no symptoms. The goal is to identify precancerous polyps or cancers at an early stage, when they are more easily treated. Common screening tests include:

1. Colonoscopy: A direct visual examination of the inside of the colon using a flexible tube with a camera.
2. sigmoidoscopy : Similar to colonoscopy but only examines the lower part of the colon.
3. Fecal occult blood test (FOBT): A test that detects traces of blood in the stool, which may indicate the presence of polyps or tumors.
4. Fecal DNA test: Analyzes stool to detect genetic mutations associated with colon cancer.
5. Virtual colonoscopy (CT colonography): A CT scan that produces detailed images of the colon and rectum.

Regular screening is generally recommended starting at age 45-50, but may be started earlier for those with high risk factors. It is important to discuss with a doctor to determine which test is most appropriate based on personal and family history.

Text similarity: 87%

2) **INPUT: What is colon cancer screening?**

OUTPUT (SESSION 1): Colon cancer screening refers to a series of medical tests designed to detect colorectal cancer early in people who have no symptoms. The goal of screening is to find cancer at an early stage, when treatment is most effective and the chances of success are greatest. Screening also helps find and remove precancerous polyps before they can turn into cancer.
Here are the main screening methods used:

1. Colonoscopy: Examines the entire colon and rectum using a flexible tube with a camera. Can detect and remove polyps.
2. Virtual colonoscopy (CT colonography): Uses computed tomography to create detailed images of the colon.
3. sigmoidoscopy : Examines only the lower part of the colon and rectum.
4. Fecal occult blood test (FOBT) and fecal immunochemical test (FIT): Detect blood in the stool, which may indicate the presence of polyps or cancer.
5. Fecal DNA test: Analyzes stool to detect genetic changes associated with colorectal cancer.

It is important to discuss with a doctor which screening method is best based on your age, family history, risk factors, and individual preferences.

OUTPUT (SESSION 2): Colon cancer screening refers to a series of tests and medical procedures that are performed to detect colorectal cancer early in asymptomatic individuals. The primary goal is to identify both precancerous polyps, which can progress to cancer, and cancers in their early stages, when they are most easily treated and curable. Common screening methods include:

1. Colonoscopy: A direct visual examination of the colon using a flexible tube equipped with a camera, which allows polyps to be found and removed.
2. sigmoidoscopy : Similar to colonoscopy, but focuses on examining the lower part of the colon.
3. Fecal occult blood test (FOBT): Checks for the presence of non-visible blood in the stool, which may be an indicator of polyps or cancer.
4. Fecal DNA test: Analyzes stool to detect genetic changes associated with colon cancer.
5. colonography (virtual colonoscopy): Uses CT scans to produce images of the colon and rectum.

The recommendation is that adults begin routine screening between the ages of 45 and 50, depending on national guidelines and individual risk factors, such as family history of colon cancer. Talking to a doctor is essential to determine the most appropriate screening plan.

Text similarity: 90%

3) **INPUT: What is colon cancer screening?**

OUTPUT (SESSION 1): Colon cancer screening is a series of tests aimed at identifying colorectal cancer or precancerous polyps early in asymptomatic people. The main goal is to detect the disease at an early stage, thus increasing the chances of effective treatment and success. The main screening methods include:

1. Colonoscopy: A procedure in which a doctor uses an endoscope to examine the entire colon and rectum. During the exam, polyps may be removed or tissue samples may be taken for further analysis.

2. Virtual colonoscopy (CT colonography): A noninvasive test that uses computed tomography to obtain detailed images of the colon and rectum.
3. sigmoidoscopy : This procedure is similar to a colonoscopy but only examines the lower part of the colon and rectum.
4. Fecal occult blood test (FOBT) and fecal immunochemical test (FIT): Tests that check for the presence of blood in the stool, a potential indicator of polyps or cancer.
5. Fecal DNA test: Analyzes DNA found in colon sloughed cells in the stool to detect genetic changes that may indicate the presence of tumors or polyps.
   The type of screening test chosen depends on several factors, including the patient's age, family history, personal risk factors, and individual preferences. It is always advisable to discuss with your doctor to determine the most appropriate screening path.

   OUTPUT (SESSION 2): Colon cancer screening consists of a series of tests designed to detect colorectal cancer and precancerous polyps early. These tests are performed on people who have no symptoms of cancer, with the goal of identifying the disease when it is in its earliest, most treatable stages. The main screening methods include:

1. Colonoscopy: A procedure in which a doctor examines the inside of the colon using a flexible tube with a camera. During a colonoscopy, polyps may be removed or biopsies may be taken.
2. sigmoidoscopy : A test similar to a colonoscopy, but which examines only the lower part of the colon and rectum.
3. Fecal occult blood test (FOBT): A test that checks for the presence of non-visible blood in the stool, which may indicate the presence of polyps or tumors.
4. Fecal DNA test: A test that detects specific genetic mutations that may be a sign of colon cancer.
5. Colonography (Virtual Colonoscopy): An imaging test that uses a CT scan to produce detailed images of the colon and rectum.
   The choice of test depends on several factors, including age, family history of cancer, and other risk factors. In general, it is recommended that adults begin screening between the ages of 45 and 50, unless there is a reason to start earlier. Consulting with a doctor can help determine the best personalized screening plan.

   Text similarity: 87%

4) **INPUT: How is colon cancer screening done?**

   OUTPUT (SESSION 1): Colon cancer screening can be done through several tests, each with its own method of execution. Here is how the most common ones work:

1. Colonoscopy:
   - Preparation: It requires bowel preparation which consists of following a specific diet and taking laxatives to clean the colon.
   - Procedure: A doctor inserts a colonoscope (a flexible tube with a camera) through the rectum to examine the entire colon. During the exam, any polyps may be removed and sent to a laboratory for analysis.
2. Virtual colonoscopy (CT colonography):
   - Preparation: Similar to a colonoscopy, with a bowel cleansing.
   - Procedure: Uses a computerized tomography scan to take pictures of the large intestine. Does not require sedation, but may be less effective at detecting small polyps.

3. Flexible sigmoidoscopy :
    - Preparation: Usually a cleansing of the rectum and lower colon is necessary.
    - Procedure: Similar to a colonoscopy but only examines the rectum and sigmoid colon.
4. Fecal occult blood test (FOBT) and fecal immunochemical test (FIT):
    - Preparation: Often does not require dietary restrictions or stopping medications, but it is important to follow the test instructions.
    - Procedure: The patient provides a stool sample which is analyzed for traces of blood not visible to the naked eye.
5. Fecal DNA test:
    - Preparation: No specific bowel preparation is required.
    - Procedure: The patient collects a stool sample at home and sends it to a laboratory, where it is analyzed to identify specific genetic mutations and the presence of blood.

The choice of test depends on several individual factors, including age, personal risk, and preferences, and should be discussed with your doctor.

OUTPUT (SESSION 2): Colon cancer screening is performed through a series of tests that allow early detection of cancer or precancerous polyps in the colon and rectum. Here is a description of how the main screening tests are performed:

1. Colonoscopy:
    - Preparation: In the days leading up to the procedure, a special diet and the use of laxatives may be required to completely cleanse the intestines.
    - Procedure: During the exam, a doctor inserts a colonoscope (a flexible tube with a camera) into the anus and guides it through the colon. This allows the doctor to view the inside of the colon and detect any polyps or abnormalities. If necessary, the doctor can remove polyps or take tissue samples for a biopsy.
2. Flexible sigmoidoscopy :
    - Preparation: Similar to a colonoscopy, but usually less intense.
    - Procedure: An instrument similar to a colonoscopy is used, but the scope is limited to the lower part of the colon. This test is quicker and less invasive.
3. Fecal occult blood test (FOBT):
    - Preparation: It requires the adoption of a specific diet a few days before the test to avoid false results.
    - Procedure: The patient collects stool samples at home, which are then sent to a laboratory to check for blood that is not visible to the naked eye.
4. Fecal DNA test:
    - Procedure: Similar to FOBT, you collect stool samples at home. The lab analyzes the stool for genetic mutations or markers of colon cancer.
5. colonography (virtual colonoscopy):
    - Preparation: Similar to a traditional colonoscopy, the colon must be cleaned.
    - Procedure: During a CT scan, you lie on a bed while an X-ray machine takes pictures of your colon. This test is less invasive, but if it finds any abnormalities, a traditional colonoscopy may be necessary.

Each test has its advantages and limitations, and the choice of the most suitable method depends on the individual patient's condition, the availability of the tests, and the doctor's recommendations.

Text similarity: 88%

5) **INPUT: How is colon cancer screening performed?**

OUTPUT (SESSION 1): Colon cancer screening can be performed through various tests, each with a specific procedure. Here is an overview of the main methods:

1. Colonoscopy:
   - Preparation: Patients must follow a specific diet and take laxatives the day before to clean the bowels.
   - Procedure: A doctor inserts a colonoscope, which is a flexible tube with a camera, through the rectum into the colon to view the entire intestinal tract. Any polyps or tissue samples may be removed or taken for further testing.
2. Virtual colonoscopy (CT colonography):
   - Preparation: Similar to a colonoscopy with a complete cleansing of the colon.
   - Procedure: A CT scan is performed after insufflation of air or CO2 into the colon to obtain detailed images. It does not require sedation and allows detailed visualization of the colon.
3. Flexible sigmoidoscopy :
   - Preparation: Usually requires a partial cleansing of the lower colon.
   - Procedure: A sigmoidoscope , similar to a colonoscope but shorter, is inserted into the rectum to examine the rectum and sigmoid colon.
4. Fecal occult blood test (FOBT) and fecal immunochemical test (FIT):
   - Preparation: Generally does not require specific preparations.
   - Procedure: The patient collects a small stool sample at home, which is then tested in a laboratory for occult blood, which can indicate the presence of polyps or cancer.
5. Fecal DNA test:
   - Preparation: No special bowel preparation is required.
   - Procedure: The patient collects a stool sample at home. This is sent to a laboratory, where it is analyzed for specific genetic alterations associated with colorectal cancer.

These tests are designed to detect colorectal cancer early or identify its precursors, thus increasing the chances of effective treatment. The choice of the most appropriate test should be made in collaboration with a doctor, based on individual risks and patient preferences.

OUTPUT (SESSION 2): Colon cancer screening is done using a series of tests designed to detect precancerous polyps and tumors in the colon and rectum before they cause symptoms. Here's how the main screening tests are done:

1. Colonoscopy:
   - Preparation: In the days preceding the exam, a specific diet and the intake of laxatives are required to completely clean the intestine.
   - Procedure: A colonoscope (a flexible tube with a camera) is inserted into the anus and guided through the colon to view the inside and detect any abnormalities. During the procedure, the doctor may remove polyps or take samples for a biopsy.
2. Flexible sigmoidoscopy :
   - Preparation: Requires less intensive cleaning than a full colonoscopy.
   - Procedure: A similar tube is inserted to examine only the lower part of the colon. This test is usually quicker and can be done without sedation.
3. Fecal occult blood test (FOBT):
   - Preparation: Specific dietary guidelines may be followed for a few days.
   - Procedure: Patients collect stool samples at home, which are then tested in a laboratory to detect nonvisible blood.

4. Fecal DNA test:
    - Procedure: Stool samples are collected at home and analyzed in the laboratory for genetic mutations linked to colon cancer.
5. colonography (virtual colonoscopy):
    - Preparation: Similar to a traditional colonoscopy, it requires cleansing the colon.
    - Procedure: The patient lies on a bed in a machine that takes CT scans to take detailed images of the colon. If polyps or other abnormalities are found, further examination with a traditional colonoscopy may be required.

Each test has its pros and cons, and the choice of which one to take depends on a variety of factors, including your age, family history, and your personal medical conditions. A doctor can help determine which screening test is best for you.

Text similarity: 86%

6) **INPUT: What is colon cancer screening for?**

OUTPUT (SESSION 1): Colon cancer screening has the primary goal of detecting the disease in early stages, when it is more treatable and the chances of cure are greater. Here is what it is used for specifically:

1. Early detection: Detecting colorectal cancer at an early stage, before obvious symptoms appear. This allows for timely intervention, improving the chances of effective treatment and reducing the risk of mortality.
2. Prevention: Identify and remove precancerous polyps (adenomas), which are growths in the colon that could develop into cancer if left untreated. Removing them helps prevent the development of cancer.
3. Reduction in mortality: Statistics and studies have shown that regular screening reduces the mortality rate associated with colorectal cancer, thanks to the possibility of treating the disease before it becomes advanced.
4. Treatment Guide: Screening results can help determine the best course of treatment, which may include surgery, chemotherapy, or other forms of treatment.

In summary, screening is an important preventive measure that helps improve the health and longevity of the population, allowing colon cancer to be addressed more successfully.

OUTPUT (SESSION 2): Colon cancer screening is used to detect the early presence of precancerous tumors or polyps in the colon and rectum. The primary goal of screening is to detect these polyps or tumors at an early stage, when they are usually more treatable, and thus increase the chances of cure and survival.

The main benefits of screening include:

1. Prevention: Identifying and removing precancerous polyps can prevent the development of a malignant tumor.
2. Early diagnosis: Detecting colorectal cancer in its early stages allows for more effective and less invasive treatments, significantly increasing the chances of recovery.
3. Reduction in mortality: Regular screening has been shown to reduce the mortality rate associated with colon cancer, as treatments are more effective in the early stages of the disease.
4. Awareness of risk factors: It can also help identify individuals with a genetic predisposition or other risk factors, who need more frequent monitoring and control.

Colorectal screening is generally recommended for adults starting at age 45 to 50, or earlier if there are high risk factors, such as a family history of colon cancer. Talking to a doctor can help determine the best screening plan and timing for your individual situation.

Text similarity: 90%

7) **INPUT: Why is colon cancer screening done?**

OUTPUT (SESSION 1): Colon cancer screening is performed for several key reasons:

1. Early detection: This allows colorectal cancer to be detected in early stages, when there may be no symptoms. Early identification facilitates more effective treatments and increases the chances of recovery.
2. Prevention: Screening helps identify and remove precancerous polyps (small growths in the colon), significantly reducing the risk of them turning into cancer.
3. Reduction of Mortality: Through early diagnosis and timely treatment, regular screening helps reduce the mortality rate associated with colorectal cancer. Numerous studies show that screening lowers the likelihood of death from this type of cancer.
4. Early intervention: Identifying cancer before it spreads allows for the most appropriate treatment, which may include surgery, chemotherapy, or other targeted therapies.
5. Health Awareness: Participating in regular screening programs encourages people to be proactive about their health, leading them to have regular medical check-ups and discuss any risk factors with their doctors.

In summary, colon cancer screening is an important public health strategy designed to prevent disease and save lives.

OUTPUT (SESSION 2): Colon cancer screening is performed for several key reasons:

1. Prevention: It allows us to detect and remove precancerous polyps before they can develop into malignant tumors, thus reducing the risk of developing colorectal cancer.
2. Early diagnosis: Detect tumors in their early stages, when they are most treatable and the chances of therapeutic success are greatest.
3. Reduction of mortality: Through early diagnosis and timely treatment, screening helps reduce the mortality rate associated with colorectal cancer.
4. Improving treatment options: Treatment of cancers discovered early tends to be less invasive and more effective than those discovered at a later stage.
5. Identifying risk factors: Screening can detect individuals with increased genetic predisposition or other risk factors, allowing for closer surveillance and timely preventive interventions.

In summary, colon cancer screening is an important preventive measure that can save lives, improve quality of life, and reduce the costs associated with treating advanced cancer. It is generally recommended for adults starting at age 45-50, or earlier if there are particular risk factors.

Text similarity: 89%

8) **INPUT: Why is it advisable to screen for colon cancer?**

OUTPUT (SESSION 1): Colon cancer screening is advisable for several key reasons:

1. Early detection: Detects colorectal cancer in its earliest stages, when it is most treatable and has the highest chance of cure. Tumors detected early can often be completely removed with surgery.
2. Prevention: It can detect precancerous polyps, which are benign but potentially dangerous growths in the colon. Removing them during screening tests can prevent them from becoming cancerous.
3. Reduction in Mortality: Regular screening has been shown to be effective in reducing colorectal cancer-related mortality rates through timely diagnosis and treatment.
4. Reduced invasive treatments: Detecting cancer at an early stage can often limit the need for more invasive or extensive treatments, improving quality of life and reducing complications.
5. Awareness of Risk Factors: Participating in screening also provides an opportunity to discuss with your doctor your personal risk factors, such as family history and other health-related factors, which may influence how often and what type of screening you need.
6. Encouraging general health: Regular screening can encourage people to maintain a healthy lifestyle and be aware of their health, promoting broader preventive measures.

In summary, colon cancer screening is a crucial tool for the prevention and control of colorectal cancer, offering significant potential to reduce the incidence and mortality associated with this disease.

OUTPUT (SESSION 2): Colon cancer screening is recommended for several key reasons:

1. Prevention: Screening allows us to identify and remove precancerous polyps before they can turn into malignant tumors, reducing the risk of developing colorectal cancer.
2. Early diagnosis: Identify cancer in its early stages, when it is most treatable and the chances of cure are greatest.
3. Reduction of mortality: Through timely diagnosis and treatment, screening helps significantly reduce the mortality rate related to colon cancer.
4. Less invasive treatments: Tumors detected early can be treated with less invasive procedures and with greater therapeutic success.
5. Identification of risk factors: Screening can reveal a genetic predisposition or other risk factors, providing the opportunity for appropriate surveillance and preventive interventions.

In short, regular screening is a preventative measure that can save lives, improve quality of life, and reduce the costs associated with treating advanced cancer. Experts typically recommend starting screening at age 45 to 50, but if you have certain risk factors, you may need to start earlier. Talking to your doctor can help determine the best approach and screening program for your individual situation.

Text similarity: 87%

9) **INPUT: What is the utility of colon cancer screening?**

OUTPUT (SESSION 1): The usefulness of colon cancer screening lies mainly in the following aspects:

1. Early Detection: This allows colorectal cancer to be detected in its early stages, when treatment is usually most effective and the chances of cure are highest. A tumor detected early can often be successfully removed surgically.
2. Prevention: Screening can identify precancerous polyps in the colon, which can be removed before they become malignant tumors. This helps prevent the development of colorectal cancer.
3. Reduced Mortality: Studies show that regular screening reduces mortality rates associated with colorectal cancer, through early detection and rapid intervention.
4. Limiting Invasive Treatments: Detecting cancer at an early stage can reduce the need for more complex and prolonged invasive treatments, improving the quality of life for patients.
5. Awareness and Prevention: Screening encourages people to be more aware of their colon health and to discuss their risk of colorectal cancer with their doctors, promoting a healthier lifestyle.
6. Risk Factor Information: During screening visits, doctors can better counsel patients about their risk factors, tailoring recommendations based on family history, age, and other health factors.

In summary, colon cancer screening is an essential tool in the prevention and control strategy of colorectal cancer, helping to save lives and improve health experiences.

OUTPUT (SESSION 2): The utility of colon cancer screening lies in several key aspects that improve health management and save lives:

1. Prevention: Through screening, precancerous polyps can be identified and removed before they become malignant tumors, significantly reducing the risk of developing colon cancer.
2. Early Diagnosis: Detecting colon cancer in its early stages allows for more effective treatment than when the disease is in an advanced stage. Early diagnosis is associated with better survival rates.
3. Reduction of mortality: Regular screening has been shown to reduce mortality associated with colon cancer, thanks to the possibility of timely and appropriate treatment.
4. Less invasive interventions: Treatment of tumors detected in the early stages is often less invasive and more effective, with less impact on the patient's quality of life.
5. Identifying risk factors: Screening is useful for identifying individuals at high risk, such as those with a family history of colon cancer, who may benefit from more frequent monitoring.

Overall, colorectal screening is an important public health strategy that helps prevent the development of colon cancer and improves treatment options and outcomes for those who do develop it. It is generally recommended for adults ages 45-50 and older, unless there are specific risk factors that require a different approach.

Text similarity: 85%

10) INPUT: Who is eligible for colon cancer screening?

OUTPUT (SESSION 1): Colon cancer screening is generally aimed at:

1. Adults over 45: In many guidelines, the recommended age to start screening is 45, because of the increased risk of colorectal cancer with age.
2. People with a family history of colorectal cancer or polyps: Individuals with first-degree relatives who have had colon cancer or adenomatous polyps may need to start screening earlier than age 45 and more frequently.

3. Individuals with inherited conditions: Those with inherited syndromes, such as Lynch syndrome or familial adenomatous polyposis (FAP), which increase the risk of colon cancer, need closer monitoring and earlier screening.
4. People with inflammatory bowel disease: Those with diseases such as ulcerative colitis or Crohn's disease are at higher risk and may require more frequent screening.
5. Individuals who have had polyps or colon cancer in the past: These patients need regular checkups to prevent recurrence or detect early new growths.
6. People with other risk factors: Conditions such as obesity, a low-fiber, high-fat diet, and long-term alcohol and tobacco use can increase risk, making screening an important consideration.

   It is essential for each individual to discuss this with their doctor to determine the most appropriate timing and type of screening based on their personal situation and risk factors.

OUTPUT (SESSION 2): Colon cancer screening is generally aimed at:

1. Adults over 45-50 years old: Guidelines recommend that individuals begin regular screening for colon cancer starting in this age group, as the risk of developing the disease increases with age.
2. People with a family history of colon cancer: Those who have first-degree relatives (parents, siblings, or children) who have had colon cancer may be able to start screening before age 45, on the advice of a doctor.
3. Individuals with inherited conditions: Those who have genetic syndromes such as familial adenomatous polyposis (FAP) or Lynch syndrome (hereditary nonpolyposis colorectal cancer , HNPCC) are considered at high risk and may need early and frequent screening.
4. Patients with a personal history of colon polyps or cancer: People who have had precancerous polyps or colon cancer in the past should have regular checkups.
5. Individuals with inflammatory bowel disease: Diseases such as ulcerative colitis or Crohn's disease can increase the risk of colon cancer, making more frequent checkups necessary.

   It is important for each individual to discuss with their doctor to determine the right time to start and the frequency of screening, considering their personal and family risk factors.

Text similarity : 85%

SCENARIO 2
Want to participate in colon cancer screening but don't know how? Ask your question for clarification.

1) **INPUT: Who should I contact for more information about colon cancer screening?**

OUTPUT (SESSION 1): For more information on colon cancer screening, please contact:

1. Your primary care physician: This is often your first point of contact to discuss your concerns and determine whether screening is appropriate for you based on your medical history and risk factors.
2. A gastroenterologist: A specialist in diseases of the gastrointestinal tract, can provide specific details about the different types of screening available and which might be best for you.
3. Cancer Prevention Centers: Specialized facilities for the prevention and early diagnosis of tumors can offer specific consultations and screening programs.
4. Local Clinics or Hospitals: Many hospitals offer colon cancer screening programs and have staff available to answer questions.
5. Public Health Services: They can provide educational resources and information about screening programs available in your area.
6. Cancer Associations: Non-profit organizations, such as the Italian League for the Fight against Cancer (LILT), offer information and support for cancer prevention.

Consulting with a knowledgeable health professional will help you better understand the screening options available and make informed decisions about your health.

OUTPUT (SESSION 2): To learn more about colon cancer screening, you can turn to several reliable sources:

1. Primary Care Physician: Your first port of call should be your family doctor or GP, who can provide personalized advice based on your medical and family history.
2. Gastroenterology Specialist: A gastroenterologist is a doctor who specializes in disorders of the gastrointestinal tract and can offer specific details about the screenings and tests that are best for you.
3. Cancer Screening Centers: Many hospitals and clinics offer cancer screening programs and have trained staff who can provide you with detailed information.
4. Local public health departments: These agencies often have resources and informational materials about available screening programs and current recommendations.
5. Cancer Associations: Industry organizations, such as the Italian Association for Cancer Research (AIRC) or similar, provide educational resources and detailed information on the benefits of screening and research developments.

By consulting these sources, you can get a comprehensive overview of colon cancer screening and the options available to you.

Text similarity: 83%

2) **Who should I contact to participate in colon cancer screening?**

OUTPUT (SESSION 1): To participate in colon cancer screening, you should contact:

1. Your GP: Can assess your risk, provide you with a screening referral, and give you information about how to proceed.
2. A gastroenterologist: This specialist can give you detailed advice on available screening tests and book any necessary tests, such as a colonoscopy.
3. Local Public Health Services: Many states or counties have colon cancer screening programs that you can contact directly for information and to schedule an appointment.
4. Local clinics or hospitals: These facilities often offer screening programs and can help you arrange for any necessary tests.
   Be sure to discuss with a healthcare professional your specific risk factors and which screening method is most appropriate for you.

OUTPUT (SESSION 2): To participate in colon cancer screening, you should contact:

1. Primary Care Physician: Your primary care physician is the first step in starting the screening process. They can provide you with the information you need and direct you to the appropriate screening services.
2. Regional Screening Program: In many regions, there are screening programs organized by the public health service. Contacting your local public health department or visiting your local health authority's website can help you find out how to participate.
3. Gastroenterologist: If you have specific risk factors, it may be helpful to contact a gastroenterology specialist who can offer more specific advice and suggest appropriate tests.
   By contacting one of these professionals or services, you can obtain the information you need to enroll in the screening program that best suits your needs.

Text similarity: 90%

3) INPUT: How can I participate in colon cancer screening?

OUTPUT (SESSION 1): To participate in colon cancer screening, you can follow these steps:

1. Consult your primary care physician: This is the first step in discussing your eligibility for screening. Your doctor will assess your risk factors and provide you with personalized recommendations, as well as explain the different types of tests available.
2. Get a prescription or referral : Your doctor may give you a prescription for the appropriate screening test or refer you to a specialist, such as a gastroenterologist, for further testing.
3. Contact a screening center or clinic: You can make an appointment at a specialized center, hospital, or clinic that offers screening programs for colon cancer.
4. Prepare for the test: Depending on the type of screening recommended (e.g., colonoscopy, fecal occult blood test), you may need to follow specific instructions, such as a special diet or taking laxatives.
5. Take the screening test: Follow the instructions provided by your doctor or screening center to complete the test.
6. Get your results and plan next steps: After your test, your doctor will discuss your results and any further action needed, which may include follow-up or additional treatments if needed.
   Regular screening, as directed by your doctor, is important to detect problems early and maintain your health.

OUTPUT (SESSION 2): To participate in colon cancer screening, follow these steps:

1. Contact your GP: Start by talking to your GP. They can advise you on the appropriate timing for screening based on your age, family history and personal risk factors.
2. About Regional Screening Programs: In many regions, public health services offer organized screening programs for colon cancer. You can often find information on the websites of local health departments or by contacting your local public health department to find out how to participate.
3. Receive an invitation: In organized programs, you may receive a direct invitation to participate in screening. Be sure to follow the instructions provided to book your appointment or collect your test kits if it is a fecal-based screening.
4. Take the test: Follow the instructions provided to complete the screening test, which may include procedures such as a fecal occult blood test or a colonoscopy, depending on your program and your risk factors.
5. Get Your Results: Once your screening is complete, be sure to get your results and discuss them with your doctor to figure out next steps, if any.

Participating in colon cancer screening is an important preventative step in maintaining your health and should be done regularly according to appropriate medical guidelines.

Text similarity: 86%

4) **INPUT: How do I access colon cancer screening?**

OUTPUT (SESSION 1): Accessing colon cancer screening generally involves the following steps:

1. Consultation with Primary Care Physician: Talk to your primary care physician about your age, family history, and personal risk factors to get a screening recommendation. Your doctor can explain the different types of screening available and determine which is best for you.
2. Screening Plan: Based on your doctor's advice, your doctor will guide you on the most appropriate plan, which may include a referral for a colonoscopy or other tests such as a fecal occult blood test (FOBT) or a fecal immunochemical test (FIT).
3. Booking an Appointment: Once you have your referral or prescription, contact a clinic, hospital, or screening center to book your test. Many public and private hospitals offer these services.
4. Preparing for the Test: Depending on the type of screening, you may be given specific instructions. For example, for a colonoscopy, you may need to follow a special diet and take laxatives to clean your bowels.
5. Screening Test: Show up for your scheduled appointment to get tested. Follow all instructions from your healthcare provider to ensure the test is performed correctly.
6. Results and Follow-Up: After the test, your GP will discuss the results with you and advise you on any next steps, which may include further monitoring or treatment if necessary.

Regular screening helps prevent colorectal cancer by detecting any abnormalities in their early stages.

OUTPUT (SESSION 2): To access colon cancer screening, follow these steps:

1. Consult your primary care physician: Start by talking to your family doctor, who can assess your personal and family risk factors and advise you on the timing and methods of screening that are best for you.

2. Learn about regional screening programs: Many regions offer screening programs organized by the public health service. You can get information about these programs by visiting your local health authority website or by contacting the public health department in your area.
3. Receive the invitation or contact the health services directly:
    - In some regions, you may receive an invitation to participate in the screening program when you reach the recommended age.
    - If you do not receive an invitation but would like to participate, you can contact your local health service to ask to be included in the program.
4. Take the test: Once you gain access to the program, you will receive detailed instructions on how to take the screening test, which may include collecting a fecal sample for occult blood or having a colonoscopy.
5. Follow the instructions and report the results: After taking the test, send the samples to the designated laboratory, if requested, and await the results. Your doctor will discuss the results and next steps with you, if necessary.

By participating in regular colon cancer screening, you can significantly reduce your risk of developing cancer and improve your chances of successful treatment if it is detected early.

Text similarity: 84%

5) **INPUT: Who should I contact to participate in colon cancer screening?**

OUTPUT (SESSION 1): To participate in colon cancer screening, you can contact:

1. General Practitioner: This is the recommended first point of contact. They can discuss your personal risk factors, explain the various screening options available, and provide a prescription or address for any necessary tests.
2. Gastroenterologist: This specialist can offer detailed advice on screening tests, such as colonoscopy, and directly manage the execution of these tests.
3. Local Screening Center: Many local hospitals and clinics have colon cancer screening programs and can assist you in scheduling and getting the appropriate tests.
4. Public Health Programs: In some areas, there are screening programs organized by local health authorities that offer free or reduced-cost testing, especially for people at risk.

Contacting one of these professionals or facilities will help you start the screening process and proactively manage your health.

OUTPUT (SESSION 2): To participate in colon cancer screening, you should contact:

1. Primary care doctor: Your family doctor is a great place to start. They can provide information about available screening programs and help you decide whether you are eligible for one based on your age, family history, and risk factors.
2. Local Health Services: Contact your local health department or public health department to find out about screening programs in your area. These programs are often offered by the public health service and are designed for individuals within a certain age range or with certain risk factors.
3. Screening Centers and Hospitals: Many hospitals offer colon cancer screening services. You can contact these facilities directly to learn about available tests, such as colonoscopy or fecal occult blood tests.

By asking your doctor or contacting local services, you can get the information you need to enroll in the program that's right for you and ensure you're following an appropriate screening plan.

Text similarity: 92%

6) **INPUT: Who is eligible for colon cancer screening?**

OUTPUT (SESSION 1): Colon cancer screening is primarily aimed at:

1. Adults aged 45 and older: This is the recommended start for screening in most guidelines, as the risk of colorectal cancer increases with age.
2. People with a family history of colorectal cancer or polyps: Those who have first-degree relatives (such as parents, siblings, or children) who have had the disease may need to start screening earlier and more often.
3. Individuals with inherited conditions: Those with genetic syndromes such as Lynch syndrome or familial adenomatous polyposis (FAP), which increase the risk of colon cancer, need earlier and more frequent screening.
4. People with inflammatory bowel disease: People with conditions such as ulcerative colitis or Crohn's disease are at increased risk and may need more frequent screening.
5. People who have had polyps or colon cancer in the past: These individuals need regular checkups to prevent recurrence or detect new growths early.
6. People with other risk factors: These include obesity, poor diet, long-term alcohol and tobacco use, and sedentary lifestyles, which can increase the risk of colon cancer.

It is important to discuss with a doctor which screening criteria apply to your specific case to ensure timely diagnosis and the best possible management.

OUTPUT (SESSION 2): Colon cancer screening is primarily intended for the following people:

1. Adults aged 45-50 years and older: International guidelines identify this age group as the starting point for routine screening programs, as the risk of developing colorectal cancer increases with age.
2. People with a family history of colon cancer: Anyone who has first-degree relatives (parents, siblings, or children) who have had colon cancer should start screening earlier than the recommended age for the general population, often about ten years younger than the age at which the family member was diagnosed.
3. Individuals with inherited genetic syndromes: Those with conditions such as familial adenomatous polyposis (FAP) or Lynch syndrome (hereditary nonpolyposis colorectal cancer) are at higher risk and require earlier and more frequent screening.
4. Patients with a personal history of colon polyps or cancer: Those who have already had precancerous polyps or colon cancer should undergo more regular checkups.
5. People with inflammatory bowel disease: People with conditions such as ulcerative colitis or Crohn's disease, which increase the risk of colon cancer, should get more frequent screenings.

Consulting with a doctor is essential to determine the right time and frequency for screening based on your personal risk factors.

Text similarity: 88%

7) **INPUT: At what age should colon cancer screening be started?**

OUTPUT (SESSION 1): The recommended age to start colon cancer screening is generally 45 years for adults at average risk. This recommendation has been lowered from the previous age of 50 years because of an increase in colorectal cancer cases among younger people. However, there are situations in which it may be appropriate to start screening earlier:

1. Family history: If you have a first-degree relative who has had colorectal cancer or adenomatous polyps, you may need to start screening earlier, often around age 40, or at an age that is 10 years younger than the age at which your relative was diagnosed, whichever comes first.
2. Hereditary conditions: People with high-risk inherited syndromes, such as Lynch syndrome or familial adenomatous polyposis (FAP), may require screening that begins in adolescence or shortly thereafter, based on their doctor's recommendation.
3. Inflammatory bowel disease: People with conditions such as ulcerative colitis or Crohn's disease may need closer monitoring starting earlier than the standard age recommended for those at average risk.

It is always important to discuss your specific case with a doctor to determine the best time to begin screening.

OUTPUT (SESSION 2): Colon cancer screening is generally recommended starting at age 45 for people at average risk. This age has been lowered from the previous recommendation of 50 years, due to an increased risk observed in younger age groups.

If you have higher risk factors, such as a family history of colon cancer, inherited genetic syndromes, or a personal history of polyps or inflammatory bowel disease, you may need to start screening even earlier. In these cases, it is important to consult with your doctor to determine a screening plan that is appropriate for your individual needs.

Text similarity: 85%

8) **INPUT: At what age should colon cancer screening be done?**

OUTPUT (SESSION 1): Screening for colon cancer is generally recommended starting at age 45 for adults at average risk. This guideline was established to improve early detection, given the increasing incidence of colorectal cancer among younger people. However, in some cases, it may be necessary to start earlier:

1. Family History: If you have first-degree relatives who have had colorectal cancer or polyps, it may be recommended to start screening earlier, around age 40 or 10 years earlier than the age your relative was diagnosed with the disease, whichever comes first.
2. Lynch Syndrome or FAP: People with inherited genetic syndromes that increase their risk may need screening much earlier, potentially as early as their late teens or early 20s.
3. High Risk Conditions: If you have chronic inflammatory bowel diseases, such as ulcerative colitis or Crohn's disease, your doctor may recommend starting screening before age 45.

It is advisable to discuss with your doctor to customize the screening plan based on your risk factors.

OUTPUT (SESSION 2): Colon cancer screening is generally recommended starting at age 45 for people at average risk. This recommendation reflects current guidelines, which have been adapted to respond to the increase in colorectal cancer cases observed in younger age groups.
Those with high risk factors, such as a family history of colon cancer, inherited genetic syndromes (such as Lynch syndrome), or conditions such as ulcerative colitis or Crohn's disease, may need to start screening earlier. It's important to talk to your doctor about tailoring a screening plan to your risk factors and history.

Text similarity: 82%

9) **INPUT: How often should colon cancer screening be done?**

OUTPUT (SESSION 1): The frequency of colon cancer screening depends on the type of test used and the individual patient's risk. Here are general recommendations:

1. Colonoscopy: Every 10 years, if results are normal and there are no other risk factors.
2. sigmoidoscopy : Every 5 years, or every 10 years if combined with an annual fecal immunochemical test (FIT).
3. Virtual colonoscopy (CT colonography): Every 5 years.
4. Fecal occult blood test (FOBT) or fecal immunochemical test (FIT): Yearly.
5. Stool DNA test (such as Cologuard): Every 3 years.

These are general guidelines for average-risk individuals. Those at higher risk, such as those with a family history of colorectal cancer or certain inherited conditions, may need more frequent screening or an earlier start. It is important to discuss with your doctor how often you should get screening to meet your needs.

OUTPUT (SESSION 2): The frequency of colon cancer screening depends on the type of test used and your personal risk factors. Here is a general guide:

1. Colonoscopy: If results are normal, it is generally recommended every 10 years for people at average risk.
2. sigmoidoscopy : In the absence of abnormalities, it is recommended to repeat it every 5 years, often in combination with fecal occult blood testing (FOBT) every 3 years.
3. Fecal occult blood test (FOBT) or fecal immunochemical test (FIT): These tests, which look for blood in the stool, are generally recommended annually.
4. Fecal DNA test: This test, which looks for genetic mutations in your stool, can be done every 3 years if the results are normal.

Frequency may vary for people with high risk factors, so it is important to discuss a personalized screening plan with your doctor.

Text similarity: 86%

10) **INPUT: How often should colon cancer screening be done?**

OUTPUT (SESSION 1): The frequency of colon cancer screening depends on the type of test chosen and individual risk factors. Here are general recommendations for a person at average risk:

1. Colonoscopy: Every 10 years, if results are normal.
2. sigmoidoscopy : Every 5 years, or combined with an annual fecal immunochemical test (FIT).
3. Virtual colonoscopy (CT colonography): Every 5 years.

4. Fecal occult blood test (FOBT) or Fecal immunochemical test (FIT): Every year.
5. Stool DNA test (such as Cologuard): Every 3 years.
   These intervals are based on general guidelines. People at higher risk, such as those with a family history of colorectal cancer or specific inherited conditions, may need more frequent screening or may start earlier. It is always a good idea to consult with your doctor to determine the most appropriate screening schedule for your situation.

   OUTPUT (SESSION 2): How often you should screen for colon cancer depends on the type of test you choose and your individual risk factors. Here is a general overview:

1. Colonoscopy: If results are normal, it is usually recommended every 10 years for people at average risk.
2. Sigmoidoscopy : Generally recommended every 5 years, often combined with an annual fecal occult blood test (FOBT) or FIT.
3. Fecal occult blood test (FOBT) or fecal immunochemical test (FIT): Recommended annually if used as the primary screening method.
4. Fecal DNA testing: Usually recommended every 3 years in the absence of abnormalities.
   These guidelines may vary for people with high risk factors, such as a personal or family history of polyps or colon cancer. In these cases, it is important to consult with your doctor for a personalized screening program.

   Text similarity : 85%

SCENARIO 3
You have received a positive fecal occult blood test result but are unsure of its implications and what other tests you may need. Ask your question for clarification.

1) **INPUT: Does a positive fecal occult blood test result mean I have cancer?**

   OUTPUT (SESSION 1): A positive fecal occult blood test (FOBT or FIT) does not necessarily mean that you have cancer. This result indicates that blood has been detected in your stool, but blood can come from a variety of sources. In addition to colon cancer, other causes may include:

- Non-cancerous polyps in the colon.
- Hemorrhoids.
- Ulcerations or inflammation of the intestines, such as in Crohn's disease or ulcerative colitis.
- Dietary changes or taking certain medications.
  If your fecal occult blood test is positive, your doctor will likely recommend further evaluation, usually with a colonoscopy, to determine the cause of the bleeding and check for polyps or tumors. It is important to follow your doctor's recommendations and not jump to conclusions based on a positive test result.

   OUTPUT (SESSION 2): A positive fecal occult blood test result does not necessarily mean that you have cancer. This test detects the presence of blood in the stool, which can result from a variety of causes, including:

- Polyps: Benign growths in the colon that may bleed.
- Hemorrhoids: Can cause bleeding.
- Inflammation or infection of the intestines: Conditions such as colitis can cause blood to appear.

- Other non-cancerous lesions: Ulcers or trauma may lead to bleeding.
  A positive result indicates the need for further testing, usually with a colonoscopy, to determine the cause of the bleeding. It is important to discuss the test results with your doctor, who can advise you on appropriate next steps.

  Text similarity: 83%

2) **INPUT: Does a positive occult blood test result mean you have colon cancer?**

   OUTPUT (SESSION 1): No, a positive fecal occult blood test does not necessarily mean you have colon cancer. A positive result indicates the presence of blood in your stool, which can be caused by a variety of things, not just cancer. Possible sources of bleeding include:

- Non-cancerous polyps.
- Hemorrhoids.
- Ulcerations or inflammation of the intestines, such as ulcerative colitis or Crohn's disease.
- Taking certain foods or medications that can cause bleeding.
  A positive result is a signal to perform further tests, usually a colonoscopy, to identify the cause of the bleeding and evaluate for polyps or tumors. It is essential to follow your doctor's instructions to obtain an accurate diagnosis and appropriate treatment.

  OUTPUT (SESSION 2): No, having a positive fecal occult blood test does not necessarily mean you have colon cancer. This test detects blood in the stool, which can be caused by a variety of conditions, not all of which are cancerous. For example:

- Noncancerous polyps: Some polyps can cause bleeding.
- Hemorrhoids: Can cause blood in the stool.
- Inflammation or infection: Such as those caused by colitis or other inflammatory bowel diseases.
- Ulcerations or other lesions: May lead to gastrointestinal bleeding.
  A positive result indicates that further investigation is needed to identify the cause of the bleeding, usually through a colonoscopy. It is important to discuss the results with your doctor to determine the appropriate next steps.

  Text similarity: 89%

3) **INPUT: After a positive fecal occult blood test result, do I need to do any other tests?**

   OUTPUT (SESSION 1): Yes, after a positive FOBT result, further testing is generally recommended to determine the cause of the bleeding. The most common next step is a colonoscopy.
   Colonoscopy is a procedure that allows your doctor to view the inside of your colon and rectum using a flexible tube with a camera. This test allows you to:

- Identify and remove any polyps that may be precancerous.
- Diagnose the presence of inflammation or other pathological conditions.
- If necessary, perform biopsies of any abnormal tissue for more detailed analysis.
  It is important to follow your doctor's recommendations for colonoscopy or other suggested tests in order to get an accurate diagnosis and decide on the best course of action.

OUTPUT (SESSION 2): Yes, after a positive fecal occult blood test result, it is important to do further testing to determine the cause of the bleeding. The most common procedure that is recommended is a colonoscopy.
Colonoscopy allows your doctor to directly examine the inside of your colon and rectum using a flexible tube with a video camera. During the procedure, your doctor may:

- Identify and remove polyps, if present.
- Take tissue samples (biopsy) for further testing of any abnormalities found.

This test will help clarify whether the cause of your bleeding is noncancerous, such as polyps or hemorrhoids, or due to a more serious condition, such as colon cancer. Discussion and planning with your doctor are essential to understanding your test results and determining the right next steps.

Text similarity: 87%

4) **INPUT: Can a positive fecal occult blood test result be a mistake?**

OUTPUT (SESSION 1): Yes, a positive fecal occult blood test result can be a false positive. This means that the test detected blood, but it may not be from a serious problem such as colon cancer. There are several factors that can affect the result, including:

- Diet: Consumption of certain foods (such as red meat, some fruits and vegetables) which can interfere with some types of FOBT tests.
- Medications: The use of nonsteroidal anti-inflammatory drugs (NSAIDs), aspirin, or anticoagulants may cause gastrointestinal bleeding.
- Benign Conditions: Disorders such as hemorrhoids or gastrointestinal ulcers can cause blood in the stool.

Because of the possibility of false positives, a positive fecal occult blood test usually requires further evaluation, often through a colonoscopy. This test can help determine the exact cause of the bleeding and rule out or confirm the presence of more serious conditions.

OUTPUT (SESSION 2): Yes, a positive fecal occult blood test result can be a false positive, which occurs when the test indicates the presence of blood but there is no serious condition such as cancer. There are several reasons why false positives can occur:

1. Diet: Certain foods, such as red meat, certain vegetables (e.g., radishes, cauliflower), or vitamin C and iron supplements, can affect test results.
2. Medications: The use of medications such as aspirin or other nonsteroidal anti-inflammatory drugs (NSAIDs) can cause gastrointestinal bleeding, affecting the results.
3. Non-cancerous conditions: Bleeding from hemorrhoids, anal fissures, or inflammatory bowel disease may yield a positive result.

Because of these potential impacts on the results, a positive test is usually followed by a colonoscopy to directly examine the colon and determine the exact cause of the bleeding. It is essential to discuss the results with your doctor to determine the appropriate next steps.

Text similarity: 86%

5) **INPUT: After a positive fecal occult blood test result, do I need to do another confirmatory fecal occult blood test?**

OUTPUT (SESSION 1): Typically, after a positive FOBT result, the next step is not to perform another confirmatory test, but to undergo a colonoscopy. Colonoscopy is considered the gold standard for further investigation, as it provides direct visualization of the inside of the colon and rectum to identify the source of the bleeding.
A colonoscopy allows you to:

- Identify polyps, tumors, or other abnormalities.
- Perform biopsies of suspicious tissues.
- Remove potentially precancerous polyps.

Repeating the occult blood test without a colonoscopy may delay the diagnosis of any conditions that require medical attention. Therefore, it is important to follow your doctor's instructions and schedule a colonoscopy to get an accurate evaluation.

OUTPUT (SESSION 2): After a positive fecal occult blood test result, another confirmatory occult blood test is not usually performed. Instead, the recommended next step is usually a colonoscopy.
Colonoscopy is important because it allows you to directly examine the inside of the colon and rectum to identify the cause of bleeding. Through this procedure, it is possible not only to diagnose the presence of polyps or other abnormalities, but also to intervene immediately, for example by removing polyps that could be precancerous.
Performing another occult blood test, however, may not provide additional useful information and may delay the opportunity to obtain an accurate diagnosis. It is essential to follow your doctor's instructions for next steps after a positive result.

Text similarity: 85%

6) **INPUT: Can a positive FOBT result be caused by hemorrhoids?**

OUTPUT (SESSION 1): Yes, a positive FOBT test result can be caused by hemorrhoids. Hemorrhoids are considered a benign cause of rectal bleeding and can lead to blood in the stool, which the test can detect. However, because FOBT can also indicate other, more serious conditions such as polyps or tumors in the colon or rectum, it is important not to jump to conclusions based on this result alone.
A positive result requires further testing, often through a colonoscopy, to determine the exact cause of the bleeding and ensure there are no more serious conditions that have gone undiagnosed. Following your doctor’s instructions is essential to receiving an accurate diagnosis and proper treatment.

OUTPUT (SESSION 2): Yes, a positive FOBT result can be caused by hemorrhoids. Hemorrhoids are enlarged veins in the anal and rectal regions that can cause bleeding. This bleeding can contaminate the stool and lead to a positive FOBT result. However, a positive result could also mean the presence of other conditions, some of which could be more serious, such as precancerous polyps or colon cancer. For

this reason, a positive result is usually followed by further investigation, such as a colonoscopy, to determine the exact cause of the bleeding.
It is important to discuss your test results with your doctor to understand what they mean for you and to plan any additional diagnostic tests that may be needed.

Text similarity: 89%

7) **INPUT: Can hemorrhoids justify a positive fecal occult blood test?**

OUTPUT (SESSION 1): Yes, hemorrhoids can cause a positive FOBT result. Hemorrhoids are swollen veins in the rectum or anus that can bleed, especially during or after bowel movements, and this bleeding can be detected by the test.
However, because a positive occult blood test result can also indicate other, more serious conditions, such as polyps or colorectal cancer, it is important that a positive result be followed up with further testing. Typically, the next step is a colonoscopy, which allows the inside of the colon and rectum to be directly examined to determine the exact cause of the bleeding.
It is important to follow medical recommendations to ensure an accurate diagnosis and effectively address any underlying health issues.

OUTPUT (SESSION 2): Yes, hemorrhoids can warrant a positive fecal occult blood test. Hemorrhoids are swollen veins in the anal and rectal region that can rupture and cause bleeding, which can be detected by a fecal occult blood test.
However, because a positive test can also indicate other conditions, such as polyps or colon cancer, it is important to do further testing to determine the exact cause of the bleeding. Typically, the next recommended step is a colonoscopy, which allows direct visualization of the inside of the colon and rectum to arrive at an accurate diagnosis.
Discussing your results with your doctor is essential for planning appropriate management and follow-up tests.

Text similarity: 84%

8) **INPUT: What does it mean to test positive for occult blood in the fecal matter?**

OUTPUT (SESSION 1): A positive fecal occult blood test means that blood has been detected in your stool by the test. This blood is not visible to the naked eye, but the test can identify it. A positive result does not necessarily mean cancer is present, but it does indicate that there is bleeding in the lower gastrointestinal tract that requires further investigation.
Possible causes of bleeding include:

- Colon polyps: Noncancerous growths that may bleed.
- Colorectal Cancer: One of the most serious possible causes.
- Hemorrhoids: Rectal varices that can cause bleeding.
- Ulcers or inflammation: Conditions such as ulcerative colitis or Crohn's disease.
- Diverticulosis: Small protrusions in the tissues of the colon that may bleed.

Following a positive result, it is common for a doctor to recommend a colonoscopy to more closely examine the inside of the colon and rectum, determine the source of the bleeding, and take appropriate action. It is important to follow your doctor's instructions to complete the diagnostic process.

OUTPUT (SESSION 2): A positive fecal occult blood test means that blood has been detected in your stool. This test is designed to identify traces of blood that are not visible to the naked eye.
A positive result indicates that there may be bleeding in the gastrointestinal tract, which can be caused by a variety of conditions, including:

- Polyps: Growths that may be precancerous and sometimes bleed.
- Hemorrhoids: Swollen veins in the rectal or anal area that may cause bleeding.
- Ulcers or inflammation: Conditions such as ulcerative colitis or other intestinal inflammation.
- Tumors: Both benign and malignant, which may bleed.

It is important to note that a positive result does not automatically mean that cancer is present. However, it is a sign that further investigation, typically through a colonoscopy, is warranted to determine the specific cause of the bleeding. Consulting with your doctor to discuss your results and next steps is essential.

Text similarity: 82%

9) **INPUT: I tested positive for fecal occult blood, what should I do?**

OUTPUT (SESSION 1): If you have a positive FOBT test, the most commonly recommended next step is to have a colonoscopy. A colonoscopy is a procedure that examines the inside of your colon and rectum to identify the cause of bleeding. Here's what you should do:

1. Contact your doctor: Tell your doctor about your test result. He or she will give you instructions on how to proceed.
2. Schedule a colonoscopy: Your doctor will refer you to a gastroenterologist to schedule a colonoscopy. This test is essential to determine if your bleeding is caused by polyps, cancer, or other conditions such as hemorrhoids or inflammation.
3. Prepare for the exam: Follow the instructions provided to prepare for the colonoscopy. This usually includes a special diet and laxatives to cleanse the colon.
4. Attend your appointment: Be sure to attend your colonoscopy appointment. The exam will help you get an accurate diagnosis and plan any necessary treatments.
5. Get your results and follow recommendations: After your colonoscopy, you will discuss your results with your doctor to determine next steps, which may include treatments, lifestyle changes, or further monitoring.

Addressing a positive result promptly and following your doctor's recommendations is important to ensure an accurate diagnosis and appropriate treatment.

OUTPUT (SESSION 2): If you have a positive FOBT test, the next recommended step is usually a colonoscopy. A colonoscopy is a more detailed exam that allows the doctor to view the inside of your colon and rectum. Through this exam, your doctor can:

- Identify and remove any polyps present.
- Perform biopsies on suspicious tissues for further examination.
- Rule out or confirm the presence of serious conditions, such as colon cancer.

Here's what you should do after a positive result:

1. Consult your primary care physician: Discuss your test results with your doctor to understand what they mean specifically for you and to get recommendations on how to proceed.

2. Schedule a Colonoscopy: Your doctor will likely refer you to a gastroenterologist to schedule a colonoscopy.
3. Prepare for the exam: Carefully follow the instructions provided to prepare for your colonoscopy, which may include a special diet and laxatives to cleanse your bowels.
4. Follow the next recommendations: Based on the results of your colonoscopy, your doctor will discuss next steps, which may include treatments or a schedule for future monitoring. Positive results do not necessarily mean the presence of cancer, but it is crucial to do further tests to fully understand the situation.

Text similarity: 85%

10) INPUT: What to do if the fecal occult blood test is positive?

OUTPUT (SESSION 1): If the fecal occult blood test is positive, it is important to take the following steps for a more thorough and accurate evaluation:

1. Contact your doctor: Inform your doctor of your positive result. It is essential to discuss the test results to better understand what it might mean for you.
2. Schedule a colonoscopy: Your doctor will probably recommend a colonoscopy. This procedure is essential to examine the inside of your colon and rectum to identify the source of bleeding.
3. Follow preparation instructions: Before your colonoscopy, you will need to follow specific instructions to prepare your bowel, which may include a special diet and the use of laxatives to cleanse your bowels.
4. Undergo a colonoscopy: This procedure will allow your doctor to see if there are polyps, tumors, or other sources of bleeding in your colon. It may also allow polyps to be removed or tissue samples to be taken for further analysis.
5. Interpreting the Results: After your colonoscopy, your doctor will discuss the results with you and advise you on next steps, which may include further monitoring, surgery, or lifestyle changes.

Addressing a positive result quickly and following medical advice is critical to ensure that any potential problems, if present, can be treated as effectively as possible.

OUTPUT (SESSION 2): If you have a positive fecal occult blood test result, it is important to follow these steps to further investigate the situation and determine the cause of the bleeding:

1. Consult your doctor: Talk to your primary care physician to discuss your test result. This is a crucial step in understanding what a positive result means and planning future follow-up.
2. Prepare for additional tests: Your doctor will probably recommend a colonoscopy. This test is the most effective way to examine the inside of your colon and rectum, identify the source of bleeding, and remove or biopsy polyps or other abnormalities.
3. Schedule your colonoscopy: Make an appointment with a gastroenterologist to have your colonoscopy done. Be sure to carefully follow all preparation instructions provided by your doctor to ensure your bowels are clean and the test is accurate.
4. Discussion of Results: After your colonoscopy, your doctor will discuss your results and next steps with you. If polyps or other conditions are identified, additional interventions or monitoring may be needed.

The goal of these steps is to identify or rule out serious health problems and ensure that you receive appropriate care as soon as possible.

Text similarity : 84%

SCENARIO 4
Following a positive fecal occult blood test result, you have been advised to undergo a colonoscopy and are unsure about the procedure and how to prepare. Ask your question for detailed information.

1) **INPUT: How is a colonoscopy performed?**

OUTPUT (SESSION 1): Colonoscopy is a medical procedure used to examine the inside of the colon and rectum. Here is how a colonoscopy is usually performed:

1. Bowel Preparation: Before the exam, it is necessary to completely cleanse the intestines. This usually involves following a special fiber-free diet the day before and taking strong laxatives the day before the exam. This preparation is essential to allow a clear visualization of the intestinal mucosa.
2. Fasting: You will need to avoid eating or drinking anything after midnight before your procedure unless otherwise directed by your doctor.
3. Sedation: Before the colonoscopy begins, you will be given sedative medications to help you relax and reduce discomfort. This means you will be very drowsy or asleep during the procedure, but not completely under general anesthesia.
4. Performing the Exam:
    - The doctor uses a colonoscope, a flexible tube equipped with a camera and light, which is inserted through the rectum and gently pushed into the colon.
    - Gas (usually air or CO2) may be introduced into the colon to distend it and allow better visualization of the colonic wall.
    - During the procedure, the doctor looks for polyps, tumors, or other abnormalities. Small polyps may be removed during the exam, and tissue samples may be taken for biopsy if necessary.
5. Post-procedure: After your colonoscopy, you will be kept under observation until the effects of the sedation wear off. You may feel some bloating or cramping from the air you have introduced, but these symptoms usually resolve quickly.
6. Results: Once you have recovered, your doctor will discuss the preliminary results with you. If tissue was taken for biopsy, it will take some time to get the lab results.
It is important to remember that you may need assistance with preparation and recovery, so it is best to plan ahead and have someone drive you home after the exam.

OUTPUT (SESSION 2): A colonoscopy is a medical procedure used to examine the inside of the colon and rectum. Here's how it's usually done:

1. Preparation: In the days before the colonoscopy, the patient must follow a specific diet (often liquid) and take laxatives to completely clean the intestines. It is essential to follow these instructions carefully to ensure that the colon is free of food debris that could obstruct the view during the examination.
2. Sedation: Shortly before the procedure begins, a form of sedation or light anesthesia is usually administered to the patient to reduce discomfort and anxiety. The level of sedation may vary depending on the individual's needs and the practices of the healthcare facility.
3. Procedure: A colonoscope, a flexible tube with a camera on the end, is gently inserted through the anus and guided down the rectum and colon. The doctor may inflate the colon with air or carbon dioxide to better visualize the intestinal walls.
4. Examination and Procedures: During a colonoscopy, your doctor looks at the image from the camera to see if you can detect polyps, tumors, or other abnormalities. If

polyps are found, they are often removed during the procedure. Tissue samples (biopsies) may be taken for further analysis in a laboratory.

5. Conclusion and Recovery: After the procedure, the colonoscope is removed and the patient is monitored while the sedation wears off. You may experience a slight feeling of bloating or cramping from the air introduced during the exam, but this should subside quickly.
6. Results: Your doctor will discuss the results of your colonoscopy. If polyps were removed or biopsies were taken, the results may take a few days to come back.

Colonoscopy is a safe and highly effective test for the early diagnosis and prevention of colon cancer and other gastrointestinal conditions.

Text similarity: 89%

2) INPUT: What exactly does a colonoscopy consist of?

OUTPUT (SESSION 1): Colonoscopy is a diagnostic procedure that allows the entire colon and rectum to be examined for abnormalities, polyps or diseases such as cancer. Here is what it consists of exactly:

1. Bowel Preparation: Before the exam, it is essential to completely clean the intestine to ensure good visibility. This usually involves:
   - Follow a low-fiber diet in the days leading up to the test.
   - Take strong laxatives the day before your colonoscopy to empty your bowels.
   - Fast from solids and clear liquids at least from midnight before the day of the exam, following the doctor's instructions.
2. Sedation: Before the procedure begins, the patient is given sedation to reduce discomfort and anxiety. This semi-conscious state allows the patient to feel no pain during the examination.
3. Performing a colonoscopy:
   - A colonoscope, which is a thin, flexible tube equipped with a camera and light, is gently inserted through the rectum.
   - The colonoscope is slowly advanced through the colon, allowing the doctor to view the entire intestinal lining on the monitor.
   - Air or carbon dioxide is often blown into the colon to distend it and improve visibility and access.
4. Evaluation and Interventions: During the exam, the doctor looks for polyps, tumors, or other abnormalities. If polyps or suspicious tissue are found, they may be:
   - Removed.
   - Biopsied, that is, taken for further analysis in the laboratory.
5. Post-procedure: After the exam, the patient is monitored until the effects of the sedation wear off. Swelling or mild cramping may be felt, but these symptoms usually resolve quickly.
6. Discussion of Results: After the procedure, your doctor will discuss the preliminary results. If biopsies or polyp removals were done, complete lab results may take a few days.

Colonoscopy is a key test for the prevention of colorectal cancer and for the diagnosis of other intestinal conditions.

OUTPUT (SESSION 2): Colonoscopy is a diagnostic procedure that allows the inside of the colon and rectum to be examined to identify any abnormalities, such as polyps, inflammation, or tumors. Here's what it consists of exactly:

1. Bowel preparation: In the days leading up to the colonoscopy, you will need to follow a special diet, often based on clear liquids, and take laxatives. This preparation is essential to ensure that the bowel is completely clean for proper visualization during the exam.
2. Sedation: Before starting the procedure, light sedation is usually administered to reduce discomfort and anxiety. This makes the procedure more tolerable for the patient.
3. Performing the Procedure: During a colonoscopy, a flexible tube called a colonoscope, equipped with a video camera and a light source, is gently inserted into the anus and advanced through the rectum and colon.
4. Colon inspection: The doctor watches the video camera image on a monitor to look for any abnormalities. During the procedure, the colon may be slightly inflated with air or carbon dioxide to improve visualization of the intestinal walls.
5. Polyp Removal and Biopsies: If polyps or other abnormalities are found, they may be removed or biopsied (small tissue samples taken) for later analysis in a laboratory.
6. Conclusion and recovery: Once the examination is completed, the colonoscope is withdrawn. The patient is then kept under observation for a short time while the sedation wears off. There may be a temporary feeling of bloating or abdominal cramping, due to air being introduced into the colon.
7. Discussion of Results: Your doctor will immediately discuss the preliminary results with you and provide further details once the results of any biopsies are available.
   Colonoscopy is an essential tool for the prevention and early diagnosis of colorectal cancer, and is also useful for evaluating other intestinal diseases.

   Text similarity: 85%

3) **INPUT: Is colonoscopy a risky test?**

   OUTPUT (SESSION 1): Colonoscopy is generally considered a safe procedure and is routinely performed to screen for and diagnose colon conditions, including colorectal cancer. However, as with any medical procedure, it does carry some risks. Here are the potential risks associated with colonoscopy:

1. Colon Perforation: Although rare, there is a small risk that the colonoscope could perforate the wall of the colon, which would require surgery to repair.
2. Bleeding: Bleeding may occur, especially if polyps are removed or biopsies are taken. This type of bleeding is usually minor and resolves on its own or can be controlled during the procedure.
3. Sedation Reactions: Side effects from sedation may include breathing problems or adverse reactions, but these are rare. Before the procedure, your medical team will evaluate your health to minimize this risk.
4. Infections: Although very rare, local infections may occur following the procedure.
5. Abdominal pain or discomfort: It is common to feel some cramping or bloating after the procedure due to air being introduced into the colon, but these symptoms usually disappear quickly.
   It is important to discuss with your doctor the specific benefits and risks of colonoscopy for you, especially if you have any medical conditions or specific concerns. However, for most people, the benefits of screening and early detection of colorectal cancer through colonoscopy far outweigh the potential risks.

   OUTPUT (SESSION 2): Colonoscopy is considered a safe and effective procedure for the diagnosis and prevention of colorectal cancer and other intestinal diseases.

However, as with any medical procedure, there are some risks associated with it. Here are the main ones:

1. Bowel Perforation: A rare but serious risk during a colonoscopy is a perforation of the intestinal wall. This is very rare and often requires surgery to repair.
2. Bleeding: Bleeding may occur after a polyp is removed or biopsied. This bleeding is usually mild and resolves on its own, but in some cases, treatment may be necessary.
3. Sedation Reactions: Although rare, some people may have adverse reactions to medications used for sedation.
4. Infections: Although extremely rare, there is a small risk of infections following the procedure.

It is important to discuss these risks with your doctor before the procedure. Despite the potential risks, the benefits of colonoscopy, in terms of early detection and prevention of colon cancer, far outweigh the risks associated in most cases. Your doctor can help you weigh the benefits and risks personally and determine if colonoscopy is right for you.

Text similarity: 83%

4) **INPUT: Is colonoscopy a painful procedure?**

OUTPUT (SESSION 1): Colonoscopy is generally well tolerated by most patients and is not considered a painful procedure, due to the use of sedatives and analgesics. Here's what to expect regarding pain and discomfort associated with colonoscopy:

1. Sedation: During a colonoscopy, sedatives and, in some cases, pain relievers are given to help patients relax and feel less pain. Most people are drowsy or do not remember much of the procedure.
2. Minimal discomfort: Some patients may experience mild discomfort or abdominal cramping during or after the procedure, primarily due to the air being introduced into the colon to distend it and improve visibility.
3. Feeling of bloating: It is common to feel some swelling or pressure during and immediately after the procedure, but these symptoms usually pass quickly once the air is expelled.
4. Rapid recovery: After the procedure, the patient is monitored until the effects of the sedation wear off, usually within a couple of hours.

For many people, the anticipation of the procedure is often more stressful than the procedure itself, thanks to the comfort measures provided by the anesthesiologist and medical staff. It is important to share any concerns about pain or discomfort with your doctor before your colonoscopy so that he or she can tailor your care to your needs.

OUTPUT (SESSION 2): Colonoscopy is generally well tolerated and is not considered a painful procedure thanks to the use of sedatives and/or analgesics administered before and during the examination. These drugs help to relax the patient and reduce any discomfort.

During the procedure, some patients may experience:

- Feeling of pressure or fullness: This may be due to the introduction of air or carbon dioxide into the colon to expand the walls and improve visibility.
- Mild cramping: You may experience temporary cramping as the colonoscope is advanced or when air is introduced.

Most people who have a colonoscopy remember little or nothing about the procedure because of the sedation, and recovery from sedation is usually quick. It is important

to discuss any concerns about pain or discomfort with your doctor so that they can be addressed appropriately and you can be as comfortable as possible during the procedure.

Text similarity: 89%

5) **INPUT: Is colonoscopy an invasive procedure?**

OUTPUT (SESSION 1): Yes, a colonoscopy is considered an invasive procedure. It involves inserting a colonoscope, a flexible tube with a camera and light, through the rectum to examine the entire colon and rectum. Because it involves inserting instruments into the body, it is classified as invasive.
Despite this, colonoscopy is a standard procedure for detecting and preventing serious conditions, such as colorectal cancer, and for identifying polyps that may be precancerous. Because of the use of sedation, the procedure is generally well tolerated, with most patients experiencing minimal discomfort. The benefits of colonoscopy, in terms of early diagnosis and the ability to promptly remove potential risks, tend to outweigh the risks associated with its invasive nature.

OUTPUT (SESSION 2): Yes, a colonoscopy is considered an invasive procedure because it involves inserting a colonoscope—a flexible tube with a video camera on the end—through the anus and into the colon to examine the inside of the intestinal tract. Despite the invasive nature of the procedure, colonoscopy is generally safe and well-tolerated, largely because of the use of sedation or light anesthesia, which helps minimize discomfort and anxiety for the patient.
Its invasiveness is significantly offset by the benefits it offers, such as the possibility of:

- Identify and remove precancerous polyps.
- Quickly diagnose potentially serious conditions, such as colon cancer.
- Examine changes or abnormalities in the intestinal mucosa that may not be visible with other, less invasive tests.

Your doctor will assess your individual potential risks and discuss with you whether a colonoscopy is the best procedure for your medical needs and what preparation is needed to ensure a safe and accurate exam.

Text similarity: 89%

6) **INPUT: How should I prepare for a colonoscopy?**

OUTPUT (SESSION 1): Preparing for a colonoscopy is a critical part of the procedure to ensure that your colon is completely clean, allowing your doctor to clearly view the inside of your intestine. Here are typical steps to prepare for your colonoscopy:

1. Consult Your Doctor: Meet with your doctor for specific instructions. Each doctor or health care facility may have slightly different guidelines.
2. Low-residue diet: In the days leading up to your colonoscopy, you may be advised to follow a low-fiber diet. The day before the procedure, a clear liquid diet is usually required, such as broths, juices without pulp, tea, coffee without milk, and gelatin (not red or purple).
3. Taking laxatives: You will be prescribed a special laxative to cleanse your bowels. This may be a solution to drink, which leads to numerous bowel movements the day

before the test. It is important to carefully follow the instructions on when and how to take laxatives.

4. Fasting: Do not eat or drink anything after a certain time the evening before the test, usually midnight, unless otherwise directed by your doctor.
5. Medications: Discuss your current medication list with your doctor. You may need to stop or adjust certain medications, especially those that affect blood clotting or iron supplements.
6. Plan Transportation: Arrange for someone to drive you home after the procedure, as sedation will impair your ability to drive.

Following these instructions carefully is crucial to the success of your colonoscopy. Inadequate preparation may result in an incomplete view, requiring a repeat examination. If you have any questions or difficulty following the instructions, contact your doctor for assistance.

OUTPUT (SESSION 2): Preparing for a colonoscopy is essential to ensure that the bowel is completely clean, allowing the doctor to obtain a clear view of the inside of the colon. Here are typical steps to properly prepare:

1. Special Diet: In the days leading up to the test, you may be asked to follow a low-fiber diet and avoid solid foods the day before the procedure. The day before the test, a clear liquid diet is usually recommended, which may include:
   - Fat-free broth
   - Fruit juices without pulp (such as apple juice)
   - Jelly
   - Tea or coffee (without milk or cream)
   - Waterfall
2. Taking laxatives: Your doctor will prescribe a special laxative to take the night before your colonoscopy (and sometimes the morning of the procedure). This helps to completely clean your bowels. It is important to follow the instructions exactly.
3. Hydration: It is essential to drink plenty of fluids to avoid dehydration from laxatives. Drink clear fluids such as water, broth, and clear juices up to a couple of hours before the test, as directed by your doctor.
4. Medications: Consult your doctor about what medications you can continue to take. Some medications, especially blood thinners and anti-inflammatory drugs, may need to be adjusted.
5. Logistical Planning: Because sedation will be used, you will need someone to drive you home after the procedure. Plan for an afternoon off from work or other activities, as you may feel dizzy or fatigued.

Be sure to follow the specific instructions provided by your doctor or healthcare facility, as recommendations may vary. Careful preparation is essential for a safe and effective exam.

Text similarity: 90%

7) **INPUT: What does colonoscopy preparation consist of?**

OUTPUT (SESSION 1): Colonoscopy preparation is an essential process to completely clean the colon, so that the doctor can clearly examine its internal lining. Here is what the preparation typically consists of:

1. Low-residue diet: In the days leading up to your colonoscopy, you may be advised to follow a low-fiber diet to limit the buildup of residue in your colon.

2. Clear Liquid Diet: The day before your test, you will need to switch to a completely clear liquid diet. This may include:
   - Clear broths without solids.
   - Clear drinks such as tea, coffee without milk and clear fruit juices without pulp.
   - Jellies not red or purple.
   - Water and clear sports drinks.
3. Taking laxatives: You will be prescribed a specific laxative solution to cleanse your bowels. This usually involves:
   - Drink a large amount of liquid laxative the night before your colonoscopy, which causes frequent bowel movements to completely empty your colon.
   - Sometimes it may be necessary to drink an additional dose on the morning of the test.
4. Fasting: Usually, you are not allowed to eat or drink anything, except small sips of water to take medications, after midnight before the test.
5. Medications: Talk to your doctor about the medications you are taking. You may need to stop or change some of them, especially those that affect blood clotting.
6. Specific rules: Follow all specific directions provided by your doctor or health center where your colonoscopy will be performed, as some details may vary.

   It is important to follow these instructions carefully to ensure a clear and accurate view during your colonoscopy. An incompletely cleansed colon may result in an incomplete procedure, requiring a repeat colonoscopy. If you have any questions or are having difficulty with the preparation process, contact your doctor for assistance.

OUTPUT (SESSION 2): Colonoscopy preparation is a critical process to ensure that the bowel is clean and clearly visible during the procedure. Here is how the preparation typically works:

1. Low-residue diet: A few days before the test, your doctor may recommend that you follow a low-fiber diet to reduce the amount of residue in your intestines.
2. Clear Liquid Diet: The day before your colonoscopy, you will be switched to a diet of only clear liquids. This includes:
   - Fat-free broth
   - Fruit juices without pulp (such as apple juice or white grape juice)
   - Gelatin without red or purple colorants
   - Clear sports drinks
   - Waterfall
   - Tea or coffee (without milk or cream)
3. Taking laxatives: Your doctor will prescribe a specific laxative, which helps to completely empty your bowels. This may be a solution to drink in two parts: one the night before and another the morning of the procedure, depending on your doctor's instructions.
4. Hydration: It is important to stay well hydrated by drinking plenty of clear fluids. This also helps to compensate for fluids lost during the bowel cleansing phase.
5. Medications: Talk to your doctor about any medications you take regularly. You may need to temporarily stop taking some medications, such as those that affect blood clotting.
6. Travel Planning: Because you will be sedated during your colonoscopy, you will need someone to drive you home after the exam.

   Communicating any questions or concerns with your doctor is important to prepare effectively and to ensure that your colonoscopy is conducted safely and optimally.

Text similarity: 86%

8) **INPUT: How does the colonoscopy preparation take place?**

OUTPUT (SESSION 1): Colonoscopy preparation is essential to ensure that the doctor has a clear view of the colon. Preparation usually includes the following steps:

1. Low-residue diet: A few days before the test, your doctor may recommend a low-fiber diet to reduce the amount of stool in your colon.
2. Liquid diet: The day before your colonoscopy, you will be asked to follow a clear liquids only diet. This includes water, broth, strained fruit juices (without pulp), tea, coffee (without milk or cream), and gelatin (without red or purple dyes).
3. Taking laxatives: Usually, your doctor will prescribe a strong laxative to take the day before the test. This helps to empty your bowels completely. The type of laxative and the dosage depend on your doctor's specific instructions.
4. Hydration: It is important to drink plenty of clear fluids during preparation to prevent dehydration.
5. Medication changes: Talk to your doctor about any medications you are taking. You may need to stop or adjust some of them, especially if you take blood thinners or diabetes medications.
   Follow your doctor's instructions carefully to ensure that the test is performed correctly. If you have any questions or encounter difficulties during the preparation, contact your doctor for clarification.

OUTPUT (SESSION 2): Colonoscopy preparation is essential to ensure that the intestine is completely clean and free of debris, thus allowing optimal visibility during the examination. Here is an overview of how the preparation takes place:

1. Special diet:
   - Days before: In the days before your colonoscopy, you may be advised to follow a low-residue diet, reducing your fiber intake.
   - The day before: Switch to a clear liquid diet. This includes consuming only:
     - Clear broth (fat-free)
     - Clear fruit juices without pulp (such as apple juice)
     - Gelatin (avoiding red and purple colors)
     - Tea or coffee (without milk or cream)
     - Clear Sports Drinks
     - Waterfall
2. Taking laxatives:
   - Laxative Solution: Your doctor will prescribe a laxative solution, which should be taken according to specific instructions. It is often divided into two doses: one the night before and one early in the morning on the day of the test.
   - Laxative tablets (in some protocols): These may be prescribed to be taken together with the laxative solution.
3. Hydration:
   - It is important to drink plenty of clear fluids during preparation to avoid dehydration and to aid in cleansing the intestines.
4. Dressings:
   - Consult your doctor about any medications you are taking. You may need to temporarily stop or change your medications, especially those that affect blood clotting.
5. Further instructions and tips:
   - The effects of the laxative will begin to show within a few hours of taking it, so make sure you have access to a bathroom.
   - Wear comfortable clothes and plan to stay home during this phase.

By carefully following your doctor's instructions for preparation, you help ensure that the procedure is as effective and safe as possible. If you have any questions or concerns, be sure to discuss them with your doctor before your colonoscopy.

Text similarity: 93%

9) **INPUT: To undergo a colonoscopy, do I have to follow any dietary instructions in addition to taking the laxative?**

OUTPUT (SESSION 1): Yes, in addition to taking a laxative, it is important to follow specific dietary instructions to properly prepare for a colonoscopy. Here is what is generally recommended:

1. A few days before the exam:
   - Start a low-fiber diet to reduce the amount of stool.
2. The day before the colonoscopy:
   - Follow a diet of only clear liquids. This includes:
     - Waterfall
     - Broth
     - Strained fruit juices (such as apple or white grape, without pulp)
     - Sports drinks
     - Gelatin (without red or purple colouring)
     - Tea and coffee (without milk or cream)
3. On the day of the exam:
   - Typically, you will be asked not to eat or drink anything after midnight, except for small amounts of water needed to take any medications.

It is crucial to follow the instructions provided by your doctor carefully, as inadequate preparation may affect the quality of the exam, making it difficult to identify any abnormalities. If you have specific questions or if the instructions are unclear, contact your doctor for further clarification.

OUTPUT (SESSION 2): Yes, to undergo a colonoscopy, it is essential to follow specific dietary instructions in addition to taking the laxative, to ensure that the bowel is completely clean. Here are the general steps:

1. Previous days:
   - Low-fiber diet: In the days leading up to the procedure, your doctor may recommend avoiding foods high in fiber, such as fresh fruits, raw vegetables, whole grains, and seeds. Reducing these foods helps minimize waste in your intestines.
2. The day before the colonoscopy:
   - Clear Liquid Diet: Switch to a diet consisting only of clear liquids. You can consume:
     - Clear broth (without pieces of meat or vegetables)
     - Fruit juices without pulp, such as apple juice or white grape juice
     - Clear gelatin (avoiding red and purple colors)
     - Clear and transparent sports drinks
     - Tea or coffee (without milk or cream)
     - Waterfall
3. Taking the laxative:
   - Follow your doctor's directions for taking the laxative, often starting it the afternoon or evening before the procedure.
4. The day of the colonoscopy:

- Continue drinking clear fluids up to a couple of hours before the test, as directed by your doctor, to stay hydrated.

These dietary instructions are essential to allow for effective bowel cleansing, thus ensuring an accurate and safe colonoscopy. Following the instructions of your doctor or healthcare professional is crucial to the success of the procedure.

Text similarity: 90%

10) INPUT: Is anesthesia used during colonoscopy?

OUTPUT (SESSION 1): Yes, during a colonoscopy, some form of sedation or anesthesia is usually administered to ensure comfort and reduce discomfort during the procedure. Here are the common options:

1. Conscious Sedation: This type of sedation relaxes you and relieves pain, while you remain conscious. It usually uses medications administered intravenously.
2. Deep Anesthesia: Puts you into a deep sleep during which you are unconscious. Again, the drugs are administered intravenously.
3. General anesthesia (rarely used): In some cases, you may be given general anesthesia, which puts you to a complete sleep, but this is less common for routine colonoscopies.

The type of sedation chosen will depend on several factors, including your general health, any allergies or past reactions to sedative medications, and the preferences of your doctor and patient. It is important to discuss the sedation options available to you with your doctor to ensure that you receive the appropriate level of comfort and safety during the exam.

OUTPUT (SESSION 2): Yes, during a colonoscopy, some form of sedation or light anesthesia is usually administered to ensure patient comfort and reduce discomfort. Here are the common options:

4. Conscious Sedation: This is the most common form of sedation used during a colonoscopy. Medications are given intravenously, helping the patient to relax and often forget the procedure. Although lightly sedated, patients may be able to respond to simple commands.
5. Deep Sedation: For some patients or based on the doctor's preference, deeper sedation may be used, where the patient is not aware of the procedure and does not feel any pain. Again, medications are administered intravenously.
6. General anesthesia: This option is rarely used for colonoscopy, but may be indicated in special cases when required by specific medical conditions or patient preferences.

The type of sedation used may depend on a variety of factors, including your general health, past experience, and the policies of your healthcare facility. It is important to discuss with your doctor which sedation options are available and appropriate for you before the procedure. After a colonoscopy with sedation, you will need to be escorted home, as the sedative effects may last for several hours.

Text similarity : 89%

Supplementary Table 2. Likert Scales for assessment of Accuracy, Completeness and Comprehensibility.

	**Accuracy rating by a 5-point Likert scale**
**1**	Completely incorrect
**2**	More incorrect than correct
**3**	Approximately equally correct and incorrect
**4**	More correct than incorrect
**5**	Completely correct
	**Completeness rating by a 5-point Likert scale**
**1**	Incomplete, don't addresses any aspects of the question
**2**	Incomplete, addresses some aspects of the question, but significant parts are missing or incomplete
**3**	Adequate, provides the minimum amount of information required to be considered complete
**4**	Almost comprehensive, addresses nearly all aspects, with some missing information
**5**	Comprehensive, addresses all aspects of the question and provides additional information or context beyond what was expected
	**Comprehensibility rating by a 5-point Likert scale**
**1**	Difficult to understand
**2**	Partly difficult to understand
**3**	Adequately understandable
**4**	Easy to understand
**5**	Very easy to understand
